# Supplementary material for: Dynamic interaction of MYC enhancer RNA with YEATS2 protein regulates MYC gene transcription in pancreatic cancer
Source: EMBO Rep. 2025 Apr 11;26(10):2519–44. doi: 10.1038/s44319-025-00446-0 (PMC12117045; doi:10.1038/s44319-025-00446-0)
Supplement: Supplementary file 1 — Table EV1 [file 44319_2025_446_MOESM1_ESM.docx]

**Table EV1:** **Chronic Pancreatitis (CP) and normal Patient demographics and clinical characteristics**

| **Serial No** | **Sample ID** | **Age** | **Gender** | **Hospital** | **Method** | **Confirmatory test** | **Treatment naïve** | **Smoker** | **Alcoholic** |  |
| --- | --- | --- | --- | --- | --- | --- | --- | --- | --- | --- |
| 1 | S-107 | 45 | Male | SSKM | Surgical resection | H&E staining | YES | YES | YES | CP |
| 2 | S-151 | 30 | Male | SSKM | Surgical resection | H&E staining | YES | YES | YES | CP |
| 3 | S-129 | 22 | Female | SSKM | Surgical resection | H&E staining | YES | NO | NO | CP |
| 4 | S-142 | 33 | Male | SSKM | Surgical resection | H&E staining | YES | YES | YES | CP |
| 5 | S-37 | 39 | Male | SSKM | Surgical resection | H&E staining | YES | YES | YES | CP |
| 6 | S-92 | 21 | Female | SSKM | Surgical resection | H&E staining | YES | NO | NO | CP |
| 7 | S-27 | 19 | Male | SSKM | Surgical resection | H&E staining | YES | YES | NO | CP |
|  |  |  |  |  |  |  |  |  |  |  |
| 1 | CN-1 | 43 | Female | CNCI | Surgical resection | H&E staining | YES | Tobacco chewer | NO | Normal |
| 2 | S-144 | 50 | Male | SSKM | Surgical resection | H&E staining | YES | YES | NO | Normal |
| 3 | R-30 | 55 | Male | R G KAR | Surgical resection | H&E staining | YES | YES | NO | Normal |
| 4 | S-156 | 42 | Female | SSKM | Surgical resection | H&E staining | YES | NO | NO | Normal |
| 5 | R-2 | 50 | Female | R G KAR | Surgical resection | H&E staining | YES | NO | NO | Normal |
| 6 | S-128 | 48 | Male | SSKM | Surgical resection | H&E staining | YES | NO | NO | Normal |
| 7 | S-108 | 48 | Male | SSKM | Surgical resection | H&E staining | YES | YES | NO | Normal |
