# Supplementary material for: Dynamic interaction of MYC enhancer RNA with YEATS2 protein regulates MYC gene transcription in pancreatic cancer
Source: EMBO Rep. 2025 Apr 11;26(10):2519–44. doi: 10.1038/s44319-025-00446-0 (PMC12117045; doi:10.1038/s44319-025-00446-0)
Supplement: Supplementary file 2 — Table EV2 [file 44319_2025_446_MOESM2_ESM.docx]

**Table EV2**: **Pancreatic Ductal Adenocarcinoma** (**PDAC) Patient demographics and clinical characteristics**.

| **Serial No.** | **Patient ID** | **Age (Years)** | **Gender** | **CA19-9 level (U/ml)** | **Diabetic or not** | **Differentiation** | **TNM grade** | **Stage** | **Hospital** | **Method** | **Confirmatory test** | **Treatment naïve** | **Smoker** | **Alcoholic** |
| --- | --- | --- | --- | --- | --- | --- | --- | --- | --- | --- | --- | --- | --- | --- |
| 1 | CN1 | 43 | Female | 20.8 | Non-diabetic | Moderately differentiated | T2 N0 M0 | II | CNCI | Surgical resection | H&E staining | YES | Tobacco chewer | NO |
| 2 | R2 | 50 | Female | NA | Non-diabetic | Moderately differentiated | T2 N0 M0 | II | R G KAR | Surgical resection | H&E staining | YES | NO | NO |
| 3 | S108 | 48 | Male | NA | Non-diabetic | Well differentiated | T2 N1 M0 | IIb | SSKM | Surgical resection | H&E staining | YES | YES | NO |
| 4 | S93 | 55 | Male | NA | Non-diabetic | Well differentiated | T2 N1 M0 | IIb | SSKM | Surgical resection | H&E staining | YES | YES | NO |
| 5 | S113 | 57 | Female | NA | Non-diabetic | Well differentiated | T2 N0 M0 | IIa | SSKM | Surgical resection | H&E staining | YES | NO | NO |
