# Supplementary material for: Dynamic interaction of MYC enhancer RNA with YEATS2 protein regulates MYC gene transcription in pancreatic cancer
Source: EMBO Rep. 2025 Apr 11;26(10):2519–44. doi: 10.1038/s44319-025-00446-0 (PMC12117045; doi:10.1038/s44319-025-00446-0)
Supplement: Supplementary file 3 — Table EV3 [file 44319_2025_446_MOESM3_ESM.docx]

**Table EV3: Comprehensive list of histone reader molecules.**

>BRD2

MLQNVTPHNKLPGEGNAGLLGLGPEAAAPGKRIRKPSLLYEGFESPTMASVPALQLTPANPPPPEVSNPK

KPGRVTNQLQYLHKVVMKALWKHQFAWPFRQPVDAVKLGLPDYHKIIKQPMDMGTIKRRLENNYYWAASE

CMQDFNTMFTNCYIYNKPTDDIVLMAQTLEKIFLQKVASMPQEEQELVVTIPKNSHKKGAKLAALQGSVT

SAHQVPAVSSVSHTALYTPPPEIPTTVLNIPHPSVISSPLLKSLHSAGPPLLAVTAAPPAQPLAKKKGVK

RKADTTTPTPTAILAPGSPASPPGSLEPKAARLPPMRRESGRPIKPPRKDLPDSQQQHQSSKKGKLSEQL

KHCNGILKELLSKKHAAYAWPFYKPVDASALGLHDYHDIIKHPMDLSTVKRKMENRDYRDAQEFAADVRL

MFSNCYKYNPPDHDVVAMARKLQDVFEFRYAKMPDEPLEPGPLPVSTAMPPGLAKSSSESSSEESSSESS

SEEEEEEDEEDEEEEESESSDSEEERAHRLAELQEQLRAVHEQLAALSQGPISKPKRKREKKEKKKKRKA

EKHRGRAGADEDDKGPRAPRPPQPKKSKKASGSGGGSAALGPSGFGPSGGSGTKLPKKATKTAPPALPTG

YDSEEEEESRPMSYDEKRQLSLDINKLPGEKLGRVVHIIQAREPSLRDSNPEEIEIDFETLKPSTLRELE

RYVLSCLRKKPRKPYTIKKPVGKTKEELALEKKRELEKRLQDVSGQLNSTKKPPKKANEKTESSSAQQVA

VSRLSASSSSSDSSSSSSSSSSSDTSDSDSG

>BRD3

MSTATTVAPAGIPATPGPVNPPPPEVSNPSKPGRKTNQLQYMQNVVVKTLWKHQFAWPFYQPVDAIKLNL

PDYHKIIKNPMDMGTIKKRLENNYYWSASECMQDFNTMFTNCYIYNKPTDDIVLMAQALEKIFLQKVAQM

PQEEVELLPPAPKGKGRKPAAGAQSAGTQQVAAVSSVSPATPFQSVPPTVSQTPVIAATPVPTITANVTS

VPVPPAAAPPPPATPIVPVVPPTPPVVKKKGVKRKADTTTPTTSAITASRSESPPPLSDPKQAKVVARRE

SGGRPIKPPKKDLEDGEVPQHAGKKGKLSEHLRYCDSILREMLSKKHAAYAWPFYKPVDAEALELHDYHD

IIKHPMDLSTVKRKMDGREYPDAQGFAADVRLMFSNCYKYNPPDHEVVAMARKLQDVFEMRFAKMPDEPV

EAPALPAPAAPMVSKGAESSRSSEESSSDSGSSDSEEERATRLAELQEQLKAVHEQLAALSQAPVNKPKK

KKEKKEKEKKKKDKEKEKEKHKVKAEEEKKAKVAPPAKQAQQKKAPAKKANSTTTAGRQLKKGGKQASAS

YDSEEEEEGLPMSYDEKRQLSLDINRLPGEKLGRVVHIIQSREPSLRDSNPDEIEIDFETLKPTTLRELE

RYVKSCLQKKQRKPFSASGKKQAAKSKEELAQEKKKELEKRLQDVSGQLSSSKKPARKEKPGSAPSGGPS

RLSSSSSSESGSSSSSGSSSDSSDSE

>BRD4

MSAESGPGTRLRNLPVMGDGLETSQMSTTQAQAQPQPANAASTNPPPPETSNPNKPKRQTNQLQYLLRVV

LKTLWKHQFAWPFQQPVDAVKLNLPDYYKIIKTPMDMGTIKKRLENNYYWNAQECIQDFNTMFTNCYIYN

KPGDDIVLMAEALEKLFLQKINELPTEETEIMIVQAKGRGRGRKETGTAKPGVSTVPNTTQASTPPQTQT

PQPNPPPVQATPHPFPAVTPDLIVQTPVMTVVPPQPLQTPPPVPPQPQPPPAPAPQPVQSHPPIIAATPQ

PVKTKKGVKRKADTTTPTTIDPIHEPPSLPPEPKTTKLGQRRESSRPVKPPKKDVPDSQQHPAPEKSSKV

SEQLKCCSGILKEMFAKKHAAYAWPFYKPVDVEALGLHDYCDIIKHPMDMSTIKSKLEAREYRDAQEFGA

DVRLMFSNCYKYNPPDHEVVAMARKLQDVFEMRFAKMPDEPEEPVVAVSSPAVPPPTKVVAPPSSSDSSS

DSSSDSDSSTDDSEEERAQRLAELQEQLKAVHEQLAALSQPQQNKPKKKEKDKKEKKKEKHKRKEEVEEN

KKSKAKEPPPKKTKKNNSSNSNVSKKEPAPMKSKPPPTYESEEEDKCKPMSYEEKRQLSLDINKLPGEKL

GRVVHIIQSREPSLKNSNPDEIEIDFETLKPSTLRELERYVTSCLRKKRKPQAEKVDVIAGSSKMKGFSS

SESESSSESSSSDSEDSETAFCTSGDFVSPGPSPYHSHVQCGRFREMLRWFLVDVEQTAAGQPHRQSAAG

PAITWAPAIAYPSPECARCCVGCS

>P300/CBP

MSEREERRFVEIPRESVRLMAESTGLELSDEVAALLAEDVCYRLREATQNSSQFMKHTKRRKLTVEDFNR

ALRWSSVEAVCGYGSQEALPMRPAREGELYFPEDREVNLVELALATNIPKGCAETAVRVHVSYLDGKGNL

APQGSVPSAVSSLTDDLLKYYHQVTRAVLGDDPQLMKVALQDLQTNSKIGALLPYFVYVVSGVKSVSHDL

EQLHRLLQVARSLFRNPHLCLGPYVRCLVGSVLYCVLEPLAASINPLNDHWTLRDGAALLLSHIFWTHGD

LVSGLYQHILLSLQKILADPVRPLCCHYGAVVGLHALGWKAVERVLYPHLSTYWTNLQAVLDDYSVSNAQ

VKADGHKVYGAILVAVERLLKMKAQAAEPNRGGPGGRGCRRLDDLPWDSLLFQESSSGGGAEPSFGSGLP

LPPGGAGPEDPSLSVTLADIYRELYAFFGDSLATRFGTGQPAPTAPRPPGDKKEPAAAPDSVRKMPQLTA

SAIVSPHGDESPRGSGGGGPASASGPAASESRPLPRVHRARGAPRQQGPGTGTRDVFQKSRFAPRGAPHF

RFIIAGRQAGRRCRGRLFQTAFPAPYGPSPASRYVQKLPMIGRTSRPARRWALSDYSLYLPL

>TAF1

MGPGCDLLLRTAATITAAAIMSDTDSDEDSAGGGPFSLAGFLFGNINGAGQLEGESVLDDECKKHLAGLG

ALGLGSLITELTANEELTGTDGALVNDEGWVRSTEDAVDYSDINEVAEDESRRYQQTMGSLQPLCHSDYD

EDDYDADCEDIDCKLMPPPPPPPGPMKKDKDQDSITGVSENGEGIILPSIIAPSSLASEKVDFSSSSDSE

SEMGPQEATQAESEDGKLTLPLAGIMQHDATKLLPSVTELFPEFRPGKVLRFLRLFGPGKNVPSVWRSAR

RKRKKKHRELIQEEQIQEVECSVESEVSQKSLWNYDYAPPPPPEQCLSDDEITMMAPVESKFSQSTGDID

KVTDTKPRVAEWRYGPARLWYDMLGVPEDGSGFDYGFKLRKTEHEPVIKSRMIEEFRKLEENNGTDLLAD

ENFLMVTQLHWEDDIIWDGEDVKHKGTKPQRASLAGWLPSSMTRNAMAYNVQQGFAATLDDDKPWYSIFP

IDNEDLVYGRWEDNIIWDAQAMPRLLEPPVLTLDPNDENLILEIPDEKEEATSNSPSKESKKESSLKKSR

ILLGKTGVIKEEPQQNMSQPEVKDPWNLSNDEYYYPKQQGLRGTFGGNIIQHSIPAVELRQPFFPTHMGP

IKLRQFHRPPLKKYSFGALSQPGPHSVQPLLKHIKKKAKMREQERQASGGGEMFFMRTPQDLTGKDGDLI

LAEYSEENGPLMMQVGMATKIKNYYKRKPGKDPGAPDCKYGETVYCHTSPFLGSLHPGQLLQAFENNLFR

APIYLHKMPETDFLIIRTRQGYYIRELVDIFVVGQQCPLFEVPGPNSKRANTHIRDFLQVFIYRLFWKSK

DRPRRIRMEDIKKAFPSHSESSIRKRLKLCADFKRTGMDSNWWVLKSDFRLPTEEEIRAMVSPEQCCAYY

SMIAAEQRLKDAGYGEKSFFAPEEENEEDFQMKIDDEVRTAPWNTTRAFIAAMKGKCLLEVTGVADPTGC

GEGFSYVKIPNKPTQQKDDKEPQPVKKTVTGTDADLRRLSLKNAKQLLRKFGVPEEEIKKLSRWEVIDVV

RTMSTEQARSGEGPMSKFARGSRFSVAEHQERYKEECQRIFDLQNKVLSSTEVLSTDTDSSSAEDSDFEE

MGKNIENMLQNKKTSSQLSREREEQERKELQRMLLAAGSAASGNNHRDDDTASVTSLNSSATGRCLKIYR

TFRDEEGKEYVRCETVRKPAVIDAYVRIRTTKDEEFIRKFALFDEQHREEMRKERRRIQEQLRRLKRNQE

KEKLKGPPEKKPKKMKERPDLKLKCGACGAIGHMRTNKFCPLYYQTNAPPSNPVAMTEEQEEELEKTVIH

NDNEELIKVEGTKIVLGKQLIESADEVRRKSLVLKFPKQQLPPKKKRRVGTTVHCDYLNRPHKSIHRRRT

DPMVTLSSILESIINDMRDLPNTYPFHTPVNAKVVKDYYKIITRPMDLQTLRENVRKRLYPSREEFREHL

ELIVKNSATYNGPKHSLTQISQSMLDLCDEKLKEKEDKLARLEKAINPLLDDDDQVAFSFILDNIVTQKM

MAVPDSWPFHHPVNKKFVPDYYKVIVNPMDLETIRKNISKHKYQSRESFLDDVNLILANSVKYNGPESQY

TKTAQEIVNVCYQTLTEYDEHLTQLEKDICTAKEAALEEAELESLDPMTPGPYTPQPPDLYDTNTSLSMS

RDASVFQDESNMSVLDIPSATPEKQEGEDGDGDLADEEEGTVQQPQASVLYEDLLMSEGEDDEEDAGSDE

EGDNPFSAIQLSESGSDSDVGSGGIRPKQPRMLQENTRMDMENEESMMSYEGDGGEASHGLEDSNISYGS

YEEPDPKSNTQDTSFSSIGNFRPGVVAHTCNLSTLGGRGGQIT

>HP1BP3

MATDTSQGELVHPKALPLIVGAQLIHADKLGEKVEDSTMPIRRTVNSTRETPPKSKLAEGEEEKPEPDIS

SEESVSTVEEQENETPPATSSEAEQPKGEPENEEKEENKSSEETKKERADSIHSTLFIIGQNS

>CHD1

MNGHSDEESVRNSSGESSQSDDDSGSASGSGSGSSSGSSSDGSSSQSGSSDSDSGSESGSQSESESDTSR

ENKVQAKPPKVDGAEFWKSSPSILAVQRSAILKKQQQQQQQQQHQASSNSGSEEDSSSSEDSDDSSSEVK

RKKHKDEDWQMSGSGSPSQSGSDSESEEEREKSSCDETESDYEPKNKVKSRKPQNRSKSKNGKKILGQKK

RQIDSSEEDDDEEDYDNDKRSSRRQATVNVSYKEDEEMKTDSDDLLEVCGEDVPQPEEEEFETIERFMDC

RIGRKGATGATTTIYAVEADGDPNAGFEKNKEPGEIQYLIKWKGWSHIHNTWETEETLKQQNVRGMKKLD

NYKKKDQETKRWLKNASPEDVEYYNCQQELTDDLHKQYQIVGRIIAHSNQKSAAGYPDYYCKWQGLPYSE

CSWEDGALISKKFQACIDEYFSRNQSKTTPFKDCKVLKQRPRFVALKKQPSYIGGHEGLELRDYQLNGLN

WLAHSWCKGNSCILADEMGLGKTIQTISFLNYLFHEHQLYGPFLLVVPLSTLTSWQREIQTWASQMNAVV

YLGDINSRNMIRTHEWTHHQTKRLKFNILLTTYEILLKDKAFLGGLNWAFIGVDEAHRLKNDDSLLYKTL

IDFKSNHRLLITGTPLQNSLKELWSLLHFIMPEKFSSWEDFEEEHGKGREYGYASLHKELEPFLLRRVKK

DVEKSLPAKVEQILRMEMSALQKQYYKWILTRNYKALSKGSKGSTSGFLNIMMELKKCCNHCYLIKPPDN

NEFYNKQEALQHLIRSSGKLILLDKLLIRLRERGNRVLIFSQMVRMLDILAEYLKYRQFPFQRLDGSIKG

ELRKQALDHFNAEGSEDFCFLLSTRAGGLGINLASADTVVIFDSDWNPQNDLQAQARAHRIGQKKQVNIY

RLVTKGSVEEDILERAKKKMVLDHLVIQRMDTTGKTVLHTGSAPSSSTPFNKEELSAILKFGAEELFKEP

EGEEQEPQEMDIDEILKRAETHENEPGPLTVGDELLSQFKVANFSNMDEDDIELEPERNSKNWEEIIPED

QRRRLEEEERQKELEEIYMLPRMRNCAKQISFNGSEGRRSRSRRYSGSDSDSISEGKRPKKRGRPRTIPR

ENIKGFSDAEIRRFIKSYKKFGGPLERLDAIARDAELVDKSETDLRRLGELVHNGCIKALKDSSSGTERT

GGRLGKVKGPTFRISGVQVNAKLVISHEEELIPLHKSIPSDPEERKQYTIPCHTKAAHFDIDWGKEDDSN

LLIGIYEYGYGSWEMIKMDPDLSLTHKILPDDPDKKPQAKQLQTRADYLIKLLSRDLAKKEALSGAGSSK

RRKARAKKNKAMKSIKVKEEIKSDSSPLPSEKSDEDDDKLSESKSDGRERSKKSSVSDAPVHITASGEPV

PISEESEELDQKTFSICKERMRPVKAALKQLDRPEKGLSEREQLEHTRQCLIKIGDHITECLKEYTNPEQ

IKQWRKNLWIFVSKFTEFDARKLHKLYKHAIKKRQESQQNSDQNSNLNPHVIRNPDVERLKENTNHDDSS

RDSYSSDRHLTQYHDHHKDRHQGDSYKKSDSRKRPYSSFSNGKDHRDWDHYKQDSRYYSDREKHRKLDDH

RSRDHRSNLEGSLKDRSHSDHRSHSDHRLHSDHRSSSEYTHHKSSRDYRYHSDWQMDHRASSSGPRSPLD

QRSYGSRSPFEHSVEHKSTPEHTWSSRKT

>polycomb group RING finger protein 6

MEGVAVVTAGSVGAAKTEGAAALPPPPPVSPPALTPAPAAGEEGPAPLSETGAPGCSGSRPPELEPERSL

GRFRGRFEDEDEELEEEEELEEEEEEEEEDMSHFSLRLEGGRQDSEDEEERLINLSELTPYILCSICKGY

LIDATTITECLHTFCKSCIVRHFYYSNRCPKCNIVVHQTQPLYNIRLDRQLQDIVYKLVINLEEREKKQM

HDFYKERGLEVPKPAVPQPVPSSKGRSKKVLESVFRIPPELDMSLLLEFIGANEGTGHFKPLEKKFVRVS

GEATIGHVEKFLRRKMGLDPACQVDIICGDHLLEQYQTLREIRRAIGDAAMQDGLLVLHYGLVVSPLKIT

>BPTF1

MRGRRGRPPKQPAAPAAERCAPAPPPPPPPPTSGPIGGLRSRHRGSSRGRWAAAQAEVAPKTRLSSPRGG

SSSRRKPPPPPPAPPSTSAPGRGGRGGGGGRTGGGGGGGHLARTTAARRAVNKVVYDDHESEEEEEEEDM

VSEEEEEEDGDAEETQDSEDDEEDEMEEDDDDSDYPEEMEDDDDDASYCTESSFRSHSTYSSTPGRRKPR

VHRPRSPILEEKDIPPLEFPKSSEDLMVPNEHIMNVIAIYEVLRNFGTVLRLSPFRFEDFCAALVSQEQC

TLMAEMHVVLLKAVLREEDTSNTTFGPADLKDSVNSTLYFIDGMTWPEVLRVYCESDKEYHHVLPYQEAE

DYPYGPVENKIKVLQFLVDQFLTTNIAREELMSEGVIQYDDHCRVCHKLGDLLCCETCSAVYHLECVKPP

LEEVPEDEWQCEVCVAHKVPGVTDCVAEIQKNKPYIRHEPIGYDRSRRKYWFLNRRLIIEEDTENENEKK

IWYYSTKVQLAELIDCLDKDYWEAELCKILEEMREEIHRHMDITEDLTNKARGSNKSFLAAANEEILESI

RAKKGDIDNVKSPEETEKDKNETENDSKDAEKNREEFEDQSLEKDSDDKTPDDDPEQGKSEVGDFKSEKS

NGELSESPGAGKGASGSTRIITRLRNPDSKLSQLKSQQVAAAAHEANKLFKEGKEVLVVNSQGEISRLST

KKEVIMKGNINNYFKLGQEGKYRVYHNQYSTNSFALNKHQHREDHDKRRHLAHKFCLTPAGEFKWNGSVH

GSKVLTISTLRLTITQLENNIPSSFLHPNWASHRANWIKAVQMCSKPREFALALAILECAVKPVVMLPIW

RESLGHTRLHRMTSIEREEKEKVKKKEKKQEEEETMQQATWVKYTFPVKHQVWKQKGEEYRVTGYGGWSW

ISKTHVYRFVPKLPGNTNVNYRKSLEGTKNNMDENMDESDKRKCSRSPKKIKIEPDSEKDEVKGSDAAKG

ADQNEMDISKITEKKDQDVKELLDSDSDKPCKEEPMEVDDDMKTESHVNCQESSQVDVVNVSEGFHLRTS

YKKKTKSSKLDGLLERRIKQFTLEEKQRLEKIKLEGGIKGIGKTSTNSSKNLSESPVITKAKEGCQSDSM

RQEQSPNANNDQPEDLIQGCSESDSSVLRMSDPSHTTNKLYPKDRVLDDVSIRSPETKCPKQNSIENDIE

EKVSDLASRGQEPSKSKTKGNDFFIDDSKLASADDIGTLICKNKKPLIQEESDTIVSSSKSALHSSVPKS

TNDRDATPLSRAMDFEGKLGCDSESNSTLENSSDTVSIQDSSEEDMIVQNSNESISEQFRTREQDVEVLE

PLKCELVSGESTGNCEDRLPVKGTEANGKKPSQQKKLEERPVNKCSDQIKLKNTTDKKNNENRESEKKGQ

RTSTFQINGKDNKPKIYLKGECLKEISESRVVSGNVEPKVNNINKIIPENDIKSLTVKESAIRPFINGDV

IMEDFNERNSSETKSHLLSSSDAEGNYRDSLETLPSTKESDSTQTTTPSASCPESNSVNQVEDMEIETSE

VKKVTSSPITSEEESNLSNDFIDENGLPINKNENVNGESKRKTVITEVTTMTSTVATESKTVIKVEKGDK

QTVVSSTENCAKSTVTTTTTTVTKLSTPSTGGSVDIISVKEQSKTVVTTTVTDSLTTTGGTLVTSMTVSK

EYSTRDKVKLMKFSRPKKTRSGTALPSYRKFVTKSSKKSIFVLPNDDLKKLARKGGIREVPYFNYNAKPA

LDIWPYPSPRPTFGITWRYRLQTVKSLAGVSLMLRLLWASLRWDDMAAKAPPGGGTTRTETSETEITTTE

IIKRRDVGPYGIRSEYCIRKIICPIGVPETPKETPTPQRKGLRSSALRPKRPETPKQTGPVIIETWVAEE

ELELWEIRAFAERVEKEKAQAVEQQAKKRLEQQKPTVIATSTTSPTSSTTSTISPAQKVMVAPISGSVTT

GTKMVLTTKVGSPATVTFQQNKNFHQTFATWVKQGQSNSGVVQVQQKVLGIIPSSTGTSQQTFTSFQPRT

ATVTIRPNTSGSGGTTSNSQVITGPQIRPGMTVIRTPLQQSTLGKAIIRTPVMVQPGAPQQVMTQIIRGQ

PVSTAVSAPNTVSSTPGQKSLTSATSTSNIQSSASQPPRPQQGQVKLTMAQLTQLTQGHGGNQGLTVVIQ

GQGQTTGQLQLIPQGVTVLPGPGQQLMQAAMPNGTVQRFLFTPLATTATTASTTTTTVSTTAAGTGEQRQ

SKLSPQMQVHQDKTLPPAQSSSVGPAEAQPQTAQPSAQPQPQTQPQSPAQPEVQTQPEVQTQTTVSSHVP

SEAQPTHAQSSKPQVAAQSQPQSNVQGQSPVRVQSPSQTRIRPSTPSQLSPGQQSQVQTTTSQPIPIQPH

TSLQIPSQGQPQSQPQVQSSTQTLSSGQTLNQVTVSSPSRPQLQIQQPQPQVIAVPQLQQQVQVLSQIQS

QVVAQIQAQQSGVPQQIKLQLPIQIQQSSAVQTHQIQNVVTVQAASVQEQLQRVQQLRDQQQKKKQQQIE

IKREHTLQASNQSEIIQKQVVMKHNAVIEHLKQKKSMTPAEREENQRMIVCNQVMKYILDKIDKEEKQAA

KKRKREESVEQKRSKQNATKLSALLFKHKEQLRAEILKKRALLDKDLQIEVQEELKRDLKIKKEKDLMQL

AQATAVAAPCPPVTPAPPAPPAPPPSPPPPPAVQHTGLLSTPTLPAASQKRKREEEKDSSSKSKKKKMIS

TTSKETKKDTKLYCICKTPYDESKFYIGCDRCQNWYHGRCVGILQSEAELIDEYVCPQCQSTEDAMTVLT

PLTEKDYEGLKRVLRSLQAHKMAWPFLEPVDPNDAPDYYGVIKEPMDLATMEERVQRRYYEKLTEFVADM

TKIFDNCRYYNPSDSPFYQCAEVLESFFVQKLKGFKASRSHNNKLQSTAS

>ING4

MAAGMYLEHYLDSIENLPFELQRNFQLMRDLDQRTEDLKAEIDKLATEYMSSARSLSSEEKLALLKQIQE

AYGKCKEFGDDKVQLAMQTYEMVDKHIRRLDTDLARFEADLKEKQIESSDYDSSSSKGRTQKEKKAARAR

SKGKNSDEEAPKTAQKKLKLVRTSPEYGMPSVTFGSVHPSDVLDMPVDPNEPTYCLCHQVSYGEMIGCDN

PDCSIEWFHFACVGLTTKPRGKWFCPRCSQERKKK

>ING1

MEILKELDECYERFSRETDGAQKRRMLHCVQRALIRSQELGDEKIQIVSQMVELVENRTRQVDSHVELFE

AQQELGDTVGNSGKVGADRPNGDAVAQSDKPNSKRSRRQRNNENRENASSNHDHDDGASGTPKEKKAKTS

KKKKRSKAKAEREASPADLPIDPNEPTYCLCNQVSYGEMIGCDNDECPIEWFHFSCVGLNHKPKGKWYCP

KCRGENEKTMDKALEKSKKERAYNR

>RAG2

MSLQMVTVSNNIALIQPGFSLMNFDGQVFFFGQKGWPKRSCPTGVFHLDVKHNHVKLKPTIFSKDSCYLP

PLRYPATCTFKGSLESEKHQYIIHGGKTPNNEVSDKIYVMSIVCKNNKKVTFRCTEKDLVGDVPEARYGH

SINVVYSRGKSMGVLFGGRSYMPSTHRTTEKWNSVADCLPCVFLVDFEFGCATSYILPELQDGLSFHVSI

AKNDTIYILGGHSLANNIRPANLYRIRVDLPLGSPAVNCTVLPGGISVSSAILTQTNNDEFVIVGGYQLE

NQKRMICNIISLEDNKIEIREMETPDWTPDIKHSKIWFGSNMGNGTVFLGIPGDNKQVVSEGFYFYMLKC

AEDDTNEEQTTFTNSQTSTEDPGDSTPFEDSEEFCFSAEANSFDGDDEFDTYNEDDEEDESETGYWITCC

PTCDVDINTWVPFYSTELNKPAMIYCSHGDGHWVHAQCMDLAERTLIHLSAGSNKYYCNEHVEIARALHT

PQRVLPLKKPPMKSLRKKGSGKILTPAKKSFLRRLFD

>WDR5

MATEEKKPETEAARAQPTPSSSATQSKPTPVKPNYALKFTLAGHTKAVSSVKFSPNGEWLASSSADKLIK

IWGAYDGKFEKTISGHKLGISDVAWSSDSNLLVSASDDKTLKIWDVSSGKCLKTLKGHSNYVFCCNFNPQ

SNLIVSGSFDESVRIWDVKTGKCLKTLPAHSDPVSVHFNRDGSLIVSSSYDGLCRIWDTASGQCLKTLID

DDNPPVSFVKFSPNGKYILAATLDNTLKLWDYSKGKCLKTYTGHKNEKYCIFANFSVTGGKWIVSGSEDN

LVYIWNLQTKEIVQKLQGHTDVVISTACHPTENIIASAALENDKTIKLWKSDC

>EED

MSEREVSTAPAGTDMPAAKKQKLSSDENSNPDLSGDENDDAVSIESGTNTERPDTPTNTPNAPGRKSWGK

GKWKSKKCKYSFKCVNSLKEDHNQPLFGVQFNWHSKEGDPLVFATVGSNRVTLYECHSQGEIRLLQSYVD

ADADENFYTCAWTYDSNTSHPLLAVAGSRGIIRIINPITMQCIKHYVGHGNAINELKFHPRDPNLLLSVS

KDHALRLWNIQTDTLVAIFGGVEGHRDEVLSADYDLLGEKIMSCGMDHSLKLWRINSKRMMNAIKESYDY

NPNKTNRPFISQKIHFPDFSTRDIHRNYVDCVRWLGDLILSKSGRAILHSHQQCMRDPVSPNLRQHLSCE

NAIVCWKPGKMEDDIDKIKPSESNVTILGRFDYSQCDIWYMRFSMDFWQKMLALGNQVGKLYVWDLEVED

PHKAKCTTLTHHKCGAAIRQTSFSRDSSILIAVCDDASIWRWDRLR

>DCAF1

MTTVVVHVDSKAELTTLLEQWEKEHGSGQDMVPILTRMSQLIEKETEEYRKGDPDPFDDRHPGRADPECM

LGHLLRILFKNDDFMNALVNAYVMTSREPPLNTAACRLLLDIMPGLETAVVFQEKEGIVENLFKWAREAD

QPLRTYSTGLLGGAMENQDIAANYRDENSQLVAIVLRRLRELQLQEVALRQENKRPSPRKLSSEPLLPLD

EEAVDMDYGDMAVDVVDGDQEEASGDMEISFHLDSGHKTSSRVNSTTKPEDGGLKKNKSAKQGDRENFRK

AKQKLGFSSSDPDRMFVELSNSSWSEMSPWVIGTNYTLYPMTPAIEQRLILQYLTPLGEYQELLPIFMQL

GSRELMMFYIDLKQTNDVLLTFEALKHLASLLLHNKFATEFVAHGGVQKLLEIPRPSMAATGVSMCLYYL

SYNQDAMERVCMHPHNVLSDVVNYTLWLMECSHASGCCHATMFFSICFSFRAVLELFDRYDGLRRLVNLI

STLEILNLEDQGALLSDDEIFASRQTGKHTCMALRKYFEAHLAIKLEQVKQSLQRTEGGILVHPQPPYKA

CSYTHEQIVEMMEFLIEYGPAQLYWEPAEVFLKLSCVQLLLQLISIACNWKTYYARNDTVRFALDVLAIL

TVVPKIQLQLAESVDVLDEAGSTVSTVGISIILGVAEGEFFIHDAEIQKSALQIIINCVCGPDNRISSIG

KFISGTPRRKLPQNPKSSEHTLAKMWNVVQSNNGIKVLLSLLSIKMPITDADQIRALACKALVGLSRSST

VRQIISKLPLFSSCQIQQLMKEPVLQDKRSDHVKFCKYAAELIERVSGKPLLIGTDVSLARLQKADVVAQ

SRISFPEKELLLLIRNHLISKGLGETATVLTKEADLPMTAASHSSAFTPVTAAASPVSLPRTPRIANGIA

TRLGSHAAVGASAPSAPTAHPQPRPPQGPLALPGPSYAGNSPLIGRISFIRERPSPCNGRKIRVLRQKSD

HGAYSQSPAIKKQLDRHLPSPPTLDSIITEYLREQHARCKNPVATCPPFSLFTPHQCPEPKQRRQAPINF

TSRLNRRASFPKYGGVDGGCFDRHLIFSRFRPISVFREANEDESGFTCCAFSARERFLMLGTCTGQLKLY

NVFSGQEEASYNCHNSAITHLEPSRDGSLLLTSATWSQPLSALWGMKSVFDMKHSFTEDHYVEFSKHSQD

RVIGTKGDIAHIYDIQTGNKLLTLFNPDLANNYKRNCATFNPTDDLVLNDGVLWDVRSAQAIHKFDKFNM

NISGVFHPNGLEVIINTEIWDLRTFHLLHTVPALDQCRVVFNHTGTVMYGAMLQADDEDDLMEERMKSPF

GSSFRTFNATDYKPIATIDVKRNIFDLCTDTKDCYLAVIENQGSMDALNMDTVCRLYEVGRQRLAEDEDE

EEDQEEEEQEEEDDDEDDDDTDDLDELDTDQLLEAELEEDDNNENAGEDGDNDFSPSDEELANLLEEGED

GEDEDSDADEEVELILGDTDSSDNSDLEDDIILSLNE

>SMN

MAMSSGGSGGGVPEQEDSVLFRRGTGQSDDSDIWDDTALIKAYDKAVASFKHALKNGDICETSGKPKTTP

KRKPAKKNKSQKKNTAASLQQWKVGDKCSAIWSEDGCIYPATIASIDFKRETCVVVYTGYGNREEQNLSD

LLSPICEVANNIEQNAQENENESQVSTDESENSRSPGNKSDNIKPKSAPWNSFLPPPPPMPGPRLGPGKP

GLKFNGPPPPPPPPPPHLLSCWLPPFPSGPPIIPPPPPICPDSLDDADALGSMLISWYMSGYHTGYYMGF

RQNQKEGRCSHSLN

>53BP1

MDPTGSQLDSDFSQQDTPCLIIEDSQPESQVLEDDSGSHFSMLSRHLPNLQTHKENPVLDVVSNPEQTAG

EERGDGNSGFNEHLKENKVADPVDSSNLDTCGSISQVIEQLPQPNRTSSVLGMSVESAPAVEEEKGEELE

QKEKEKEEDTSGNTTHSLGAEDTASSQLGFGVLELSQSQDVEENTVPYEVDKEQLQSVTTNSGYTRLSDV

DANTAIKHEEQSNEDIPIAEQSSKDIPVTAQPSKDVHVVKEQNPPPARSEDMPFSPKASVAAMEAKEQLS

AQELMESGLQIQKSPEPEVLSTQEDLFDQSNKTVSSDGCSTPSREEGGCSLASTPATTLHLLQLSGQRSL

VQDSLSTNSSDLVAPSPDAFRSTPFIVPSSPTEQEGRQDKPMDTSVLSEEGGEPFQKKLQSGEPVELENP

PLLPESTVSPQASTPISQSTPVFPPGSLPIPSQPQFSHDIFIPSPSLEEQSNDGKKDGDMHSSSLTVECS

KTSEIEPKNSPEDLGLSLTGDSCKLMLSTSEYSQSPKMESLSSHRIDEDGENTQIEDTEPMSPVLNSKFV

PAENDSILMNPAQDGEVQLSQNDDKTKGDDTDTRDDISILATGCKGREETVAEDVCIDLTCDSGSQAVPS

PATRSEALSSVLDQEEAMEIKEHHPEEGSSGSEVEEIPETPCESQGEELKEENMESVPLHLSLTETQSQG

LCLQKEMPKKECSEAMEVETSVISIDSPQKLAILDQELEHKEQEAWEEATSEDSSVVIVDVKEPSPRVDV

SCEPLEGVEKCSDSQSWEDIAPEIEPCAENRLDTKEEKSVEYEGDLKSGTAETEPVEQDSSQPSLPLVRA

DDPLRLDQELQQPQTQEKTSNSLTEDSKMANAKQLSSDAEAQKLGKPSAHASQSFCESSSETPFHFTLPK

EGDIIPPLTGATPPLIGHLKLEPKRHSTPIGISNYPESTIATSDVMSESMVETHDPILGSGKGDSGAAPD

VDDKLCLRMKLVSPETEASEESLQFNLEKPATGERKNGSTAVAESVASPQKTMSVLSCICEARQENEARS

EDPPTTPIRGNLLHFPSSQGEEEKEKLEGDHTIRQSQQPMKPISPVKDPVSPASQKMVIQGPSSPQGEAM

VTDVLEDQKEGRSTNKENPSKALIERPSQNNIGIQTMECSLRVPETVSAATQTIKNVCEQGTSTVDQNFG

KQDATVQTERGSGEKPVSAPGDDTESLHSQGEEEFDMPQPPHGHVLHRHMRTIREVRTLVTRVITDVYYV

DGTEVERKVTEETEEPIVECQECETEVSPSQTGGSSGDLGDISSFSSKASSLHRTSSGTSLSAMHSSGSS

GKGAGPLRGKTSGTEPADFALPSSRGGPGKLSPRKGVSQTGTPVCEEDGDAGLGIRQGGKAPVTPRGRGR

RGRPPSRTTGTRETAVPGPLGIEDISPNLSPDDKSFSRVVPRVPDSTRRTDVGAGALRRSDSPEIPFQAA

AGPSDGLDASSPGNSFVGLRVVAKWSSNGYFYSGKITRDVGAGKYKLLFDDGYECDVLGKDILLCDPIPL

DTEVTALSEDEYFSAGVVKGHRKESGELYYSIEKEGQRKWYKRMAVILSLEQGNRLREQYGLGPYEAVTP

LTKAADISLDNLVEGKRKRRSNVSSPATPTASSSSSTTPTRKITESPRASMGVLSGKRKLITSEEERSPA

KRGRKSATVKPGAVGAGEFVSPCESGDNTGEPSALEEQRGPLPLNKTLFLGYAFLLTMATTSDKLASRSK

LPDGPTGSSEEEEEFLEIPPFNKQYTESQLRAGAGYILEDFNEAQCNTAYQCLLIADQHCRTRKYFLCLA

SGIPCVSHVWVHDSCHANQLQNYRNYLLPAGYSLEEQRILDWQPRENPFQNLKVLLVSDQQQNFLELWSE

ILMTGGAASVKQHHSSAHNKDIALGVFDVVVTDPSCPASVLKCAEALQLPVVSQEWVIQCLIVGERIGFK

QHPKYKHDYVSH

>SND1

MASSAQSGGSSGGPAVPTVQRGIIKMVLSGCAIIVRGQPRGGPPPERQINLSNIRAGNLARRAAATQPDA

KDTPDEPWAFPAREFLRKKLIGKEVCFTIENKTPQGREYGMIYLGKDTNGENIAESLVAEGLATRREGMR

ANNPEQNRLSECEEQAKAAKKGMWSEGNGSHTIRDLKYTIENPRHFVDSHHQKPVNAIIEHVRDGSVVRA

LLLPDYYLVTVMLSGIKCPTFRREADGSETPEPFAAEAKFFTESRLLQRDVQIILESCHNQNILGTILHP

NGNITELLLKEGFARCVDWSIAVYTRGAEKLRAAERFAKERRLRIWRDYVAPTANLDQKDKQFVAKVMQV

LNADAIVVKLNSGDYKTIHLSSIRPPRLEGENTQDKNKKLRPLYDIPYMFEAREFLRKKLIGKKVNVTVD

YIRPASPATETVPAFSERTCATVTIGGINIAEALVSKGLATVIRYRQDDDQRSSHYDELLAAEARAIKNG

KGLHSKKEVPIHRVADISGDTQKAKQFLPFLQRAGRSEAVVEYVFSGSRLKLYLPKETCLITFLLAGIEC

PRGARNLPGLVQEGEPFSEEATLFTKELVLQREVEVEVESMDKAGNFIGWLHIDGANLSVLLVEHALSKV

HFTAERSSYYKSLLSAEEAAKQKKEKVWAHYEEQPVEEVMPVLEEKERSASYKPVFVTEITDDLHFYVQD

VETGTQLEKLMENMRNDIASHPPVEGSYAPRRGEFCIAKFVDGEWYRARVEKVESPAKIHVFYIDYGNRE

VLPSTRLGTLSPAFSTRVLPAQATEYAFAFIQVPQDDDARTDAVDSVVRDIQNTQCLLNVEHLSAGCPHV

TLQFADSKGDVGLGLVKEGLVMVEVRKEKQFQKVITEYLNAQESAKSARLNLWRYGDFRADDADEFGYSR

>L3MBTL1

MRRREGHGTDSEMGQGPVRESQSSDPPALQFRISEYKPLNMAGVEQPPSPELRQEGVTEYEDGGAPAGDG

EAGPQQAEDHPQNPPEDPNQDPPEDDSTCQCQACGPHQAAGPDLGSSNDGCPQLFQERSVIVENSSGSTS

ASELLKPMKKRKRREYQSPSEEESEPEAMEKQEEGKDPEGQPTASTPESEEWSSSQPATGEKKECWSWES

YLEEQKAITAPVSLFQDSQAVTHNKNGFKLGMKLEGIDPQHPSMYFILTVAEVCGYRLRLHFDGYSECHD

FWVNANSPDIHPAGWFEKTGHKLQPPKGYKEEEFSWSQYLRSTRAQAAPKHLFVSQSHSPPPLGFQVGMK

LEAVDRMNPSLVCVASVTDVVDSRFLVHFDNWDDTYDYWCDPSSPYIHPVGWCQKQGKPLTPPQDYPDPD

NFCWEKYLEETGASAVPTWAFKVRPPHSFLVNMKLEAVDRRNPALIRVASVEDVEDHRIKIHFDGWSHGY

DFWIDADHPDIHPAGWCSKTGHPLQPPLGPREPSSASPGGCPPLSYRSLPHTRTSKYSFHHRLFFPRKCP

TPGCDGSGHVTGKFTAHHCLSGCPLAERNQSRLKAELSDSEASARKKNLSGFSPRKKPRHHGRIGRPPKY

RKIPQEDFQTLTPDVVHQSLFMSALSAHPDRSLSVCWEQHCKLLPGVAGISASTVAKWTIDEVFGFVQTL

TGCEDQARLFKDEVRCKCRVGDRAGVTVLKTAGSRCPPQRHFC

>SFMBT1

MNGEQQLDADAGSGMEEVELSWEDYLEETGSTAVPYGSFKHVDTRLQNGFAPGMKLEVAVRTDPETYWVA

TVITTCEQLLLLRYDGYGEDRRADFWCDIRKADLYPIGWCEQNKKTLEAPEGIRDKVSDWDEFLRQTLIG

ACSPPVPLLEGLRNGRNPLDLIAPGSRLECQAFQDSLSTWIVTVVENIGGRLKLRYEGLESSDNYEHWLY

YLDPFLHHVGWAAQQGYELQPPSAIRHLKNEAEWQEILAKVKEEEEEPLPSYLFKDKQVIGIHTFSVNMK

LEAVDPWSPFGISPATVVKVFDEKYFLVEMDDLRPENHARRSFVCHADSPGIFPVQWSLKNGLHISPPPG

YPSQDFDWADYLKQCGAEAAPQRCFPPLISEHEFKENMKLEAVNPILPEEVCVATITAVRGSYLWLQLEG

SKKPIPECIVSVESMDIFPLGWCETNGHPLSTPRRARVYKQRKIAVVQPEKQVPSSRTVHEGLRNQELNS

TESVMINGKYCCPKIYFNHRCFSGPYLNKGRIAELPQCVGPGNCVLVLREVLTLLINAAYKPSRVLRELQ

LDKDSVWHGCGEVLKAKYKGKSYRATVEIVKTADRVTEFCRQTCIKLECCPNLFGPRMVLDKCSENCSVL

TKTKYTHYYGKKKNKRIGRPPGGHSNLACALKKASKRRKRRKNVFVHKKKRSSASVDNTPAGSPQGSGGE

DEDDPDEGDDDSLSEGSTSEQQDELQEESEMSEKKSCSSSPTQSEISTSLPPDRQRRKRELRTFSFSDDE

NKPPSPKEIRIEVAERLHLDSNPLKWSVADVVRFIRSTDCAPLARIFLDQEIDGQALLLLTLPTVQECMD

LKLGPAIKLCHHIERIKFAFYEQFAN

>LEDGF

MTRDFKPGDLIFAKMKGYPHWPARVDEVPDGAVKPPTNKLPIFFFGTHETAFLGPKDIFPYSENKEKYGK

PNKRKGFNEGLWEIDNNPKVKFSSQQAATKQSNASSDVEVEEKETSVSKEDTDHEEKASNEDVTKAVDIT

TPKAARRGRKRKAGVVTTATASVNLKVSPKRGRPAATEVKIPKPRGRPKMVKQPCPSESDIITEEDKSKK

KGQEEKQPKKQPKKDEEGQKEEDKPRKEPDKKEGKKEVESKRKNLAKTGVTSTSDSEEEGDDQEGEKKRK

GGRNFQTAHRRNMLKGQHEKEAADRKRKQEEQMETEHQTTCNLQ

>NSD2

MEFSIKQSPLSVQSVVKCIKMKQAPEILGSANGKTPSCEVNRECSVFLSKAQLSSSLQEGVMQKFNGHDA

LPFIPADKLKDLTSRVFNGEPGAHDAKLRFESQEMKGIGTPPNTTPIKNGSPEIKLKITKTYMNGKPLFE

SSICGDSAADVSQSEENGQKPENKARRNRKRSIKYDSLLEQGLVEAALVSKISSPSDKKIPAKKESCPNT

GRDKDHLLKYNVGDLVWSKVSGYPWWPCMVSADPLLHSYTKLKGQKKSARQYHVQFFGDAPERAWIFEKS

LVAFEGEGQFEKLCQESAKQAPTKAEKIKLLKPISGKLRAQWEMGIVQAEEAASMSVEERKAKFTFLYVG

DQLHLNPQVAKEAGIAAESLGEMAESSGVSEEAAENPKSVREECIPMKRRRRAKLCSSAETLESHPDIGK

STPQKTAEADPRRGVGSPPGRKKTTVSMPRSRKGDAASQFLVFCQKHRDEVVAEHPDASGEEIEELLRSQ

WSLLSEKQRARYNTKFALVAPVQAEEDSGNVNGKKRNHTKRIQDPTEDAEAEDTPRKRLRTDKHSLRKRD

TITDKTARTSSYKAMEAASSLKSQAATKNLSDACKPLKKRNRASTAASSALGFSKSSSPSASLTENEVSD

SPGDEPSESPYESADETQTEVSVSSKKSERGVTAKKEYVCQLCEKPGSLLLCEGPCCGAFHLACLGLSRR

PEGRFTCSECASGIHSCFVCKESKTDVKRCVVTQCGKFYHEACVKKYPLTVFESRGFRCPLHSCVSCHAS

NPSNPRPSKGKMMRCVRCPVAYHSGDACLAAGCSVIASNSIICTAHFTARKGKRHHAHVNVSWCFVCSKG

GSLLCCESCPAAFHPDCLNIEMPDGSWFCNDCRAGKKLHFQDIIWVKLGNYRWWPAEVCHPKNVPPNIQK

MKHEIGEFPVFFFGSKDYYWTHQARVFPYMEGDRGSRYQGVRGIGRVFKNALQEAEARFREIKLQREARE

TQESERKPPPYKHIKVNKPYGKVQIYTADISEIPKCNCKPTDENPCGFDSECLNRMLMFECHPQVCPAGE

FCQNQCFTKRQYPETKIIKTDGKGWGLVAKRDIRKGEFVNEYVGELIDEEECMARIKHAHENDITHFYML

TIDKDRIIDAGPKGNYSRFMNHSCQPNCETLKWTVNGDTRVGLFAVCDIPAGTELTFNYNLDCLGNEKTV

CRCGASNCSGFLGDRPKTSTTLSSEEKGKKTKKKTRRRRAKGEGKRQSEDECFRCGDGGQLVLCDRKFCT

KAYHLSCLGLGKRPFGKWECPWHHCDVCGKPSTSFCHLCPNSFCKEHQDGTAFSCTPDGRSYCCEHDLGA

ASVRSTKTEKPPPEPGKPKGKRRRRRGWRRVTEGK

>AF9

MAVKVQFEIGHTSKLRSKKTPHPQAFTHDWEIYVQGVNKADISAFVEKVVFVLHESFPKPKRVVKEPPYA

IQESGYAGFLLPVEIYFRNRDEPKRIVYQYDLVLQSTGPPQHHVEVKTHIFEAPSEEFRTKLMRGGGVPV

FGANIGAGSLARTLSPSVGSGETAHSNEMSGVKAKGVPGTISMNSDLNTGKKHKSRSEDPGKSNAFSALF

GPPITKTSVTANNMAQVPVSKHSPEAKSAAVGGKGVFHEGREKASKDRDKSSGGDKPREKDKKDRHGRDK

DRKSSKSGDGKHERDKEKSKKDKSRDKEREREQGSGNTTSANALTVATSVGTAGGLVKHTASPKPGKPNE

MGAPSPKKTANDSAAGAVAPTSRSKERDEHKSVKSESKDKERSSSKKSKKDKKDKDKQRDESKDKRMPKD

ERPGQSDSPASGNLQSAHLQQQSQVASTGKATPTSVGSNSSSSMVSNIGKQKEEATGKHSEKAAESKGDK

STSNGNATDTKKSHKHKKKEKSKDKEKERNDRDKDKDKERDKDKLKEREKDKQHVTGSEKSSTVDPSPKL

EGASPATEGGAPDKTPVSASASSGSKKEKHKKSKEKSSGKDEKRQREAVDKQADTKHTGKSQQSQSGQSK

TPSNLDLSDMDSAQSAPDLAATNALAAAAAATLQHSDSSNSSFPDLPARSSSQQKPTKGNTGSGKETKST

VKEHKGKSKQNDKTLEKNTDKTEKPDKTPDPKVDKSEKSEKEDKKRNRRSSQSSNSGSGAGVTGAFIEPP

VKASKKEQGKAAGEKSKSPSLRPPRETNSTGTGGGTPNAGAGFLAPPSSSPSNNHSAAVAASLNNNNNNN

NNNTNSNSSHSPGEMTTPGQLQLPTEYLSELQELHHKIMTLQDNEELQHVVEMIAATGCYEITHKTFDFD

LCKLDRGTVQRLQEFLATSVS

>YEATS2

MSGIKRTIKETDPDYEDVSVALPNKRHKAIENSARDAAVQKIETIIKEQFALEMKNKEHEIEVIDQRLIE

ARRMMDKLRACIVANYYASAGLLKVSEGSKTCDTMVFNHPAIKKFLESPSRSSSPANQRAETPSANHSES

DSLSQHNDFLSDKDNNSNMDIEERLSNNMEQRPSRNTGRDTSRITGSHKTEQRNADLTDETSRLFVKKTI

VVGNVSKYIPPDKREENDQSTHKWMVYVRGSRREPSINHFVKKVWFFLHPSYKPNDLVEVREPPFHLTRR

GWGEFPVRVQVHFKDSQNKRIDIIHNLKLDRTYTGLQTLGAETVVDVELHRHSLGEDCIYPQSSESDISD

APPSLPLTIPAPVKASSPIKQSHEPVPDTSVEKGFPASTEAERHTPFYALPSSLERTPTKMTTSQKVTFC

SHGNSAFQPIASSCKIVPQSQVPNPESPGKSFQPITMSCKIVSGSPISTPSPSPLPRTPTSTPVHVKQGT

AGSVINNPYVIMDKQPGQVIGATTPSTGSPTNKISTASQVSQGTGSPVPKIHGSSFVTSTVKQEDSLFAS

MPPLCPIGSHPKVQSPKPITGGLGAFTKVIIKQEPGEAPHVPATGAASQSPLPQYVTVKGGHMIAVSPQK

QVITPGEGIAQSAKVQPSKVVGVPVGSALPSTVKQAVAISGGQILVAKASSSVSKAVGPKQVVTQGVAKA

IVSGGGGTIVAQPVQTLTKAQVTAAGPQKSGSQGSVMATLQLPATNLANLANLPPGTKLYLTTNSKNPSG

KGKLLLIPQGAILRATNNANLQSGSAASGGSGAGGGGGGGGGGGSGSGGGGSTGGGGGTAGGGTQSTAGP

GGISQHLTYTSYILKQTPQGTFLVGQPSPQTSGKQLTTGSVVQGTLGVSTSSAQGQQTLKVISGQKTTLF

TQAAHGGQASLMKISDSTLKTVPATSQLSKPGTTMLRVAGGVITTATSPAVALSANGPAQQSEGMAPVSS

STVSSVTKTSGQQQVCVSQATVGTCKAATPTVVSATSLVPTPNPISGKATVSGLLKIHSSQSSPQQAVLT

IPSQLKPLSVNTSGGVQTILMPVNKVVQSFSTSKPPAILPVAAPTPVVPSSAPAAVAKVKTEPETPGPSC

LSQEGQTAVKTEESSELGNYVIKIDHLETIQQLLTAVVKKIPLITAKSEDASCFSAKSVEQYYGWNIGKR

RAAEWQRAMTMRKVLQEILEKNPRFHHLTPLKTKHIAHWCRCHGYTPPDPESLRNDGDSIEDVLTQIDSE

PECPSSFSSADNLCRKLEDLQQFQKREPENEEEVDILSLSEPVKINIKKEQEEKQEEVKFYLPPTPGSEF

IGDVTQKIGITLQPVALHRNVYASVVEDMILKATEQLVNDILRQALAVGYQTASHNRIPKEITVSNIHQA

ICNIPFLDFLTNKHMGILNEDQ

>ATRX

MTAEPMSESKLNTLVQKLHDFLAHSSEESEETSSPPRLAMNQNTDKISGSGSNSDMMENSKEEGTSSSEK

SKSSGSSRSKRKPSIVTKYVESDDEKPLDDETVNEDASNENSENDITMQSLPKEDGLHGIVSCTACGQQV

NHFQKDSIYRHPSLQVLICKNCFKYYMSDDISRDSDGMDEQCRWCAEGGNLICCDFCHNAFCKKCILRNL

GRKELSTIMDENNQWYCYICHPEPLLDLVTACNSVFENLEQLLQQNKKKIKVDSEKSNKVYEHTSRFSPK

KTSSNCNGEEKKLDDSCSGSVTYSYSALIVPKEMIKKAKKLIETTANMNSSYVKFLKQATDNSEISSATK

LRQLKAFKSVLADIKKAHLALEEDLNSEFRAMDAVNKEKNTKEHKVIDAKFETKARKGEKPCALEKKDIS

KSEAKLSRKQVDSEHMHQNVPTEEQRTNKSTGGEHKKSDRKEEPQYEPANTSEDLDMDIVSVPSSVPEDI

FENLETAMEVQSSVDHQGDGSSGTEQEVESSSVKLNISSKDNRGGIKSKTTAKVTKELYVKLTPVSLSNS

PIKGADCQEVPQDKDGYKSCGLNPKLEKCGLGQENSDNEHLVENEVSLLLEESDLRRSPRVKTTPLRRPT

ETNPVTSNSDEECNETVKEKQKLSVPVRKKDKRNSSDSAIDNPKPNKLPKSKQSETVDQNSDSDEMLAIL

KEVSRMSHSSSSDTDINEIHTNHKTLYDLKTQAGKDDKGKRKRKSSTSGSDFDTKKGKSAKSSIISKKKR

QTQSESSNYDSELEKEIKSMSKIGAARTTKKRIPNTKDFDSSEDEKHSKKGMDNQGHKNLKTSQEGSSDD

AERKQERETFSSAEGTVDKDTTIMELRDRLPKKQQASASTDGVDKLSGKEESFTSLEVRKVAETKEKSKH

LKTKTCKKVQDGLSDIAEKFLKKDQSDETSEDDKKQSKKGTEEKKKPSDFKKKVIKMEQQYESSSDGTEK

LPEREEICHFPKGIKQIKNGTTDGEKKSKKIRDKTSKKKDELSDYAEKSTGKGDSCDSSEDKKSKNGAYG

REKKRCKLLGKSSRKRQDCSSSDTEKYSMKEDGCNSSDKRLKRIELRERRNLSSKRNTKEIQSGSSSSDA

EESSEDNKKKKQRTSSKKKAVIVKEKKRNSLRTSTKRKQADITSSSSSDIEDDDQNSIGEGSSDEQKIKP

VTENLVLSSHTGFCQSSGDEALSKSVPVTVDDDDDDNDPENRIAKKMLLEEIKANLSSDEDGSSDDEPEE

GKKRTGKQNEENPGDEEAKNQVNSESDSDSEESKKPRYRHRLLRHKLTVSDGESGEEKKTKPKEHKEVKG

RNRRKVSSEDSEDSDFQESGVSEEVSESEDEQRPRTRSAKKAELEENQRSYKQKKKRRRIKVQEDSSSEN

KSNSEEEEEEKEEEEEEEEEEEEEEEDENDDSKSPGKGRKKIRKILKDDKLRTETQNALKEEEERRKRIA

EREREREKLREVIEIEDASPTKCPITTKLVLDEDEETKEPLVQVHRNMVIKLKPHQVDGVQFMWDCCCES

VKKTKKSPGSGCILAHCMGLGKTLQVVSFLHTVLLCDKLDFSTALVVCPLNTALNWMNEFEKWQEGLKDD

EKLEVSELATVKRPQERSYMLQRWQEDGGVMIIGYEMYRNLAQGRNVKSRKLKEIFNKALVDPGPDFVVC

DEGHILKNEASAVSKAMNSIRSRRRIILTGTPLQNNLIEYHCMVNFIKENLLGSIKEFRNRFINPIQNGQ

CADSTMVDVRVMKKRAHILYEMLAGCVQRKDYTALTKFLPPKHEYVLAVRMTSIQCKLYQYYLDHLTGVG

NNSEGGRGKAGAKLFQDFQMLSRIWTHPWCLQLDYISKENKGYFDEDSMDEFIASDSDETSMSLSSDDYT

KKKKKGKKGKKDSSSSGSGSDNDVEVIKVWNSRSRGGGEGNVDETGNNPSVSLKLEESKATSSSNPSSPA

PDWYKDFVTDADAEVLEHSGKMVLLFEILRMAEEIGDKVLVFSQSLISLDLIEDFLELASREKTEDKDKP

LIYKGEGKWLRNIDYYRLDGSTTAQSRKKWAEEFNDETNVRGRLFIISTKAGSLGINLVAANRVIIFDAS

WNPSYDIQSIFRVYRFGQTKPVYVYRFLAQGTMEDKIYDRQVTKQSLSFRVVDQQQVERHFTMNELTELY

TFEPDLLDDPNSEKKKKRDTPMLPKDTILAELLQIHKEHIVGYHEHDSLLDHKEEEELTEEERKAAWAEY

EAEKKGLTMRFNIPTGTNLPPVSFNSQTPYIPFNLGALSAMSNQQLEDLINQGREKVVEATNSVTAVRIQ

PLEDIISAVWKENMNLSEAQVQALALSRQASQELDVKRREAIYNDVLTKQQMLISCVQRILMNRRLQQQY

NQQQQQQMTYQQATLGHLMMPKPPNLIMNPSNYQQIDMRGMYQPVAGGMQPPPLQRAPPPMRSKNPGPSQ

GKSM

>DNMT3L

MAAIPALDPEAEPSMDVILVGSSELSSSVSPGTGRDLIAYEVKANQRNIEDICICCGSLQVHTQHPLFEG

GICAPCKDKFLDALFLYDDDGYQSYCSICCSGETLLICGNPDCTRCYCFECVDSLVGPGTSGKVHAMSNW

VCYLCLPSSRSGLLQRRRKWRSQLKAFYDRESENPLEMFETVPVWRRQPVRVLSLFEDIKKELTSLGFLE

SGSDPGQLKHVVDVTDTVRKDVEEWGPFDLVYGATPPLGHTCDRPPSWYLFQFHRLLQYARPKPGSPRPF

FWMFVDNLVLNKEDLDVASRFLEMEPVTIPDVHGGSLQNAVRVWSNIPAIRSSRHWALVSEEELSLLAQN

KQSSKLAAKWPTKLVKNCFLPLREYFKYFSTELTSSL

>KAP1

MAASAAAASAAAASAASGSPGPGEGSAGGEKRSTAPSAAASASASAAASSPAGGGAEALELLEHCGVCRE

RLRPEREPRLLPCLHSACSACLGPAAPAAANSSGDGGAAGDGTVVDCPVCKQQCFSKDIVENYFMRDSGS

KAATDAQDANQCCTSCEDNAPATSYCVECSEPLCETCVEAHQRVKYTKDHTVRSTGPAKSRDGERTVYCN

VHKHEPLVLFCESCDTLTCRDCQLNAHKDHQYQFLEDAVRNQRKLLASLVKRLGDKHATLQKSTKEVRSS

IRQVSDVQKRVQVDVKMAILQIMKELNKRGRVLVNDAQKVTEGQQERLERQHWTMTKIQKHQEHILRFAS

WALESDNNTALLLSKKLIYFQLHRALKMIVDPVEPHGEMKFQWDLNAWTKSAEAFGKIVAERPGTNSTGP

APMAPPRAPGPLSKQGSGSSQPMEVQEGYGFGSGDDPYSSAEPHVSGVKRSRSGEGEVSGLMRKVPRVSL

ERLDLDLTADSQPPVFKVFPGSTTEDYNLIVIERGAAAAATGQPGTAPAGTPGAPPLAGMAIVKEEETEA

AIGAPPTATEGPETKPVLMALAEGPGAEGPRLASPSGSTSSGLEVVAPEGTSAPGGGPGTLDDSATICRV

CQKPGDLVMCNQCEFCFHLDCHLPALQDVPGEEWSCSLCHVLPDLKEEDGSLSLDGADSTGVVAKLSPAN

QRKCERVLLALFCHEPCRPLHQLATDSTFSLDQPGGTLDLTLIRARLQEKLSPPYSSPQEFAQDVGRMFK

QFNKLTEDKADVQSIIGLQRFFETRMNEAFGDTKFSAVLVEPPPMSLPGAGLSSQELSGGPGDGP

>ZMYND11

MSRVHGMHPKETTRQLSLAVKDGLIVETLTVGCKGSKAGIEQEGYWLPGDEISIKKKNTNKQEMGTYLRF

IVSRMKERAIDLNKKGKDNKHPMYRRLVHSAVDVPTIQEKVNEGKYRSYEEFKADAQLLLHNTVIFYGAD

SEQADIARMLYKDTCHELDELQLCKNCFYLSNARPDNWFCYPCIPNHELVWAKMKGFGFWPAKVMQKEDN

QVDVRFFGHHHQRAWIPSENIQDITVNIHRLHVKRSMGWKKACDELELHQRFLREGRFWKSKNEDRGEEE

AESSISSTSNEQLKVTQEPRAKKGRRNQSVEPKKEEPEPETEAVSSSQEIPTMPQPIEKVSVSTQTKKLS

ASSPRMLHRSTQTTNDGVCQSMCHDKYTKIFNDFKDRMKSDHKRETERVVREALEKLRSEMEEEKRQAVN

KAVANMQGEMDRKCKQVKEKCKEEFVEEIKKLATQHKQLISQTKKKQWCYNCEEEAMYHCCWNTSYCSIK

CQQEHWHAEHKRTCRRKR
