# Supplementary material for: Dynamic interaction of MYC enhancer RNA with YEATS2 protein regulates MYC gene transcription in pancreatic cancer
Source: EMBO Rep. 2025 Apr 11;26(10):2519–44. doi: 10.1038/s44319-025-00446-0 (PMC12117045; doi:10.1038/s44319-025-00446-0)
Supplement: Supplementary file 6 — Source data Fig. 2 [file 44319_2025_446_MOESM6_ESM.zip › Figure 2/2B/README.docx]

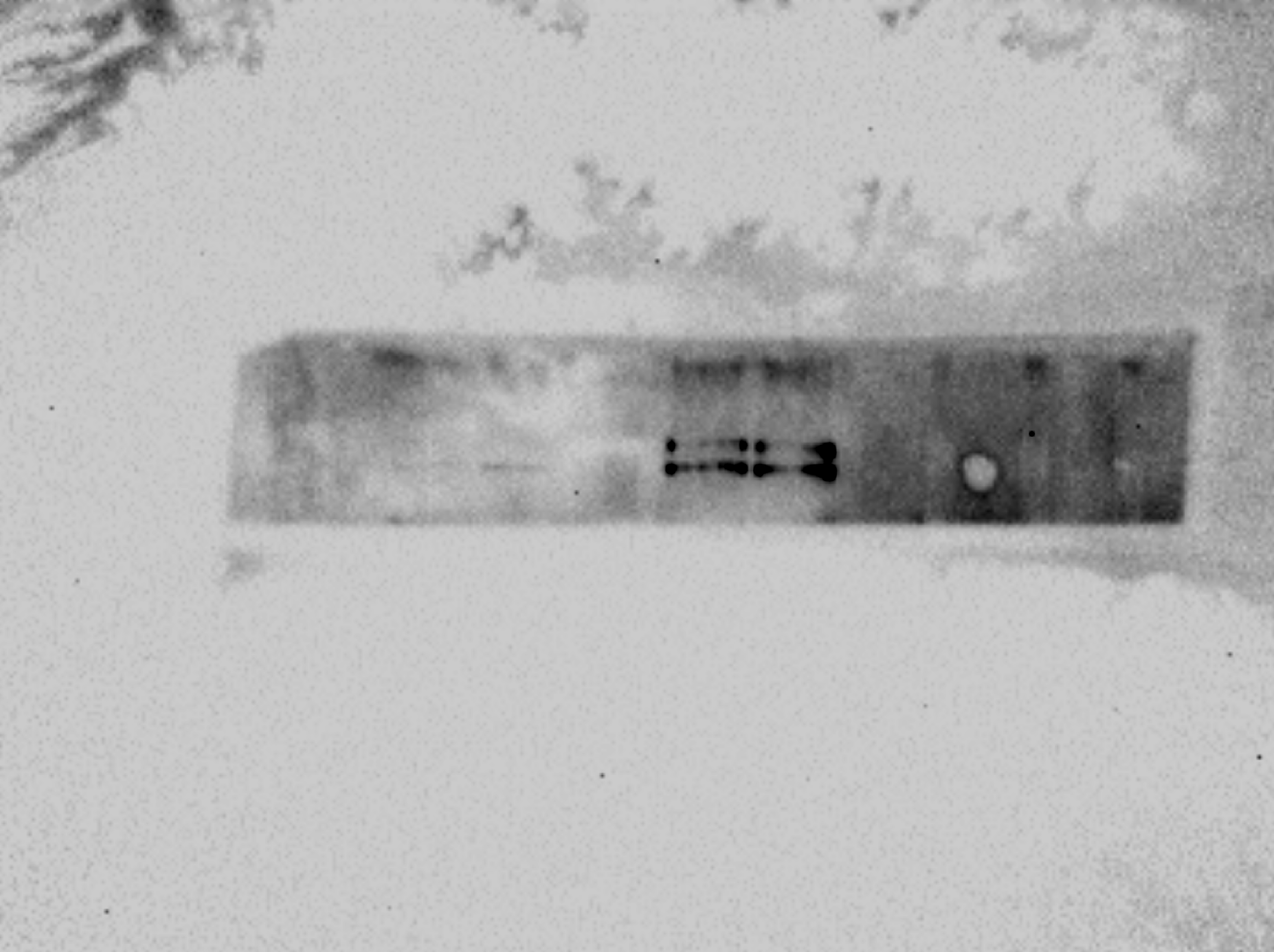


**70kDa**

**Lamin A/C in HPNE**


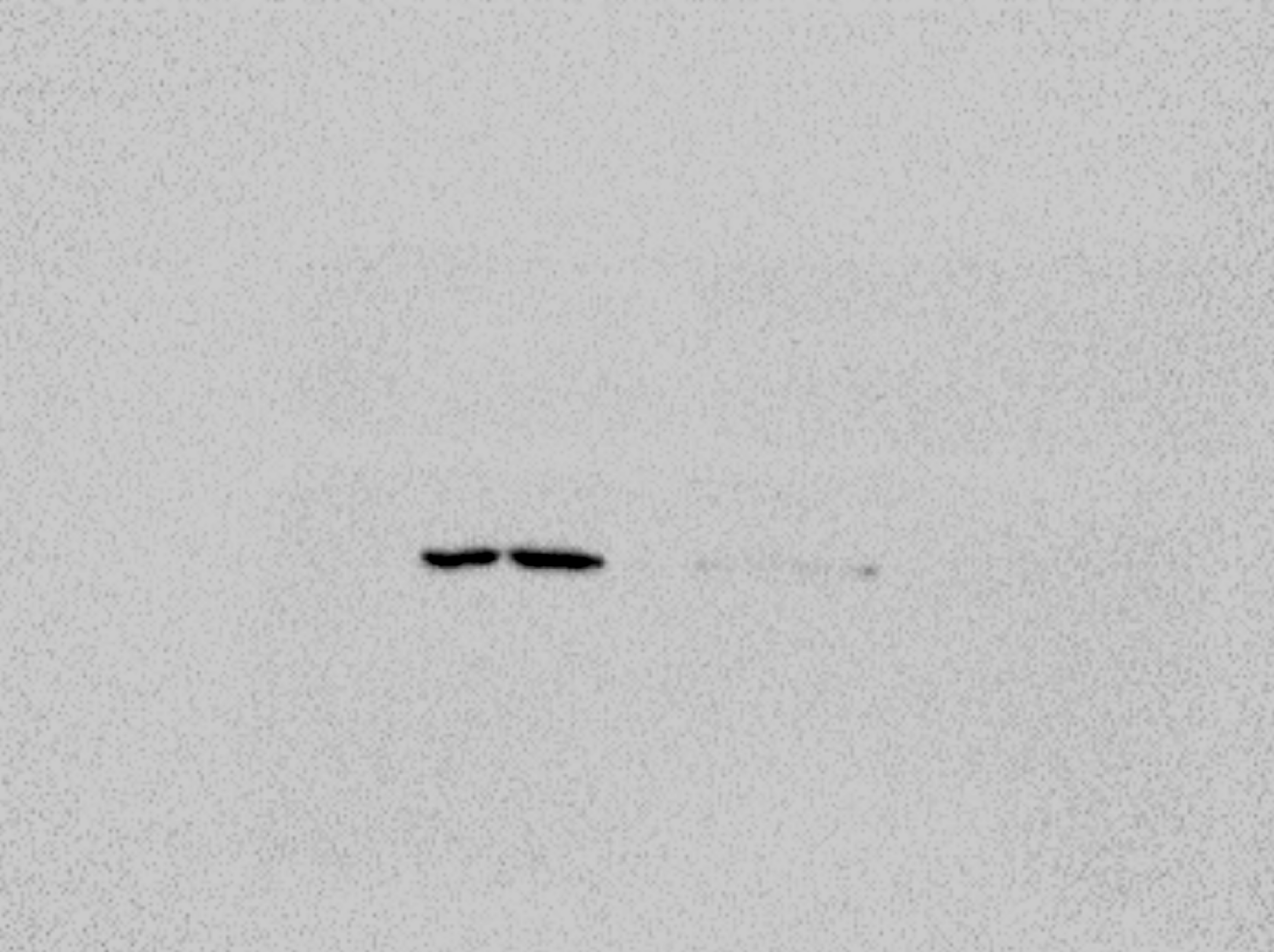


**37kDa**

**GAPDH in HPNE**

**Nuclear**

**0 24**

**0 24**

**TNF-α (h)**

**Cytosol**

**Lamin**

**GAPDH**

**0 24**

**0 24**

**Nuclear**

**Cytosol**

**TNF-α (h)**

**Fig 2B**
