# Supplementary figures and images for: Dynamic interaction of MYC enhancer RNA with YEATS2 protein regulates MYC gene transcription in pancreatic cancer
Source: EMBO Rep. 2025 Apr 11;26(10):2519–44. doi: 10.1038/s44319-025-00446-0 (PMC12117045; doi:10.1038/s44319-025-00446-0)

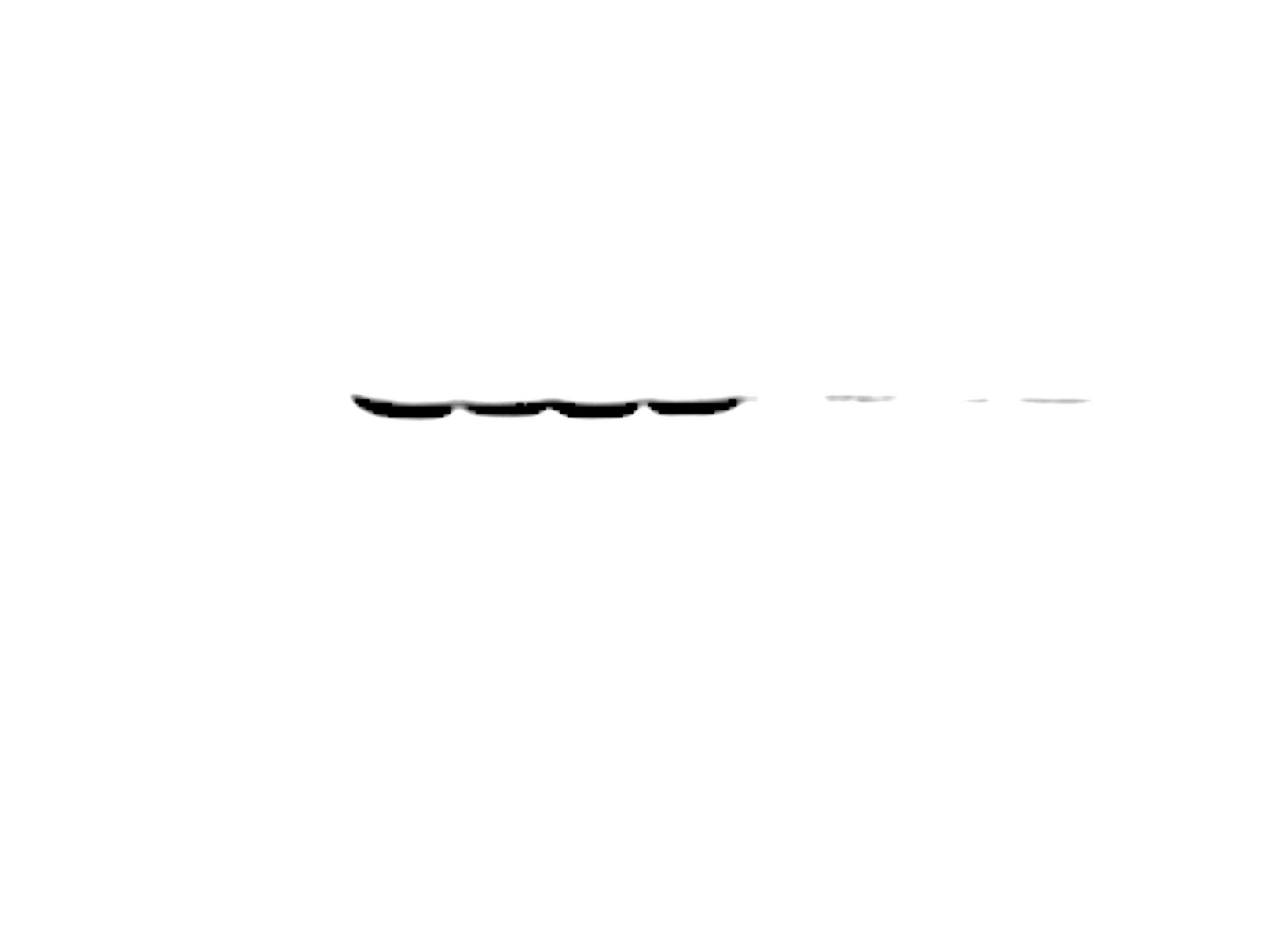

Supplement: Supplementary file 6 — Source data Fig. 2 [file 44319_2025_446_MOESM6_ESM.zip › Figure 2/2A/Western Blot beta-actin/Actin.tif]

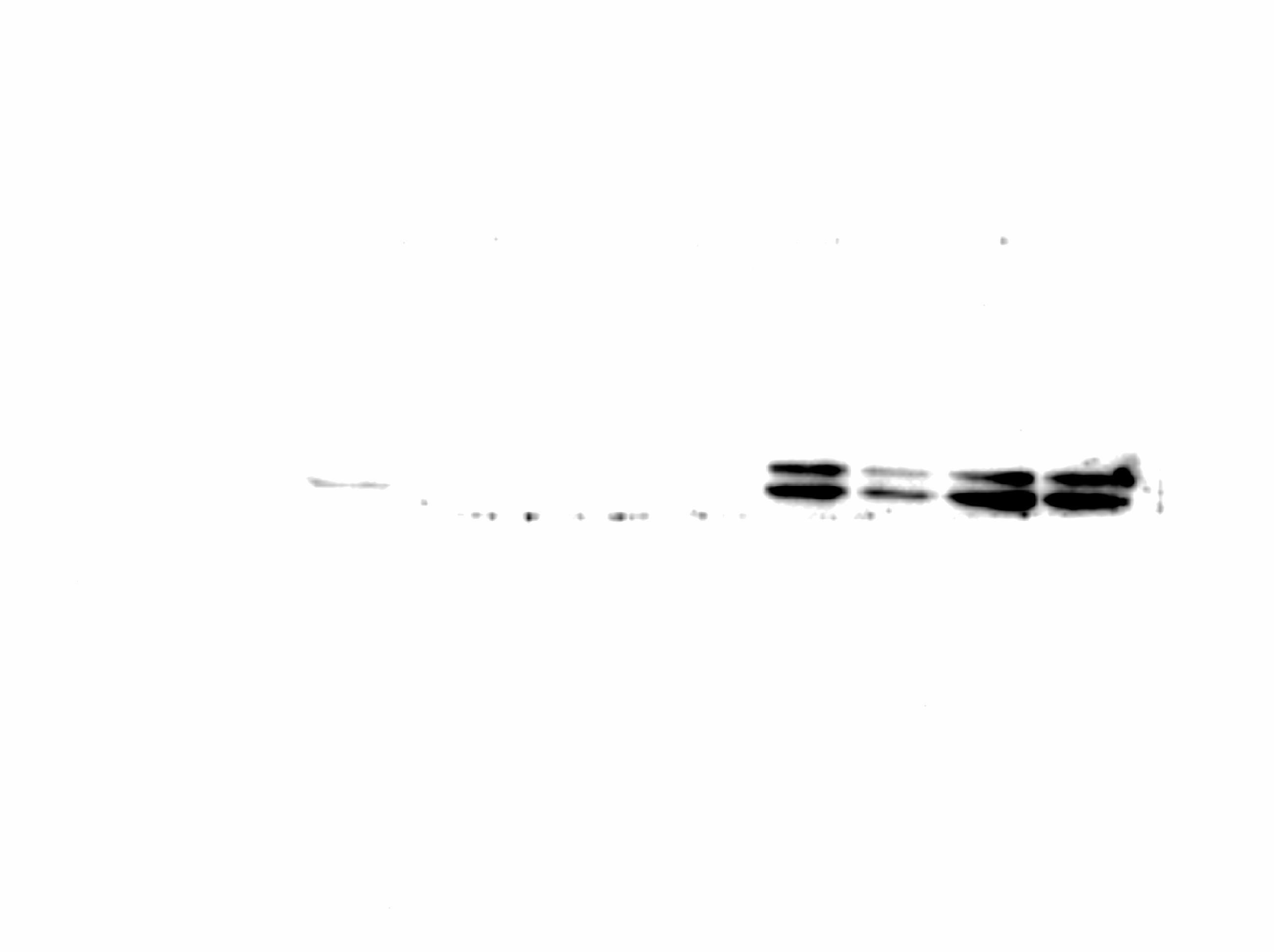

Supplement: Supplementary file 6 — Source data Fig. 2 [file 44319_2025_446_MOESM6_ESM.zip › Figure 2/2A/Western Blot Lamin/Lamin.tif]

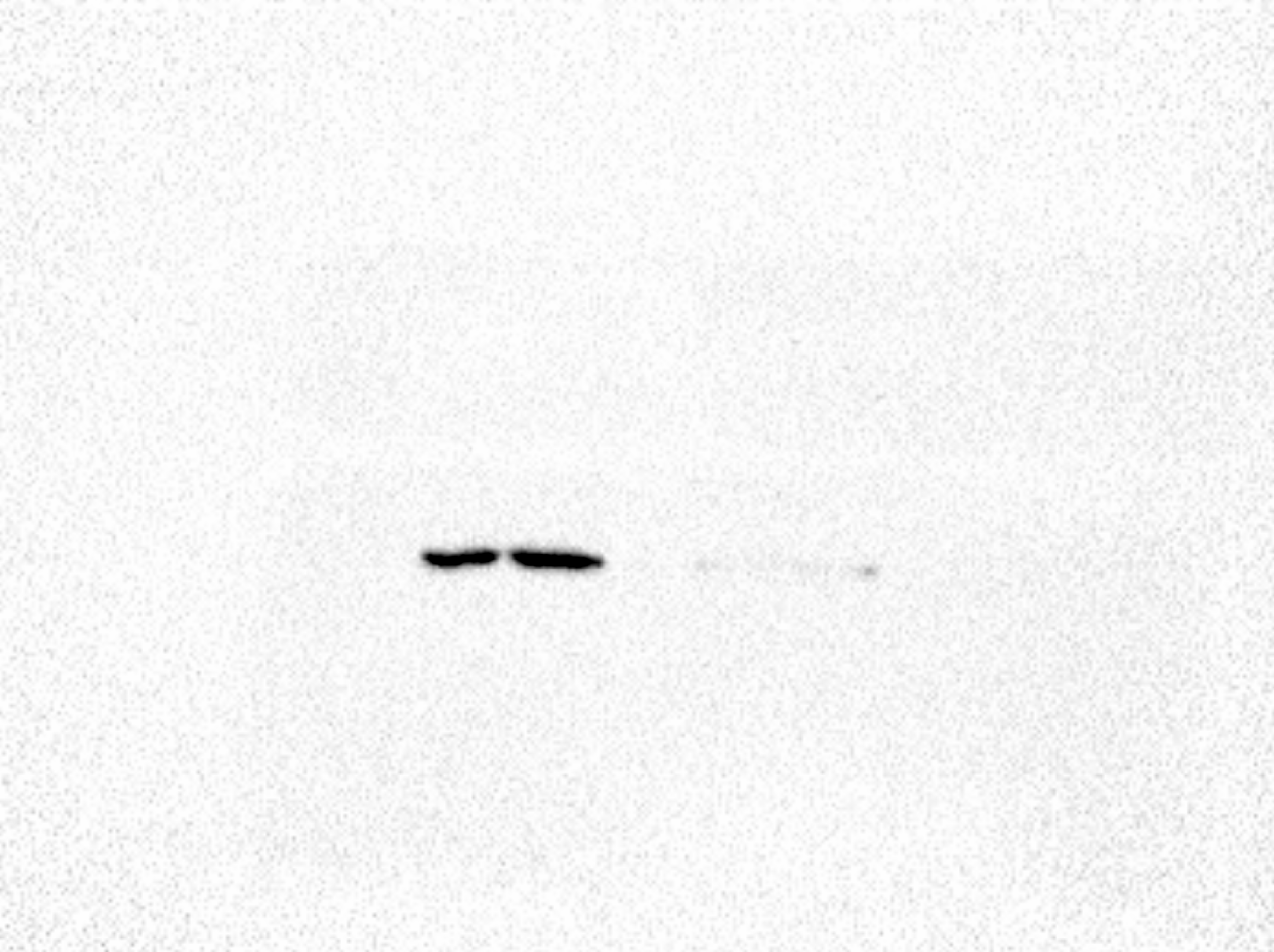

Supplement: Supplementary file 6 — Source data Fig. 2 [file 44319_2025_446_MOESM6_ESM.zip › Figure 2/2B/Western Blot GAPDH/GAPDH.tif]

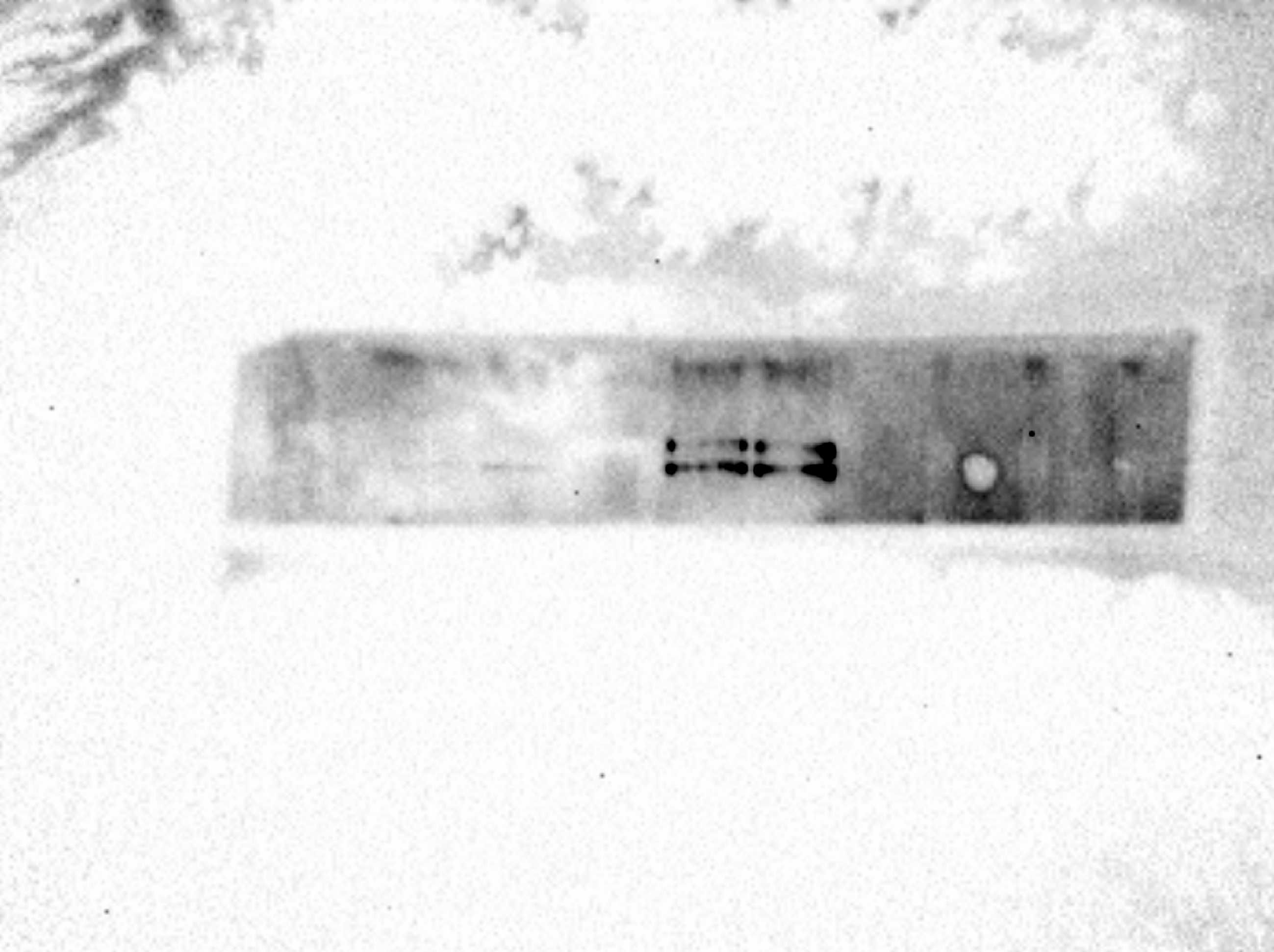

Supplement: Supplementary file 6 — Source data Fig. 2 [file 44319_2025_446_MOESM6_ESM.zip › Figure 2/2B/Western Blot Lamin/lamin.tif]

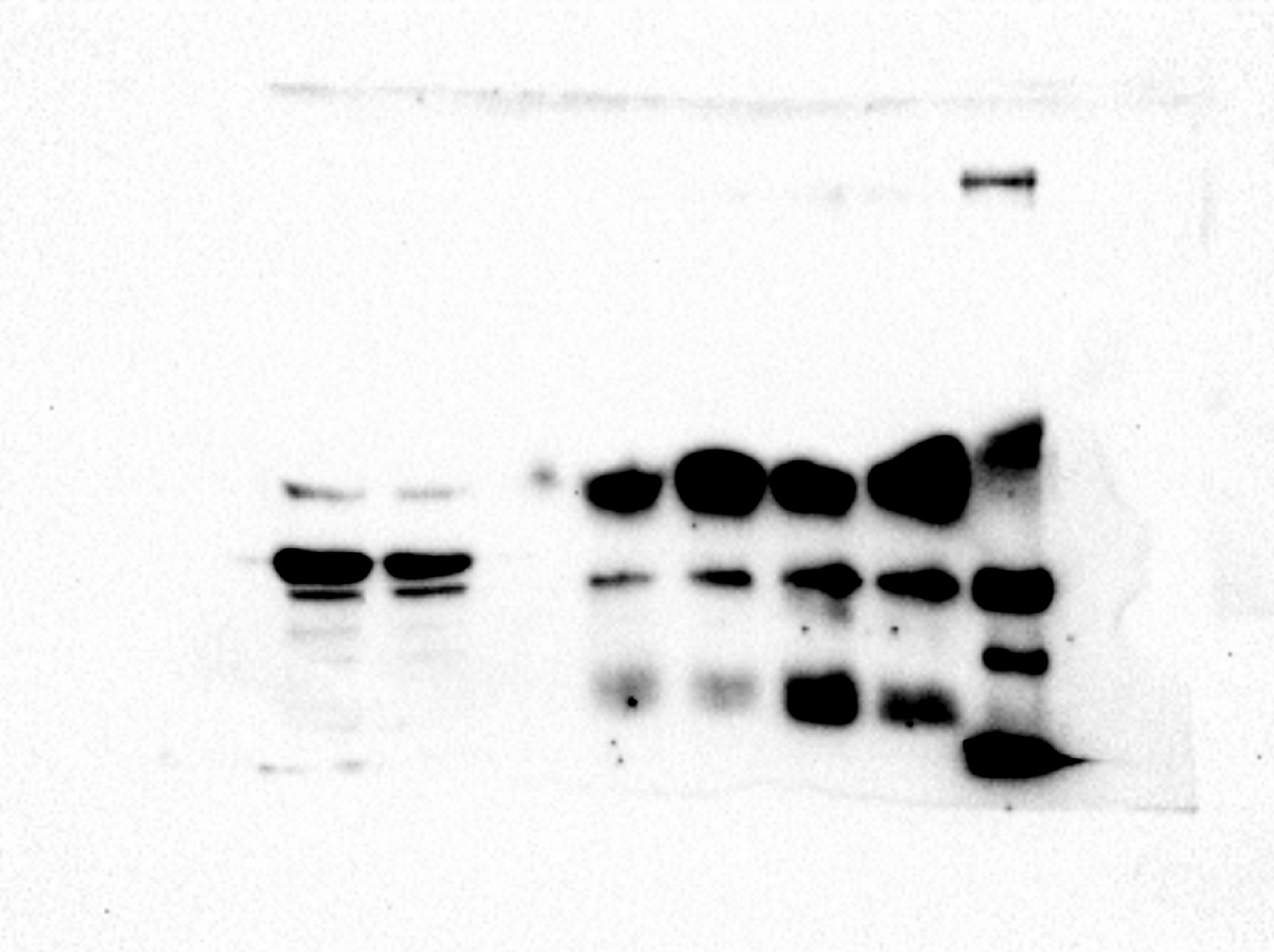

Supplement: Supplementary file 7 — Source data Fig. 3 [file 44319_2025_446_MOESM7_ESM.zip › Figure 3/3A/Western Blot GAPDH/GAPDH.tif]

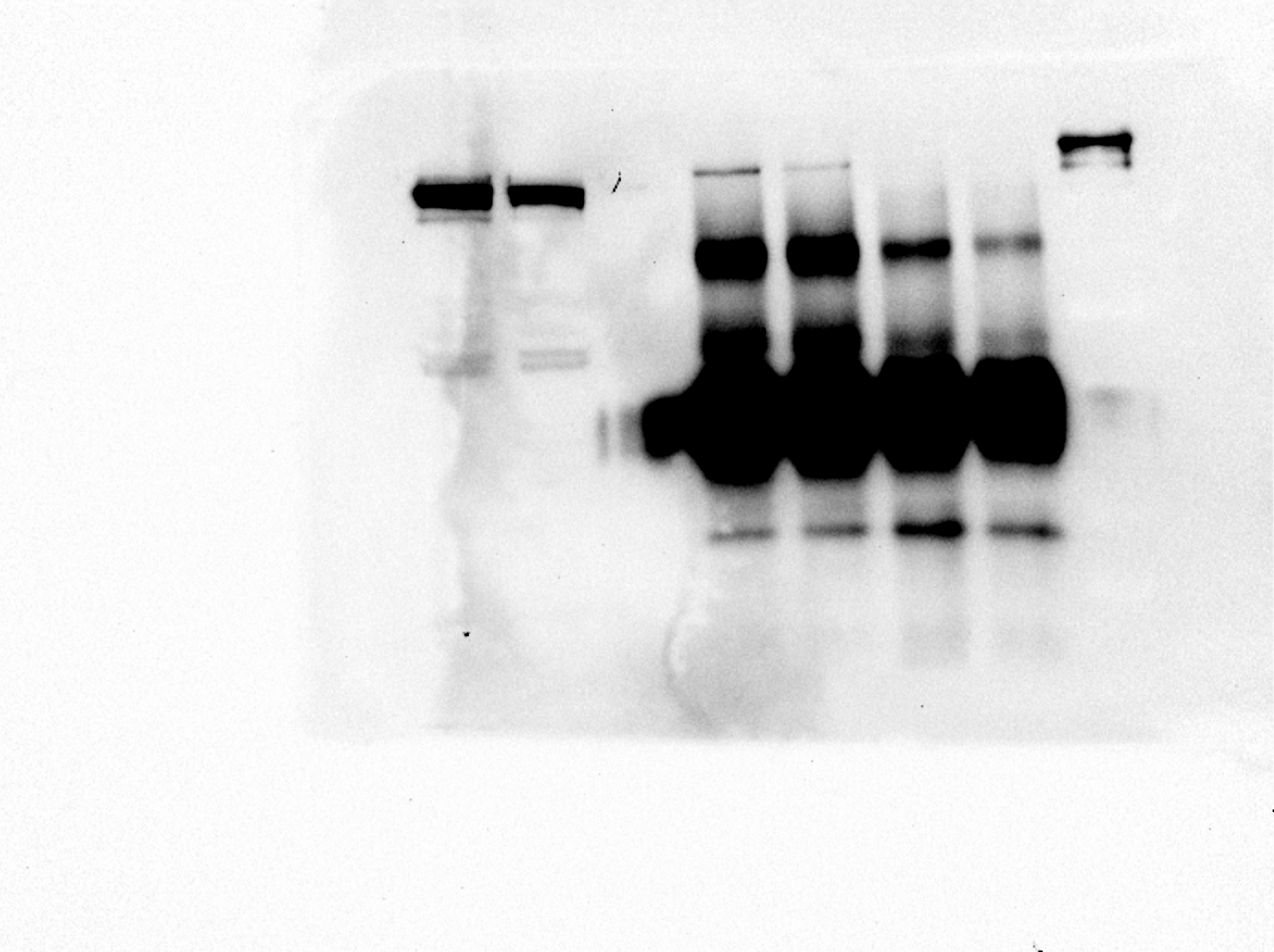

Supplement: Supplementary file 7 — Source data Fig. 3 [file 44319_2025_446_MOESM7_ESM.zip › Figure 3/3A/Western Blot YEATS2/YEATS2.tif]

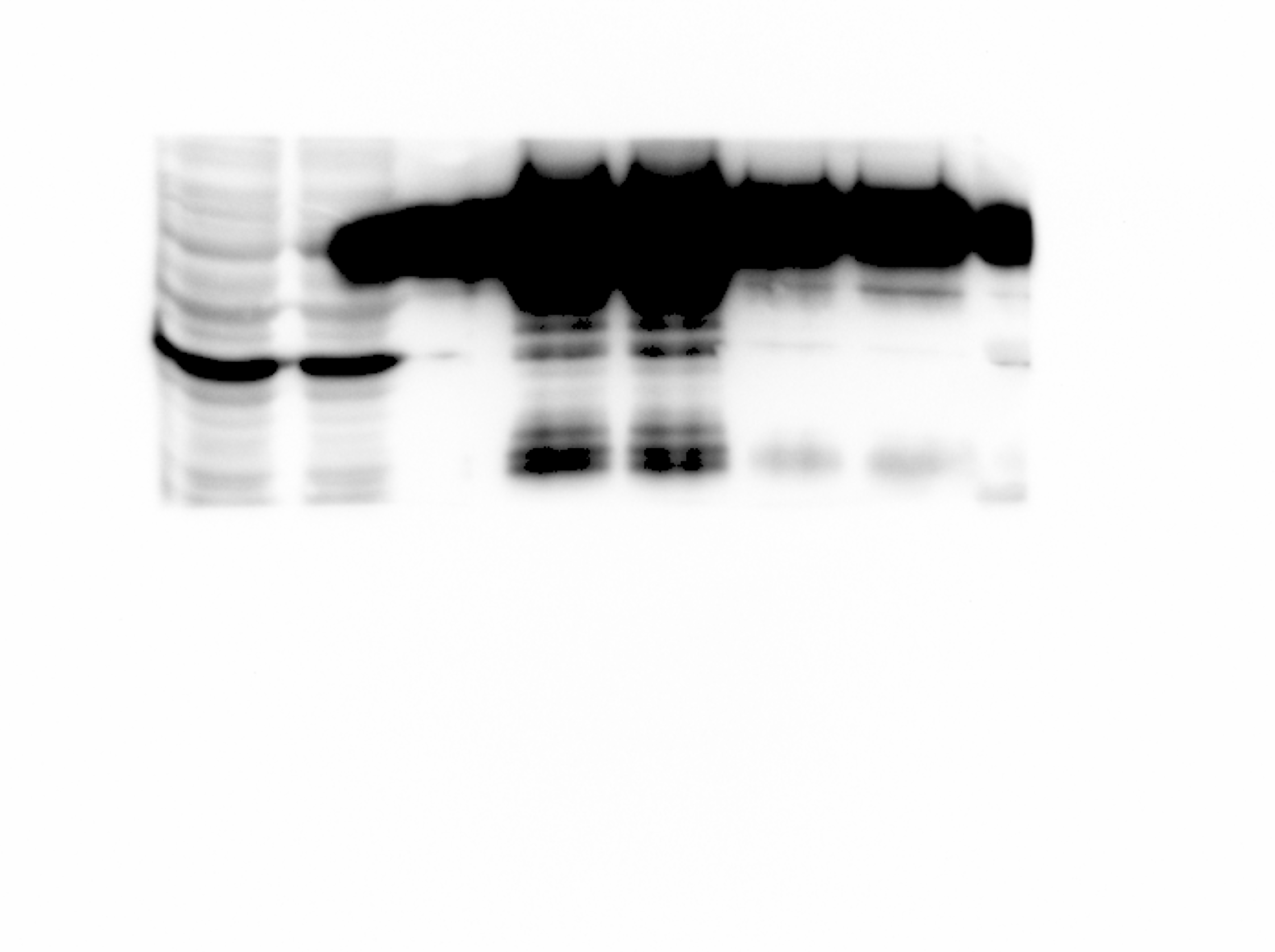

Supplement: Supplementary file 7 — Source data Fig. 3 [file 44319_2025_446_MOESM7_ESM.zip › Figure 3/3B/Western Blot GAPDH/GAPDH.tif]

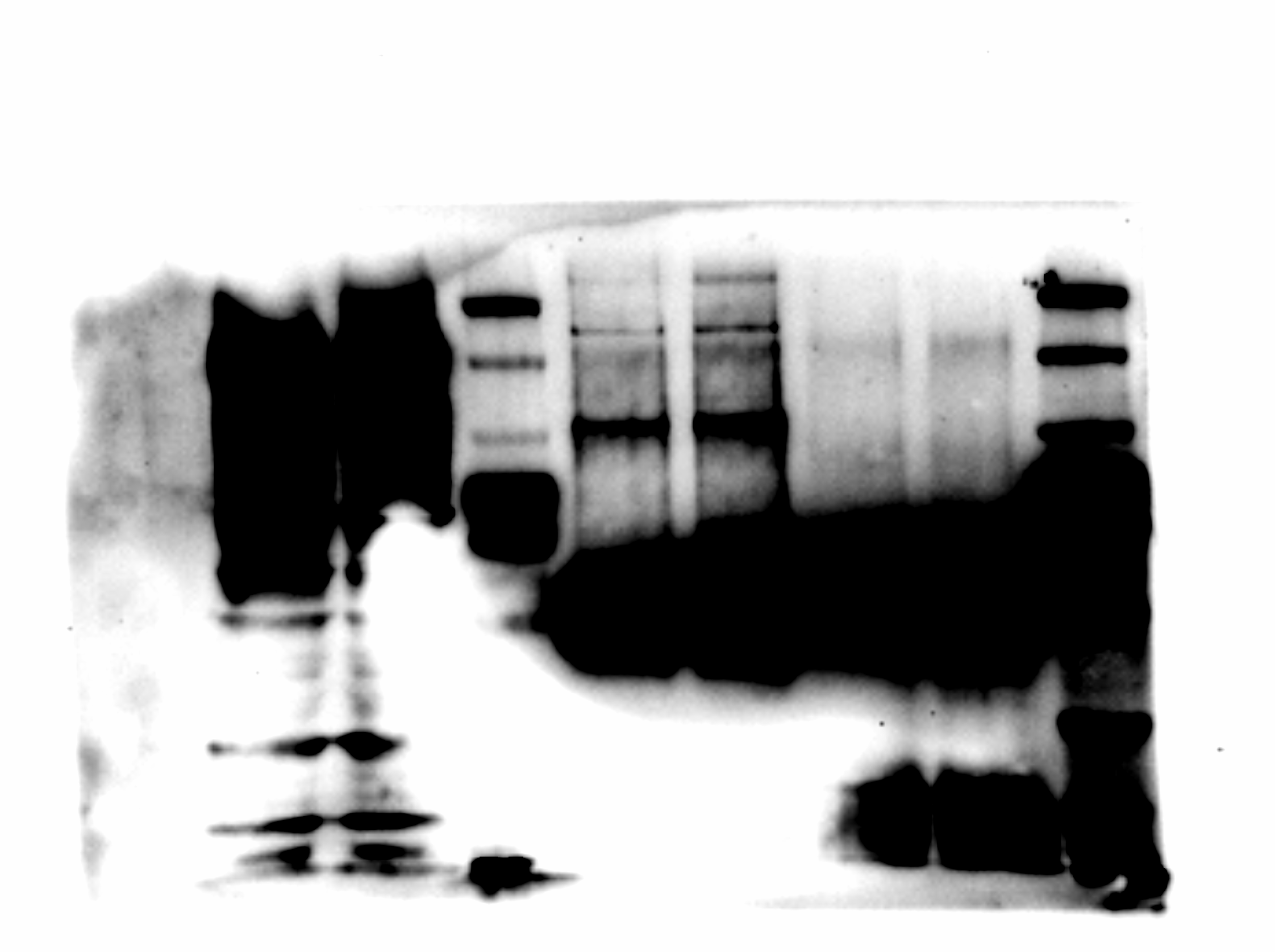

Supplement: Supplementary file 7 — Source data Fig. 3 [file 44319_2025_446_MOESM7_ESM.zip › Figure 3/3B/Western Blot YEATS2/Yeats2.tif]

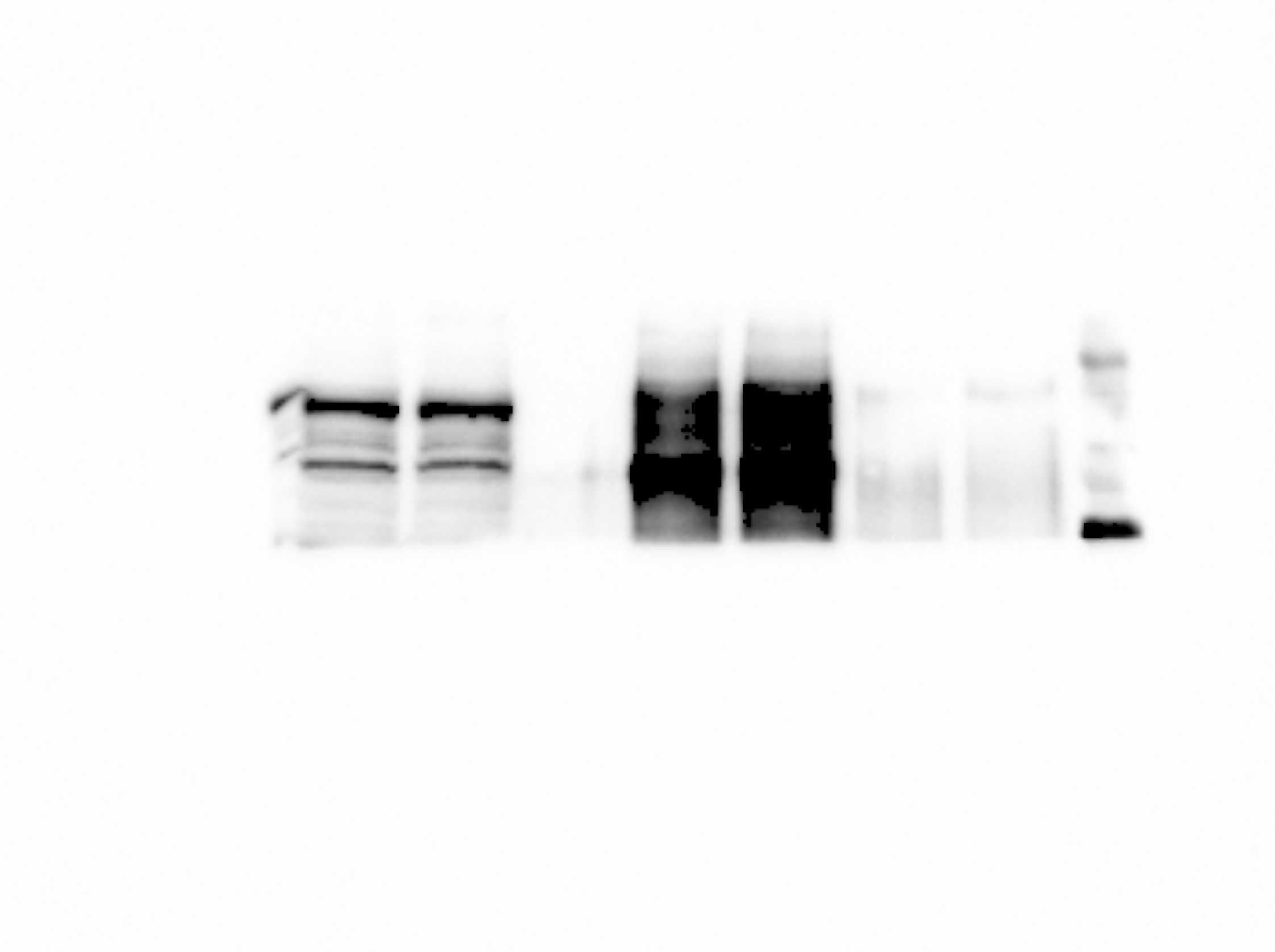

Supplement: Supplementary file 7 — Source data Fig. 3 [file 44319_2025_446_MOESM7_ESM.zip › Figure 3/3B/Western Input YEATS2/Yeats2_Input_Final.tif]

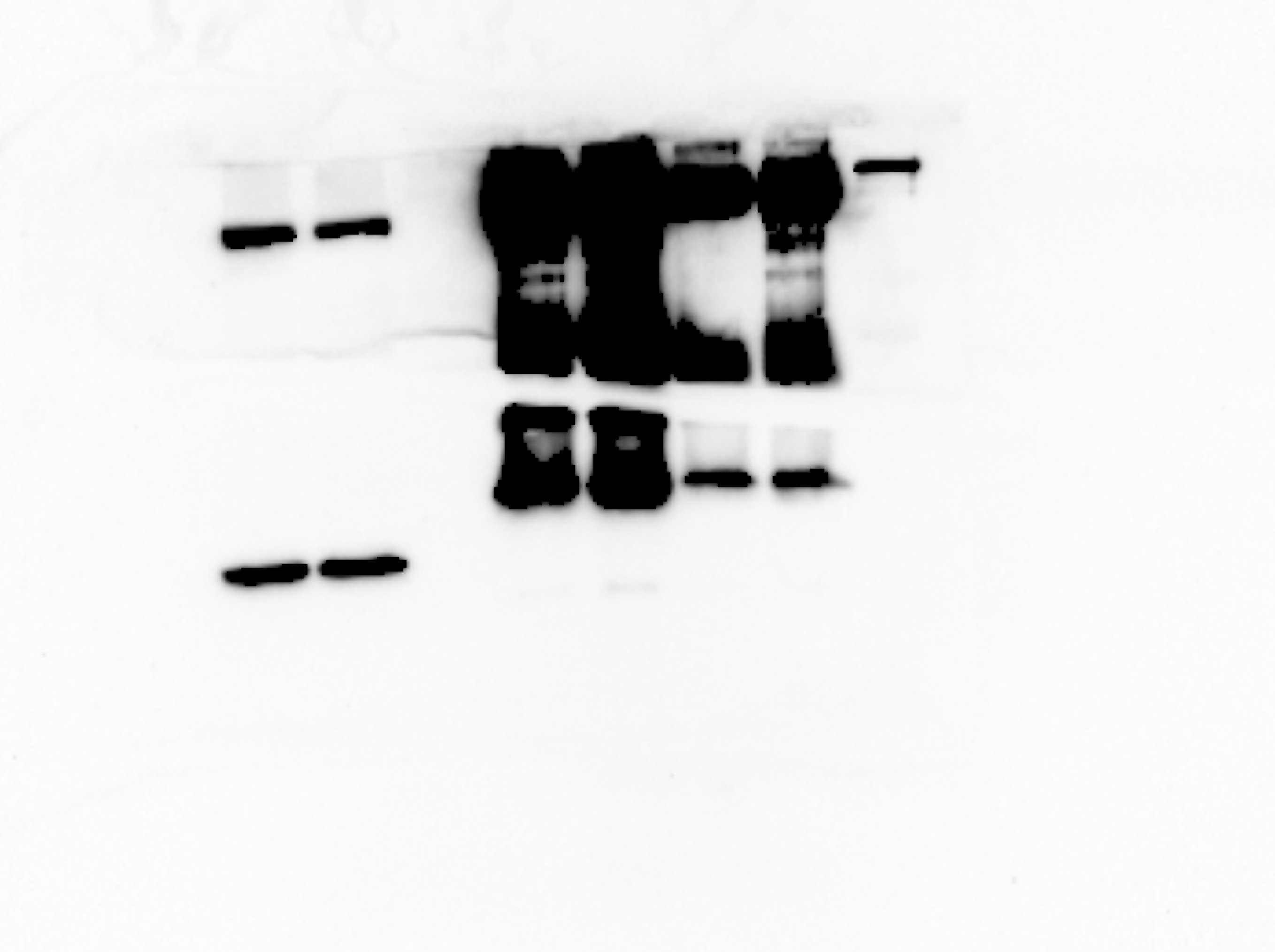

Supplement: Supplementary file 7 — Source data Fig. 3 [file 44319_2025_446_MOESM7_ESM.zip › Figure 3/3C/Western Blot GAPDH/GAPDH_Final.tif]

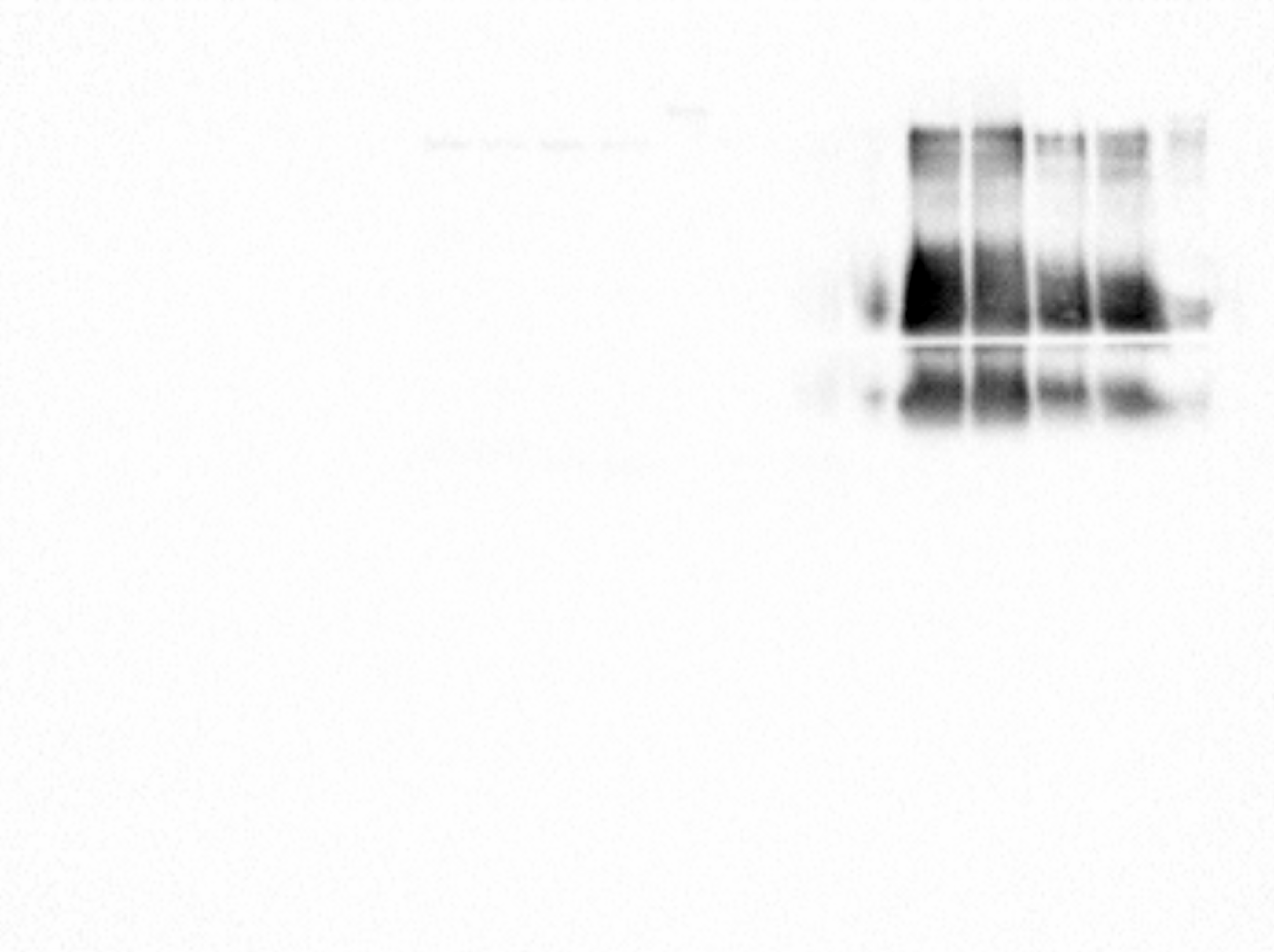

Supplement: Supplementary file 7 — Source data Fig. 3 [file 44319_2025_446_MOESM7_ESM.zip › Figure 3/3C/Western Blot YEATS2 IP/YEATS2.tif]

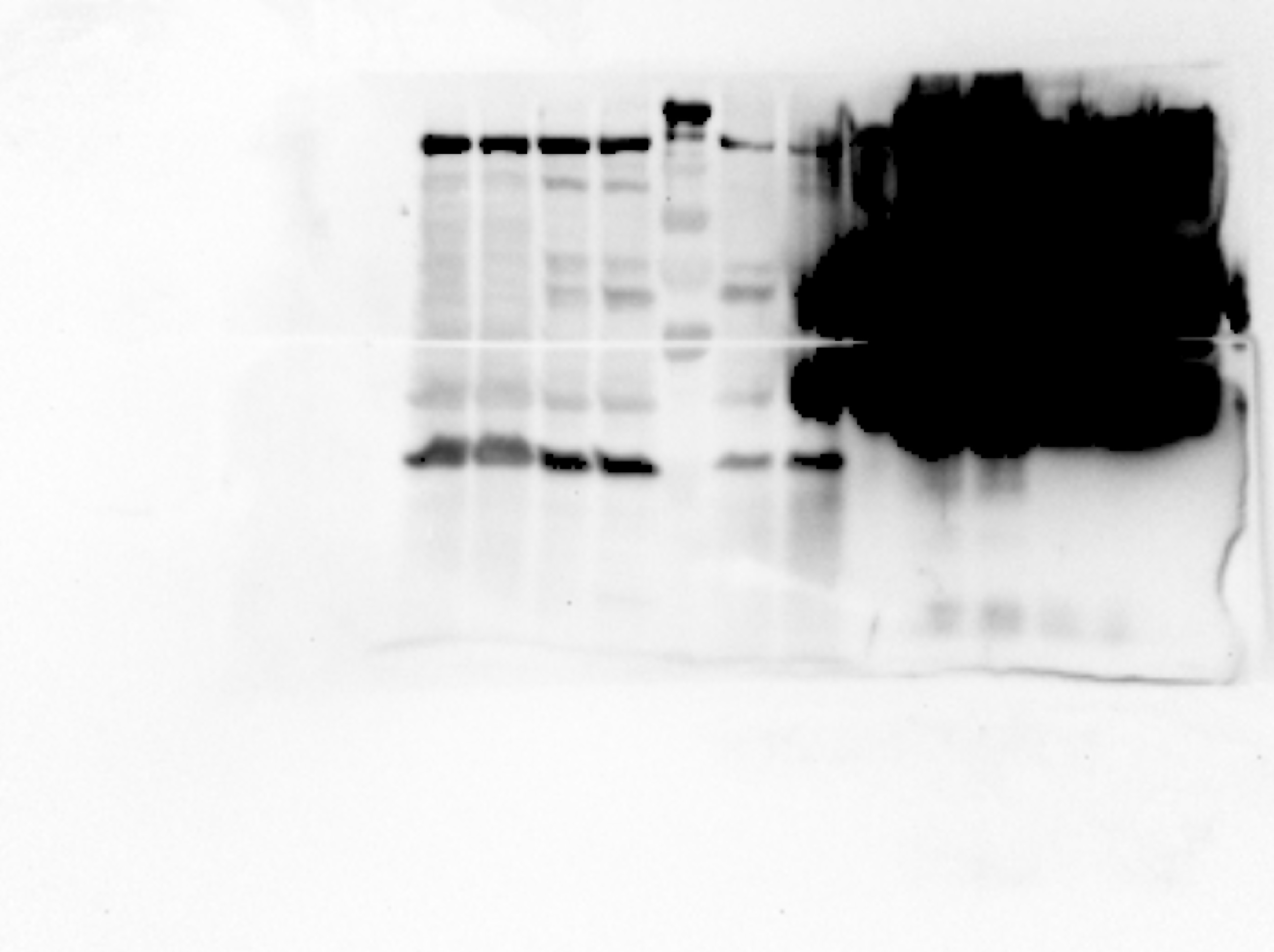

Supplement: Supplementary file 7 — Source data Fig. 3 [file 44319_2025_446_MOESM7_ESM.zip › Figure 3/3D/Western Blot YEATS2 and GAPDH/YEATS2 and GAPDH.tif]

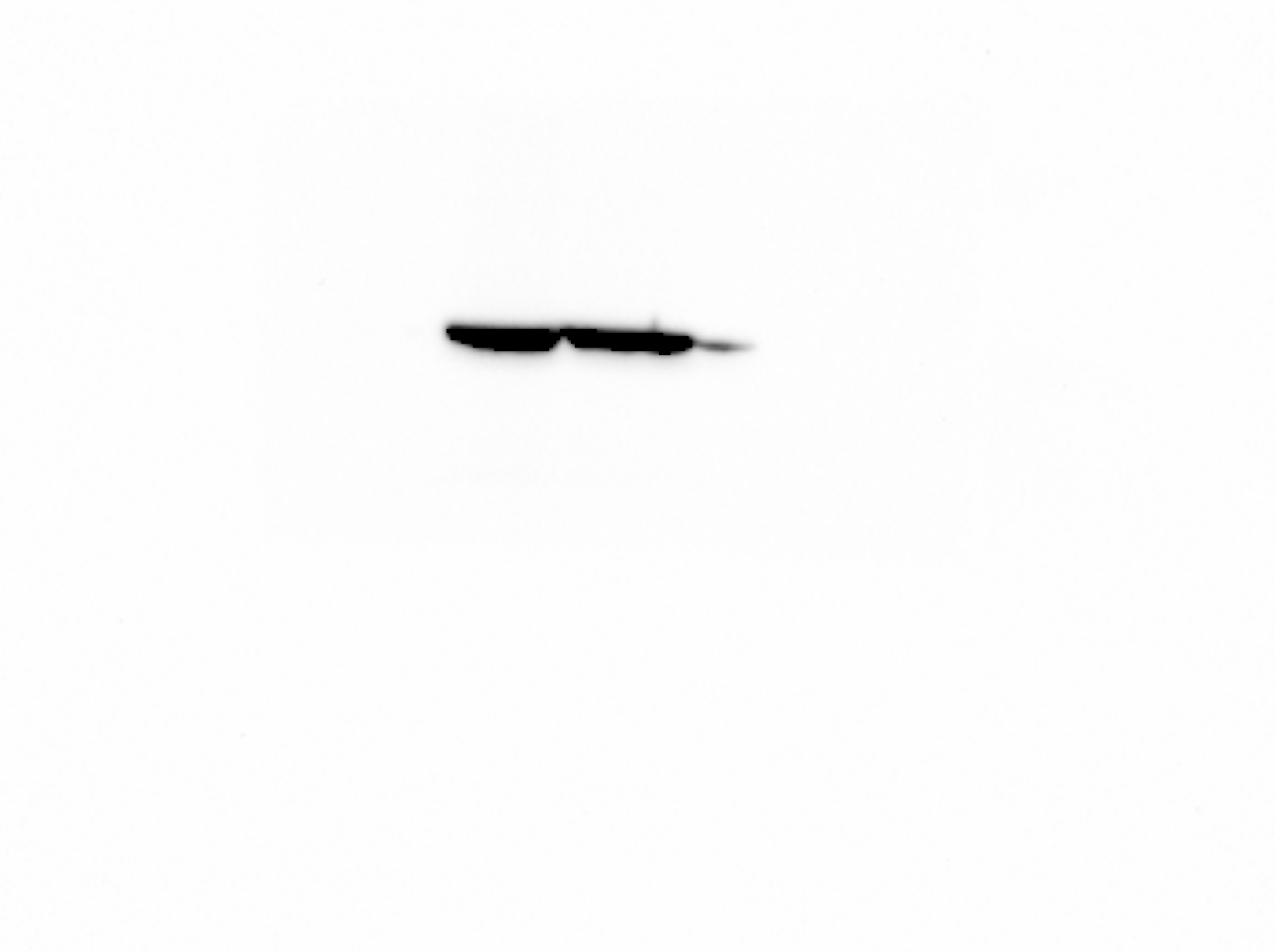

Supplement: Supplementary file 7 — Source data Fig. 3 [file 44319_2025_446_MOESM7_ESM.zip › Figure 3/3E/Western Blot GAPDH/30SEC GAPDH.tif]

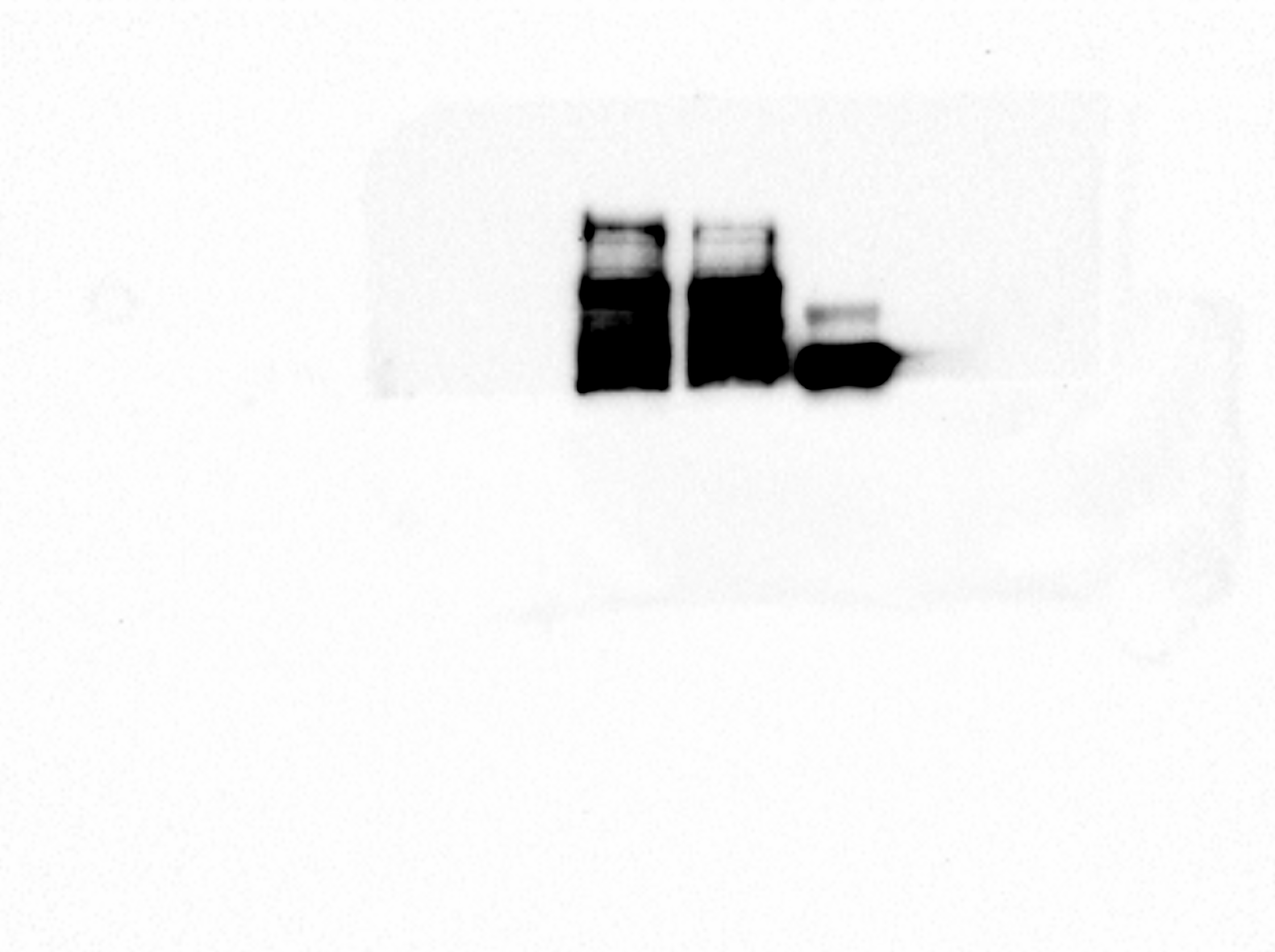

Supplement: Supplementary file 7 — Source data Fig. 3 [file 44319_2025_446_MOESM7_ESM.zip › Figure 3/3E/Western Blot YEATS2/YEATS2 .tif]

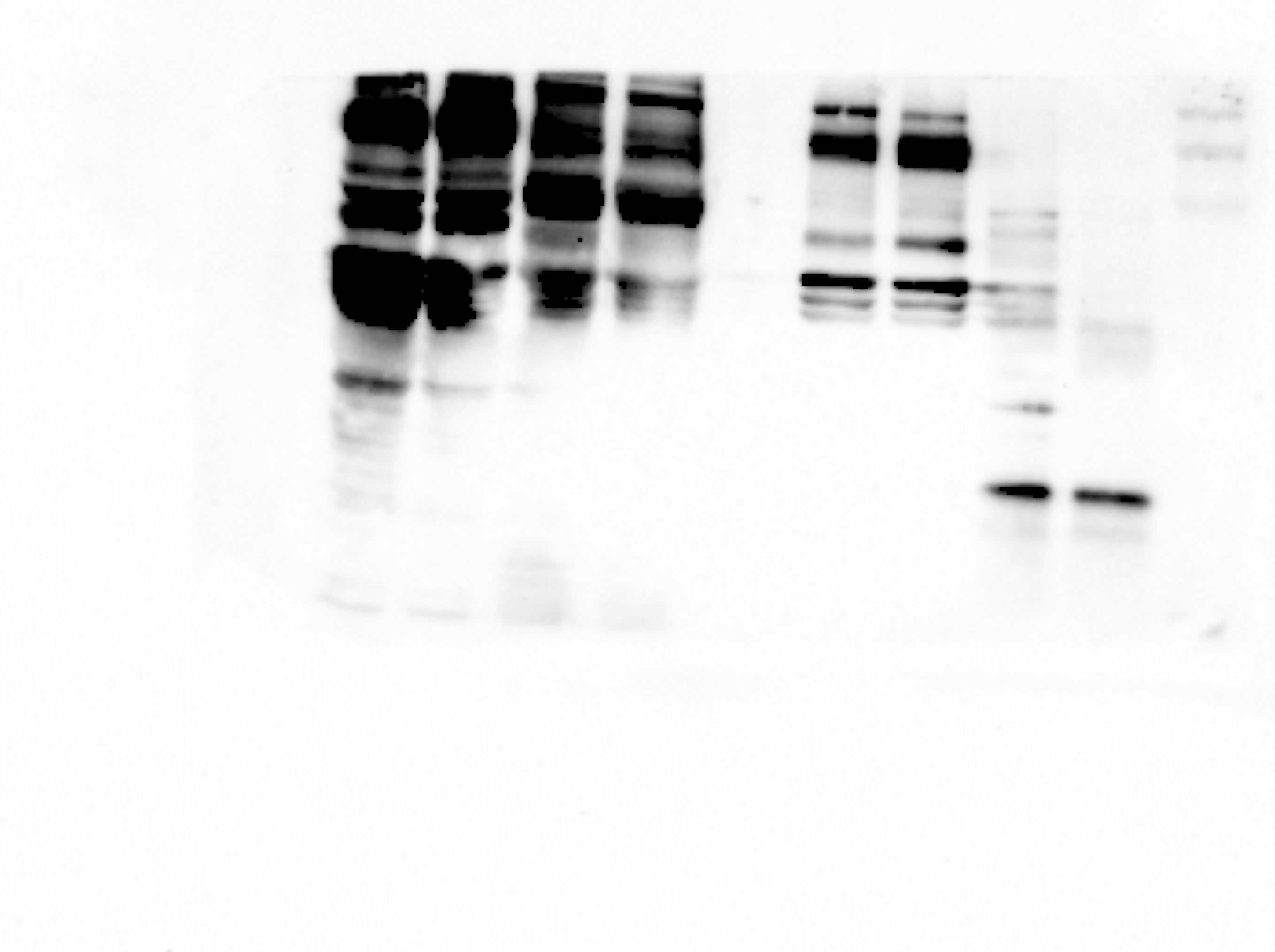

Supplement: Supplementary file 7 — Source data Fig. 3 [file 44319_2025_446_MOESM7_ESM.zip › Figure 3/3F/Western Blot 4G10/4G10.tif]

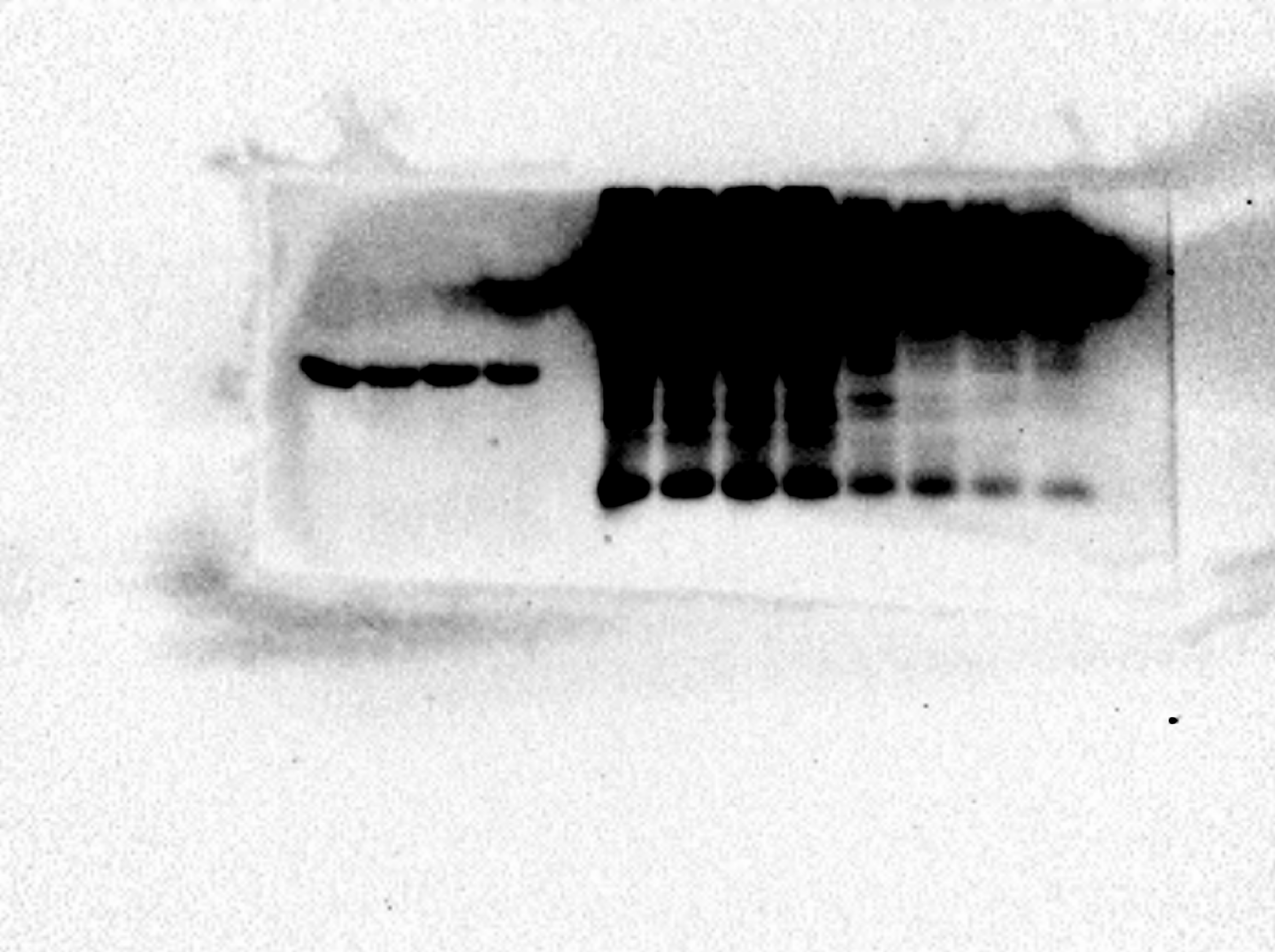

Supplement: Supplementary file 7 — Source data Fig. 3 [file 44319_2025_446_MOESM7_ESM.zip › Figure 3/3F/Western Blot GAPDH/GAPDH.tif]

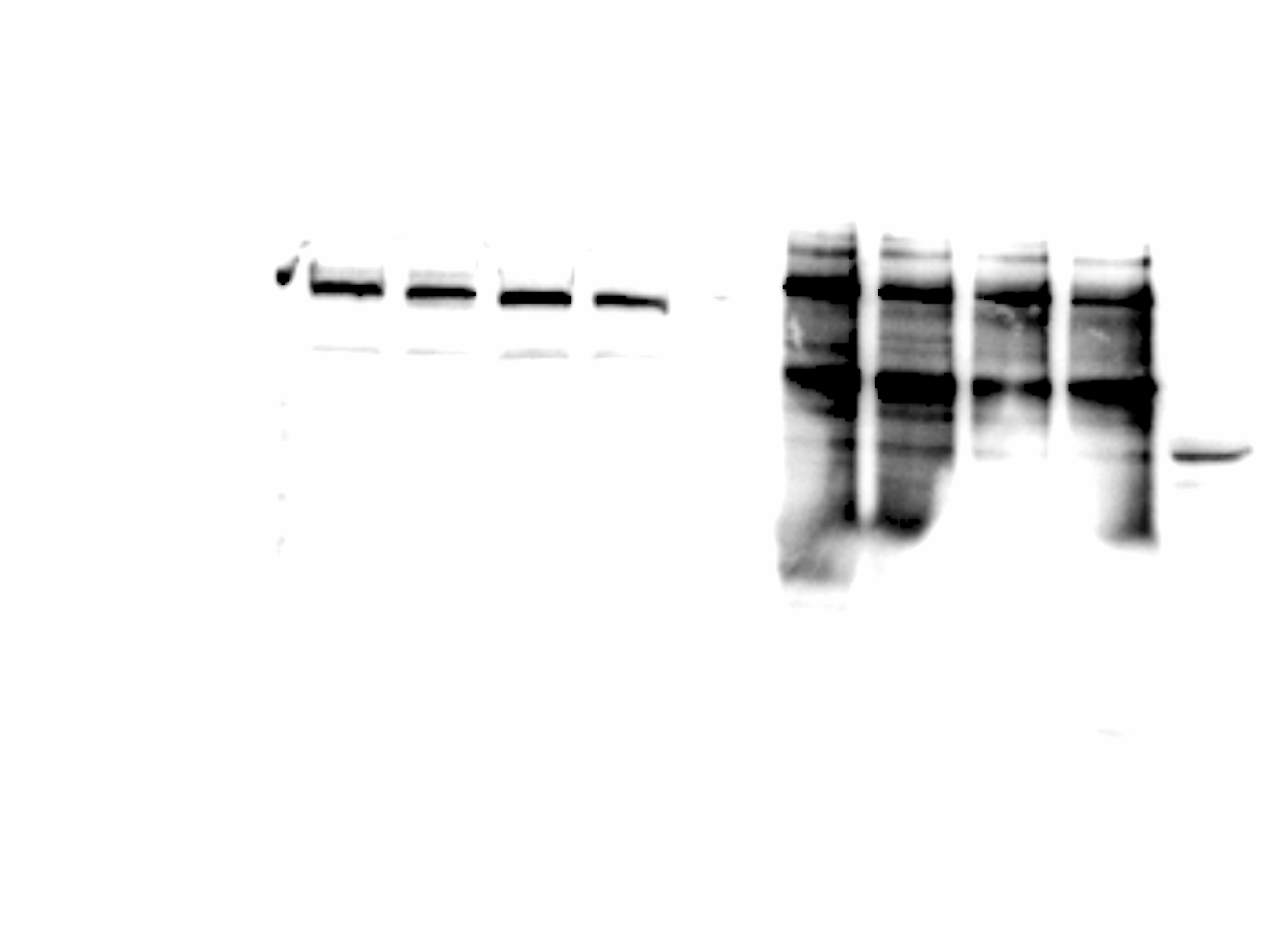

Supplement: Supplementary file 7 — Source data Fig. 3 [file 44319_2025_446_MOESM7_ESM.zip › Figure 3/3F/Western Blot Input YEATS2/YEATS2_Input_Final.tif]

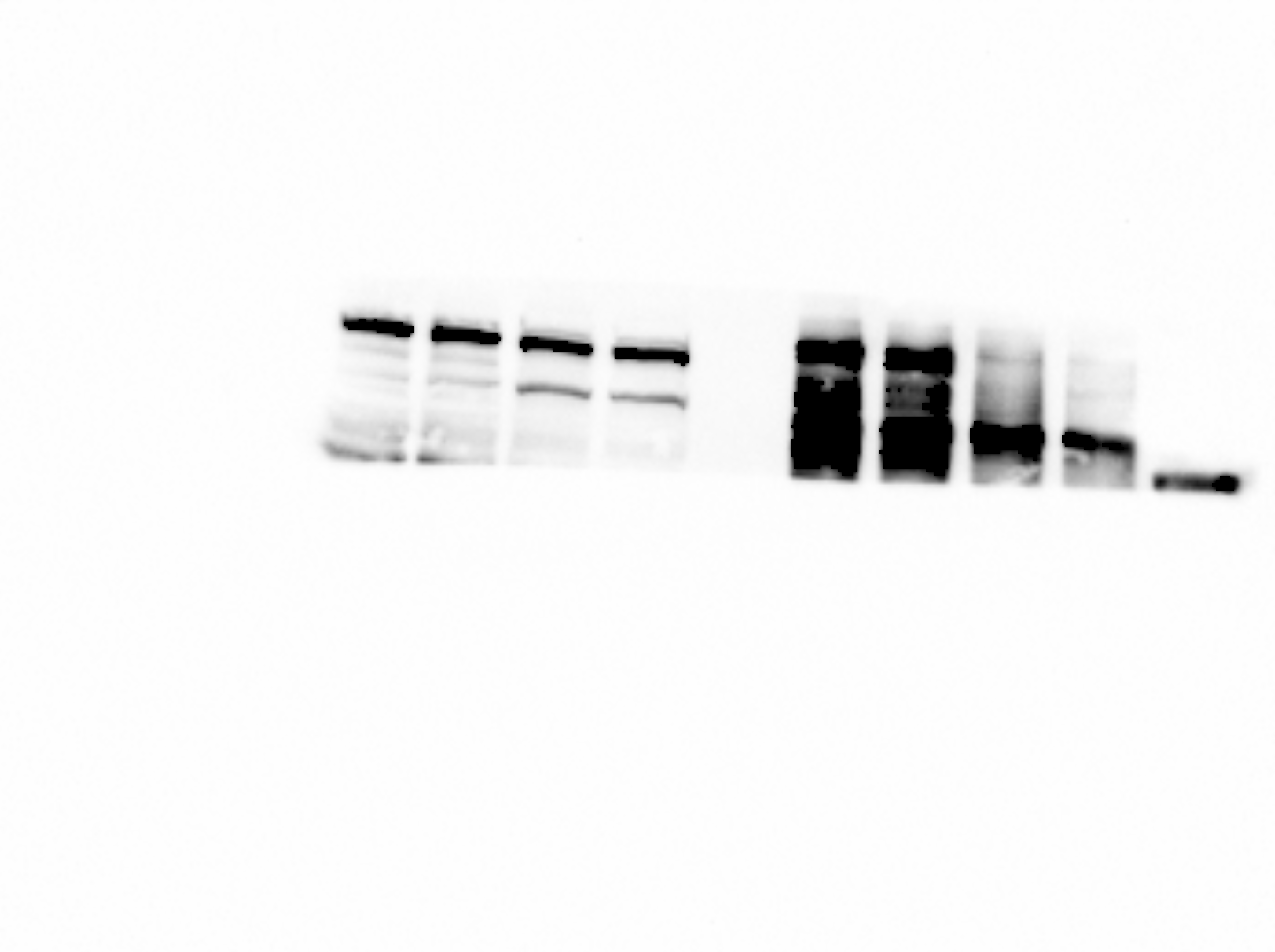

Supplement: Supplementary file 7 — Source data Fig. 3 [file 44319_2025_446_MOESM7_ESM.zip › Figure 3/3F/Western Blot YEATS2 IP/YEATS2.tif]

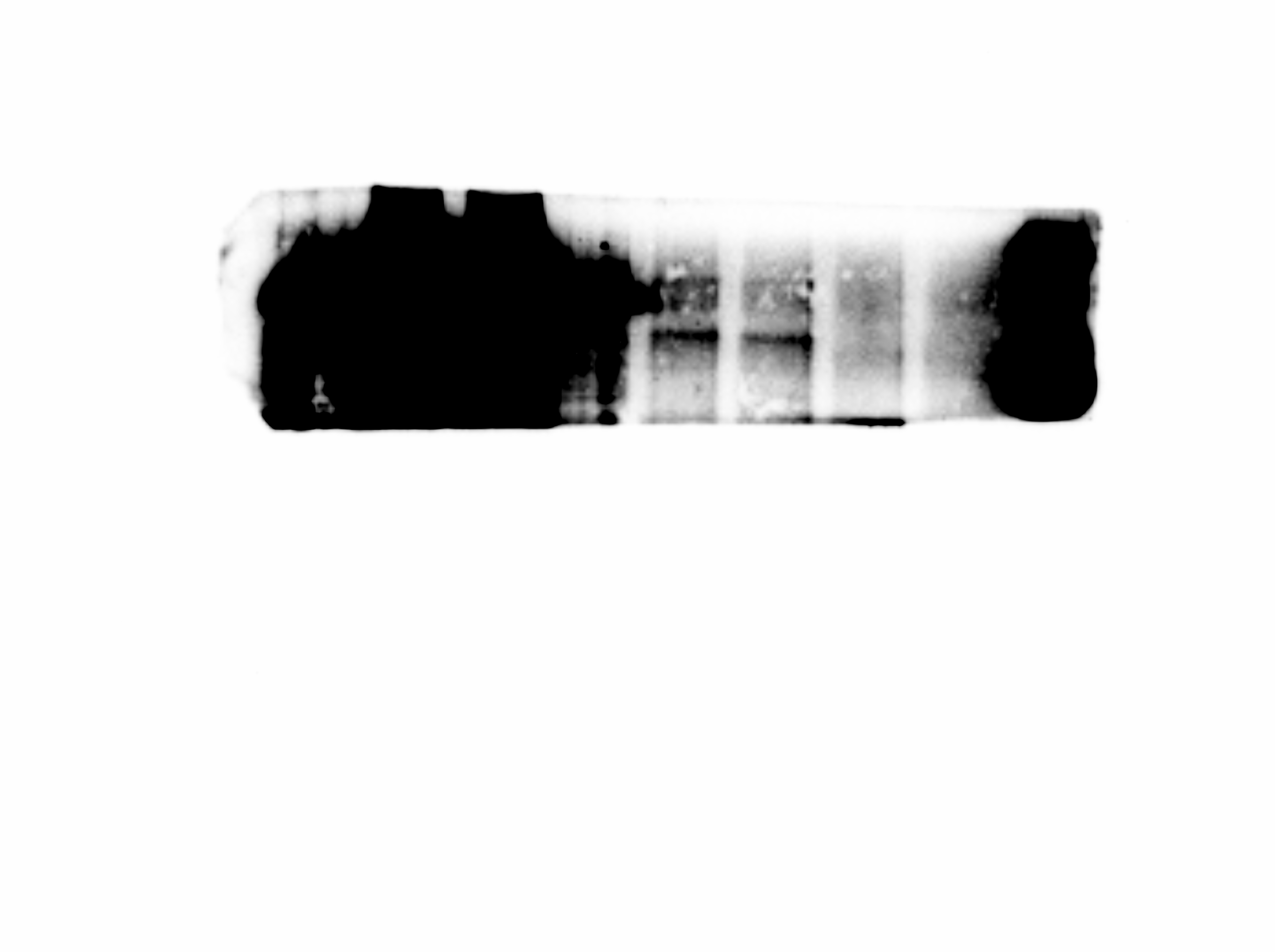

Supplement: Supplementary file 7 — Source data Fig. 3 [file 44319_2025_446_MOESM7_ESM.zip › Figure 3/3G/Western Blot 4G10/4G10.tif]

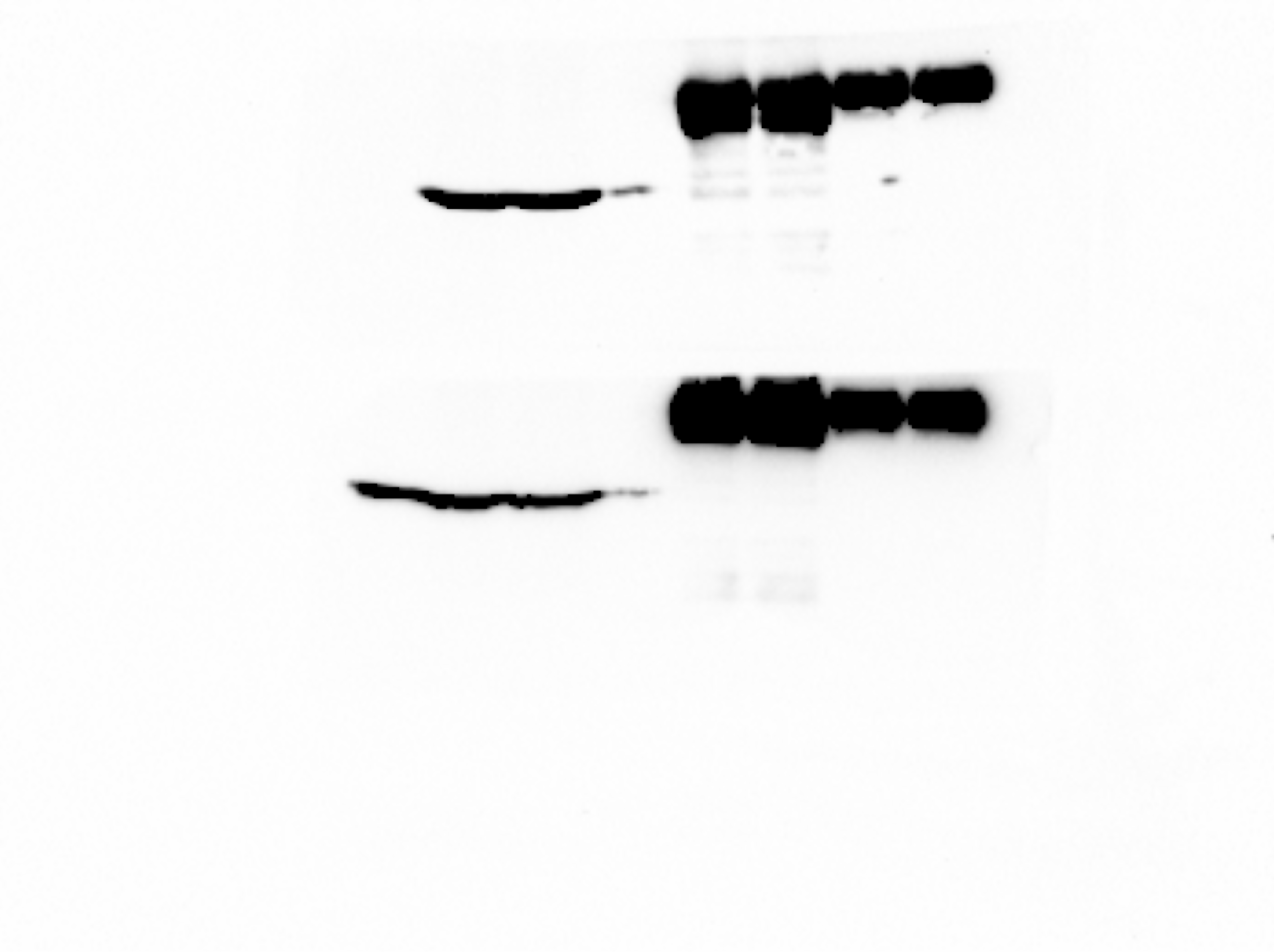

Supplement: Supplementary file 7 — Source data Fig. 3 [file 44319_2025_446_MOESM7_ESM.zip › Figure 3/3G/Western Blot GAPDH/GAPDH .tif]

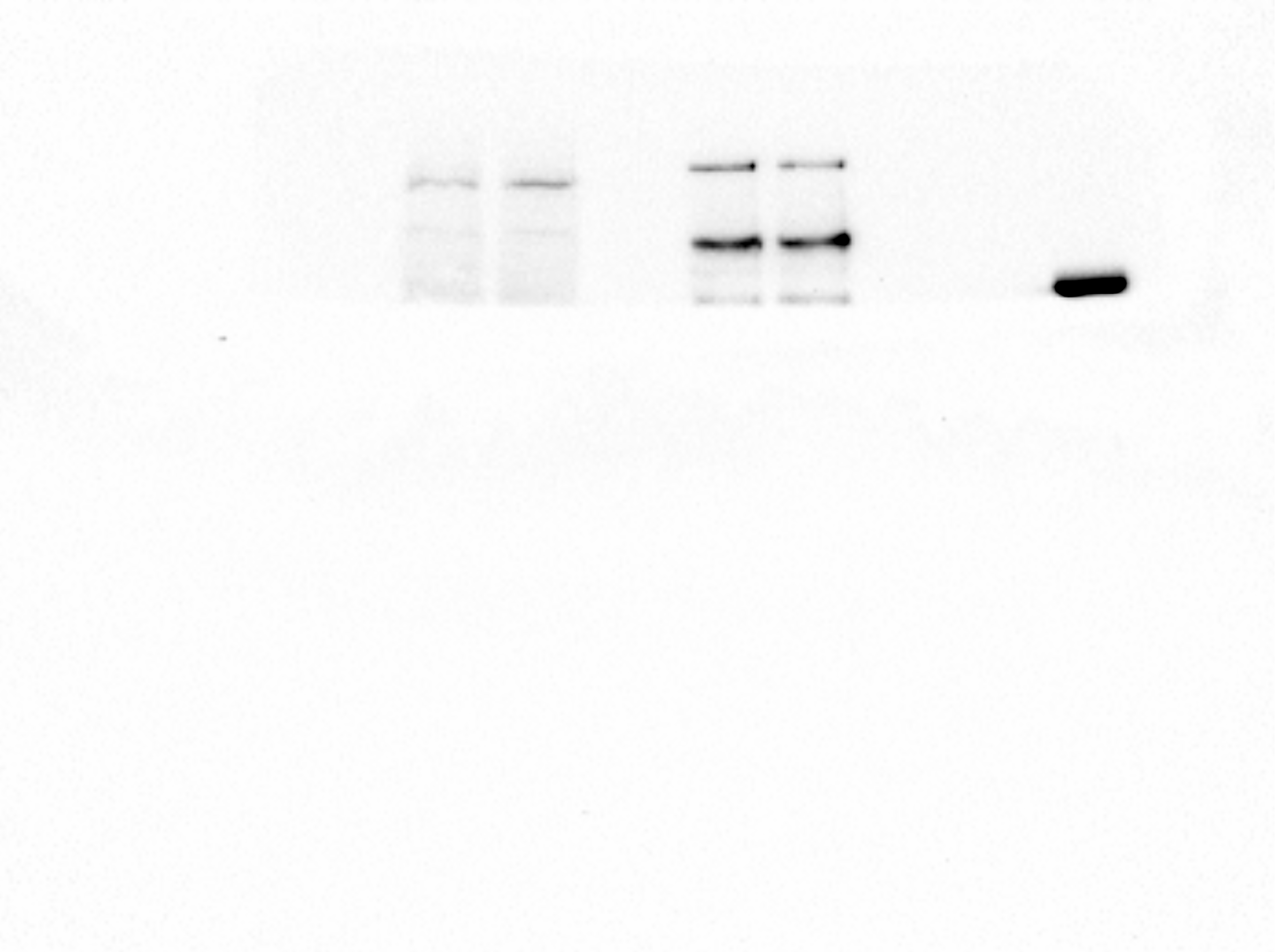

Supplement: Supplementary file 7 — Source data Fig. 3 [file 44319_2025_446_MOESM7_ESM.zip › Figure 3/3G/Western Blot Input YEATS2/YEATS2 INPUT.tif]

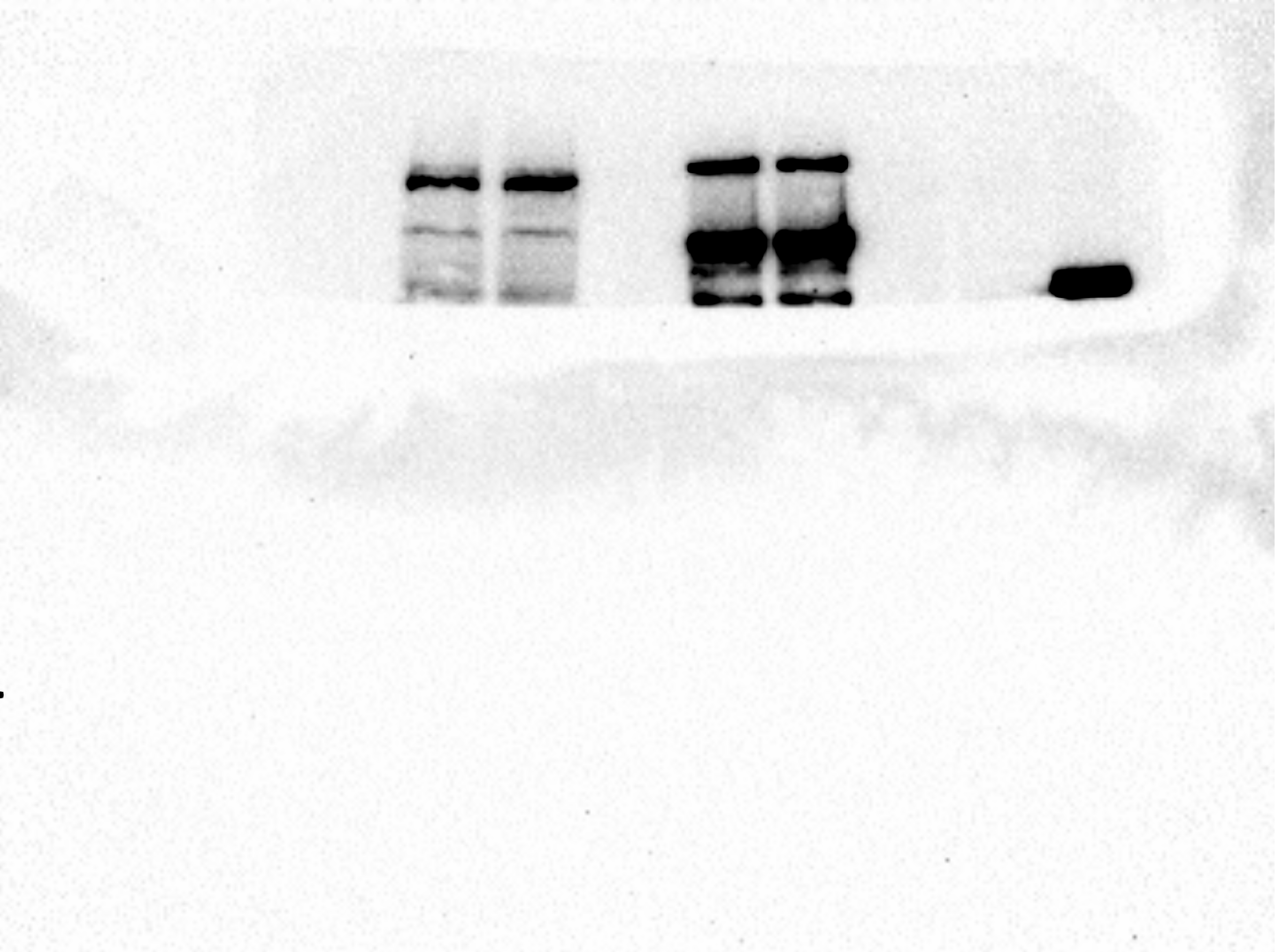

Supplement: Supplementary file 7 — Source data Fig. 3 [file 44319_2025_446_MOESM7_ESM.zip › Figure 3/3G/Western Blot YEATS2 IP/YEATS2 .tif]

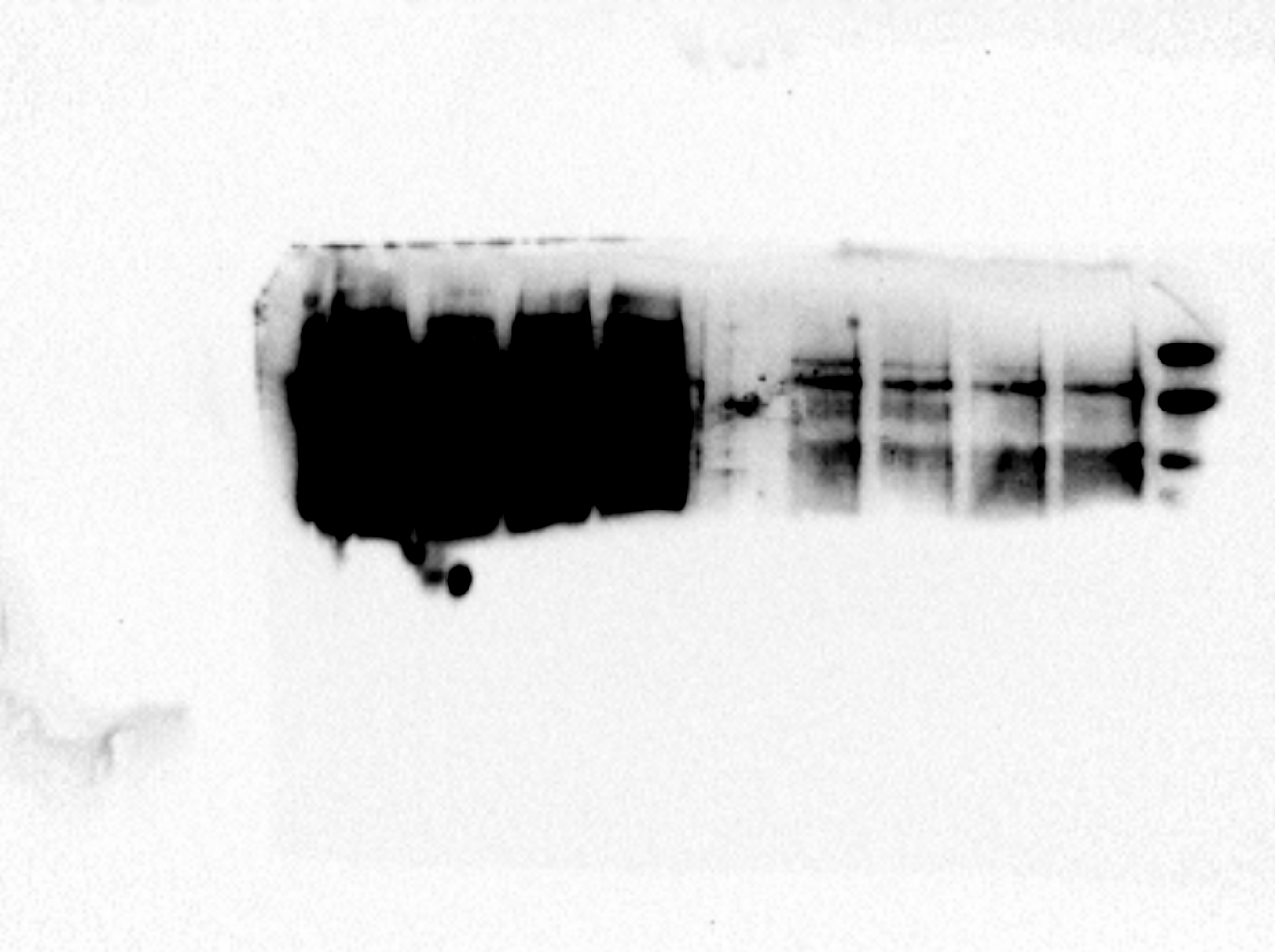

Supplement: Supplementary file 7 — Source data Fig. 3 [file 44319_2025_446_MOESM7_ESM.zip › Figure 3/3H/Western Blot 4G10/4G10.tif]

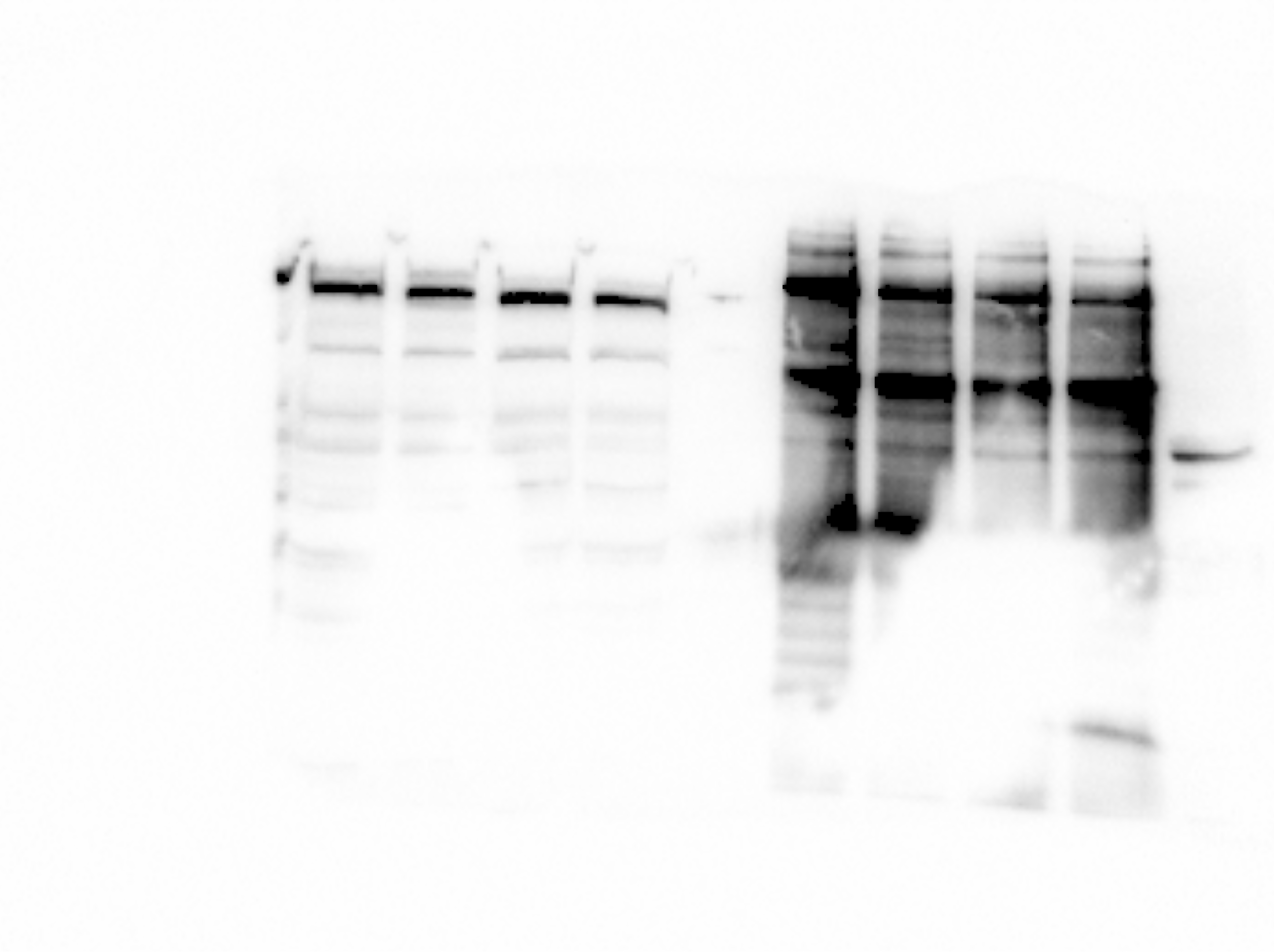

Supplement: Supplementary file 7 — Source data Fig. 3 [file 44319_2025_446_MOESM7_ESM.zip › Figure 3/3H/Western Blot YEATS2/YEATS2.tif]

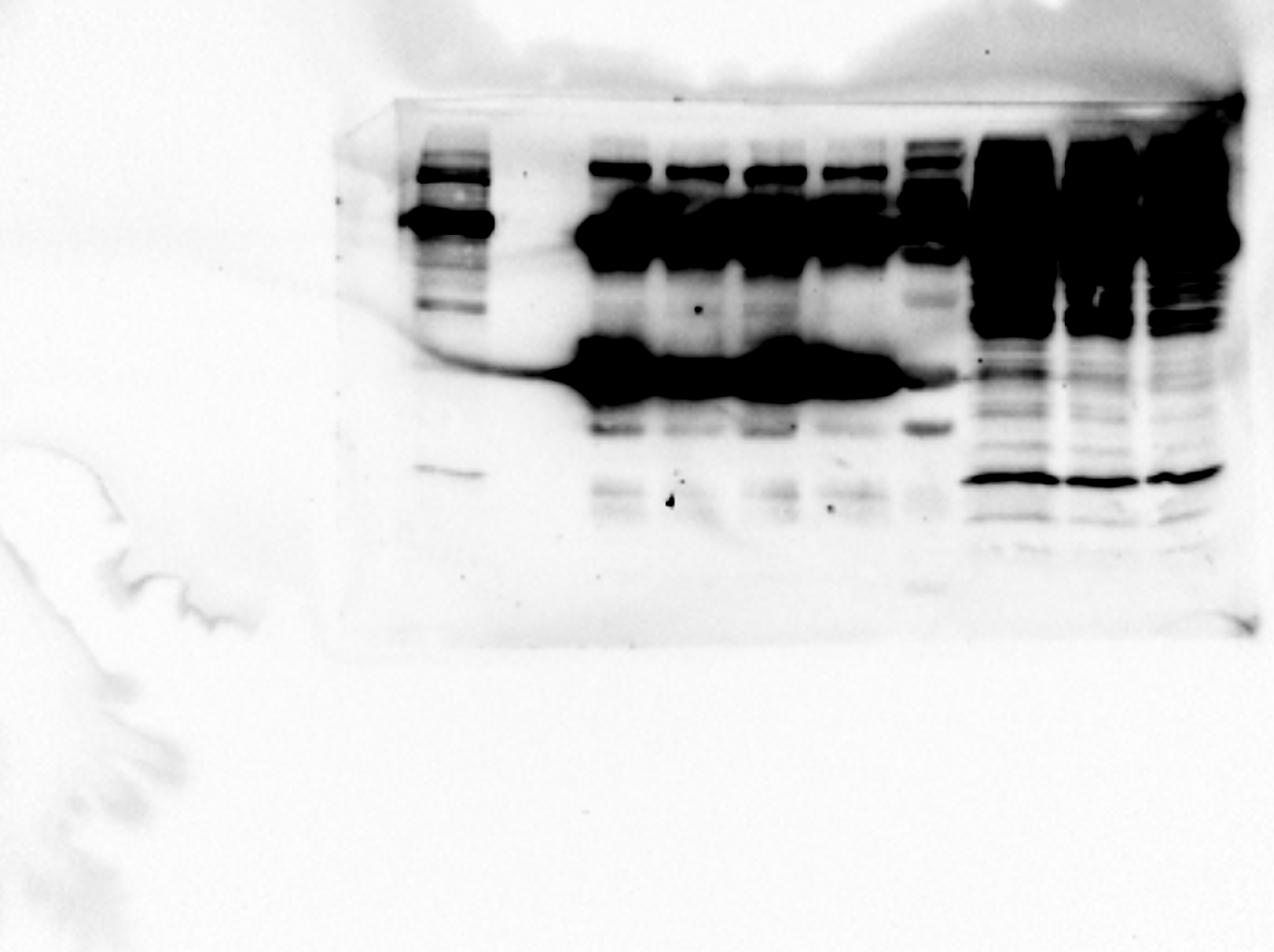

Supplement: Supplementary file 7 — Source data Fig. 3 [file 44319_2025_446_MOESM7_ESM.zip › Figure 3/3I/Western Blot 4G10/4G10 .tif]

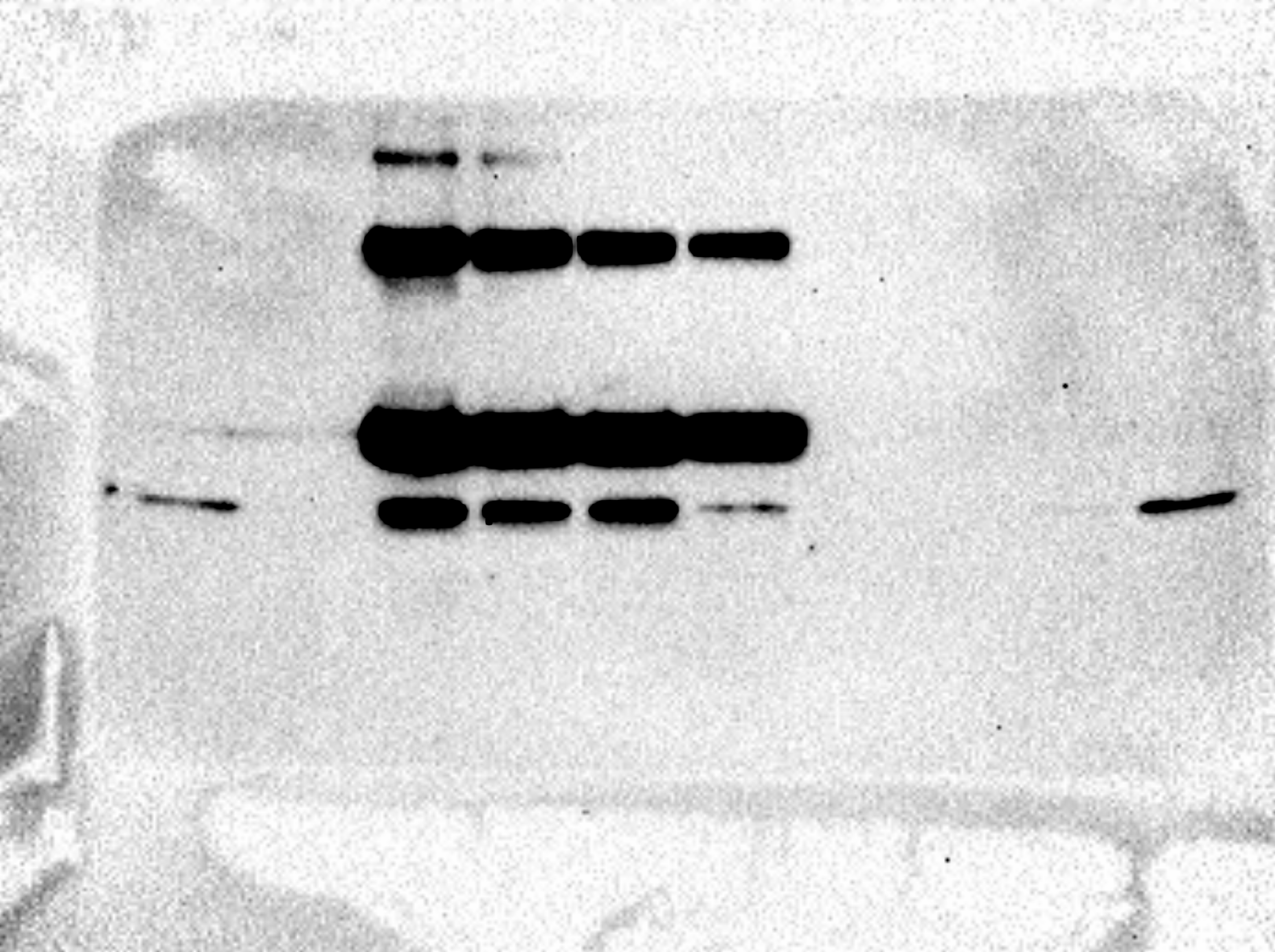

Supplement: Supplementary file 7 — Source data Fig. 3 [file 44319_2025_446_MOESM7_ESM.zip › Figure 3/3I/Western Blot Flag/FLAG.tif]

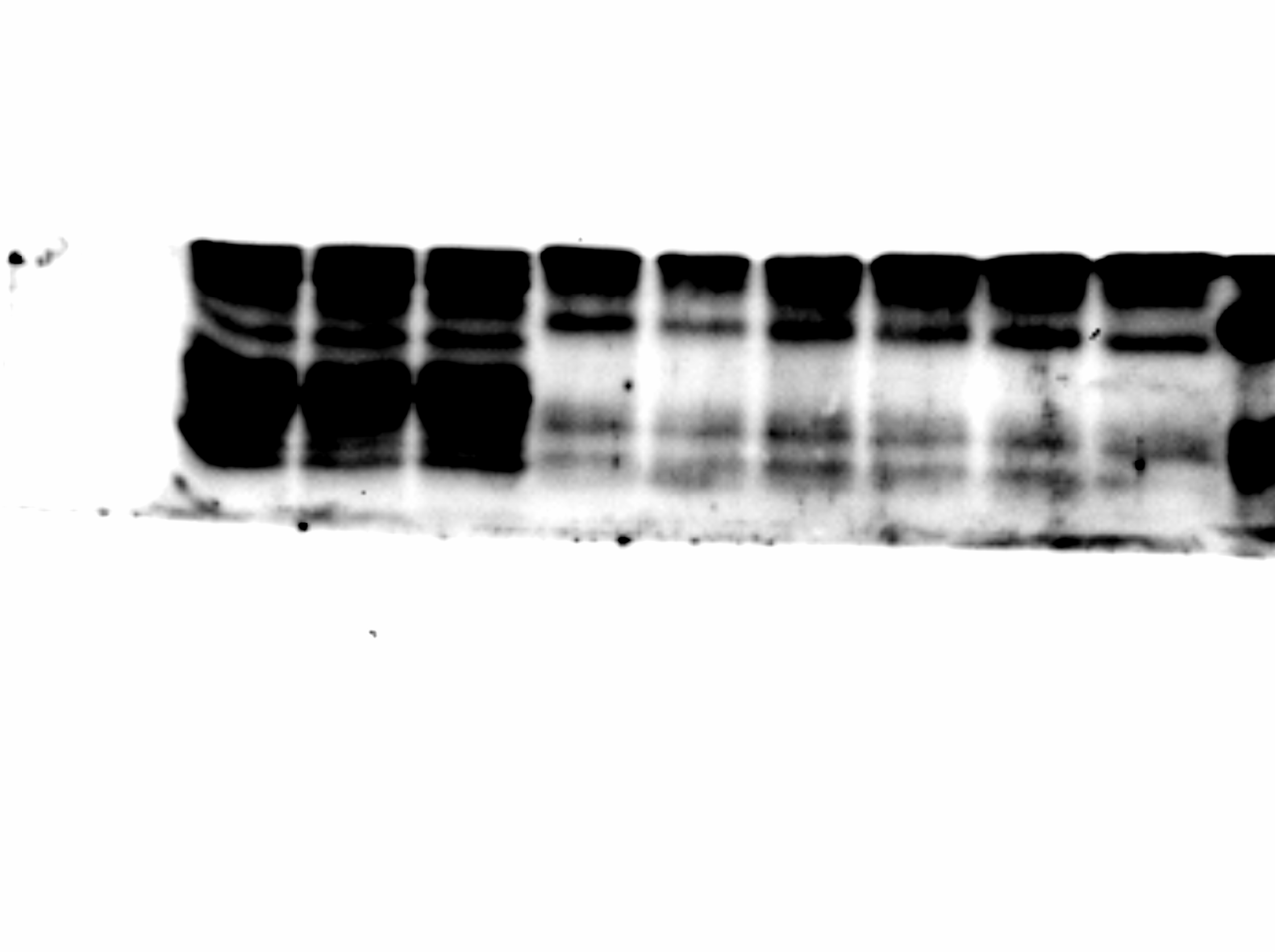

Supplement: Supplementary file 7 — Source data Fig. 3 [file 44319_2025_446_MOESM7_ESM.zip › Figure 3/3K/Western Blot 4G10/4G10.tif]

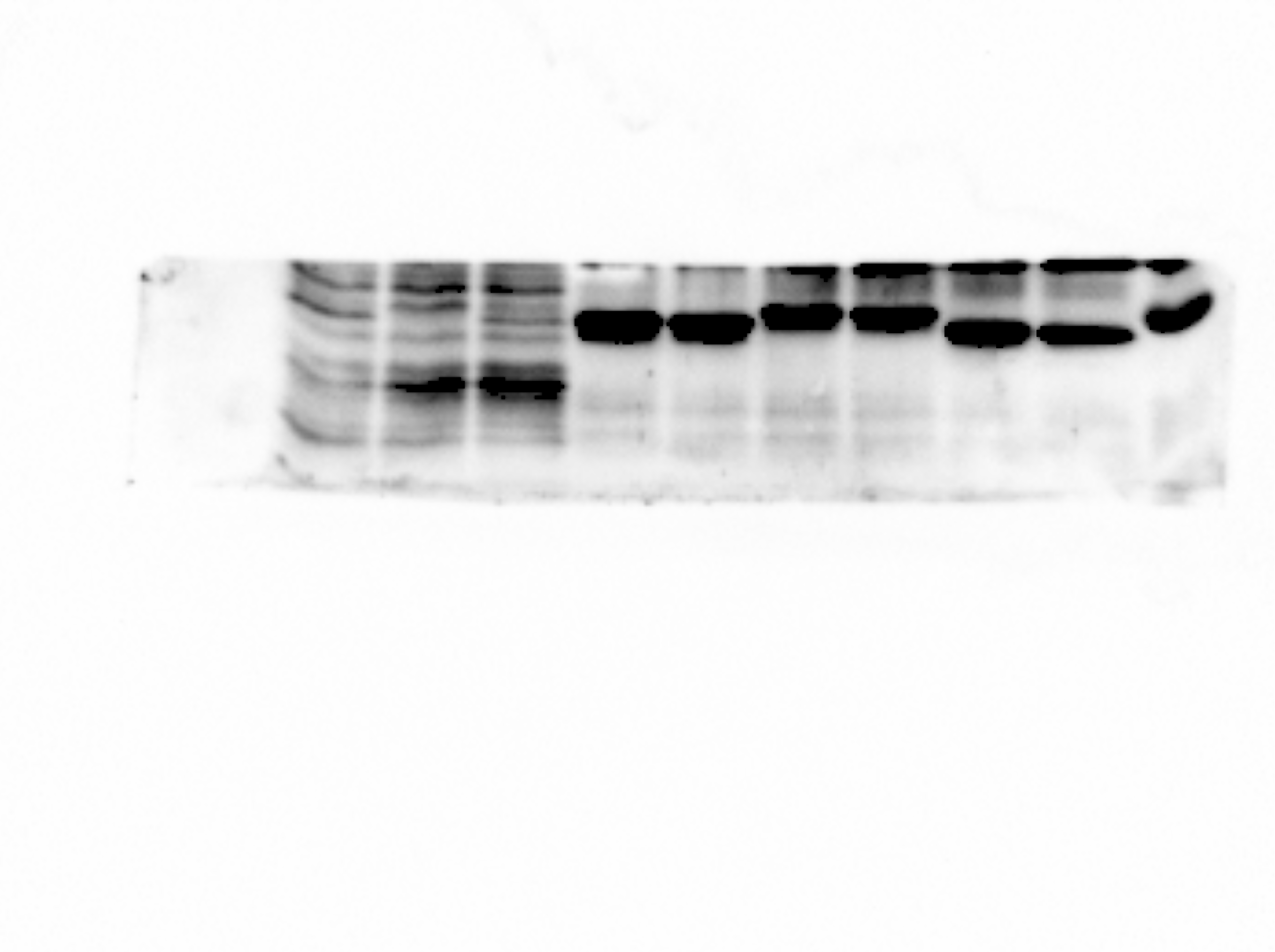

Supplement: Supplementary file 7 — Source data Fig. 3 [file 44319_2025_446_MOESM7_ESM.zip › Figure 3/3K/Western Blot Flag/FLAG FINAL.tif]

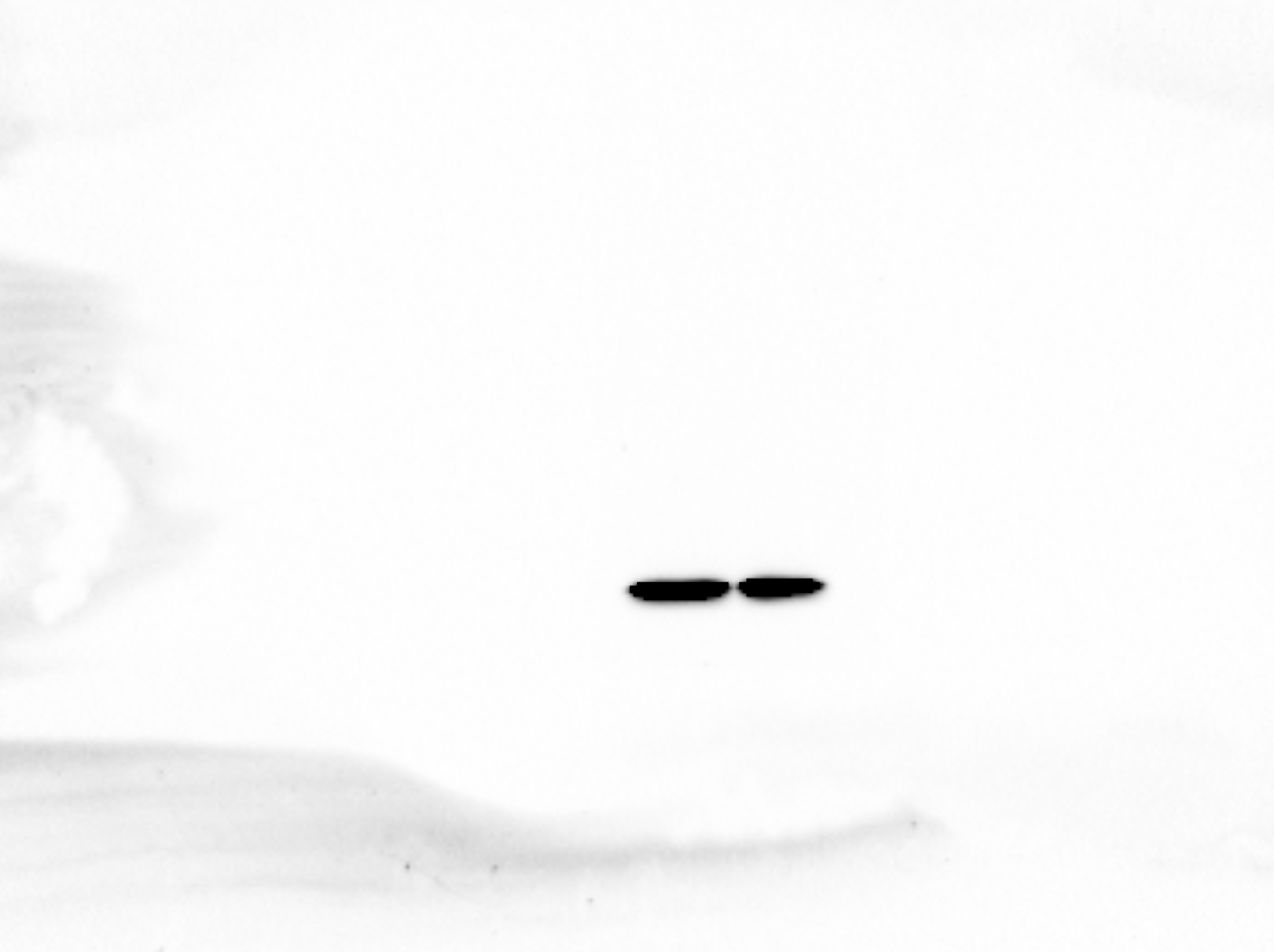

Supplement: Supplementary file 7 — Source data Fig. 3 [file 44319_2025_446_MOESM7_ESM.zip › Figure 3/3K/Western Blot Input Flag/FLAG INPUT.tif]

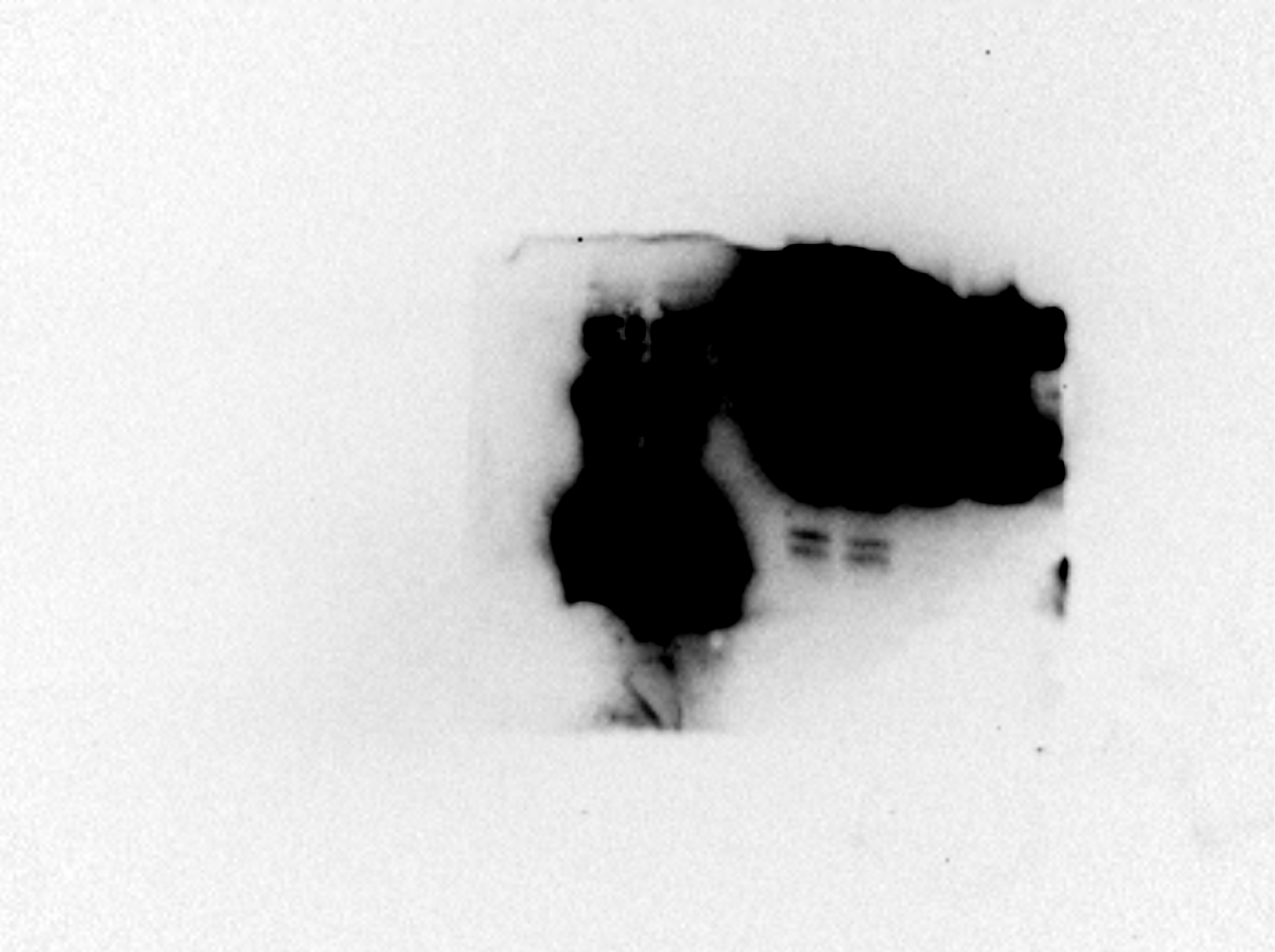

Supplement: Supplementary file 8 — Source data Fig. 4 [file 44319_2025_446_MOESM8_ESM.zip › Figure 4/4F/Lower Panel/Western Blot H3 IP/H3_IP.tif]

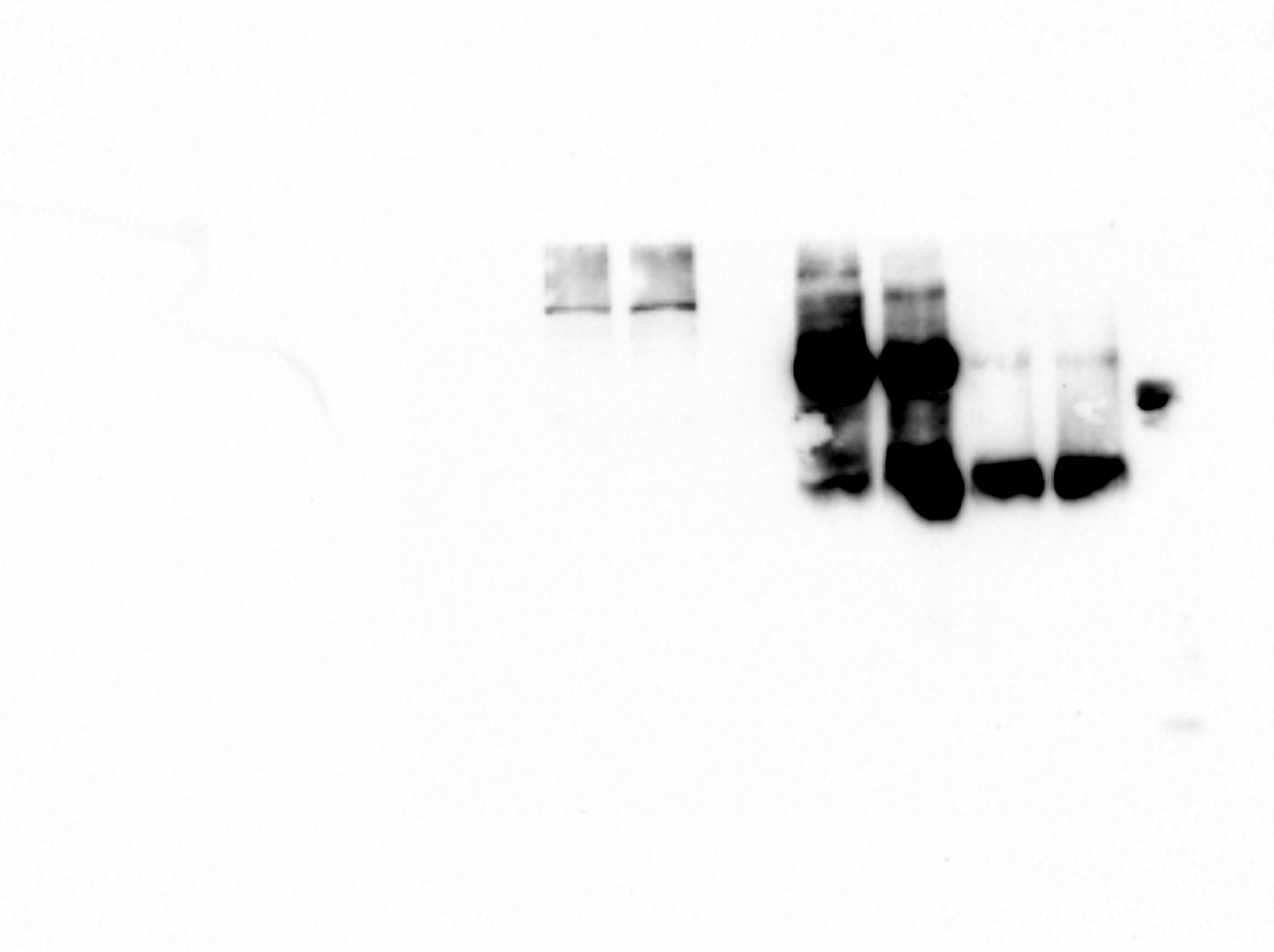

Supplement: Supplementary file 8 — Source data Fig. 4 [file 44319_2025_446_MOESM8_ESM.zip › Figure 4/4F/Lower Panel/Western Blot YEATS2 IP/Yeats2_IP_Final_.tif]

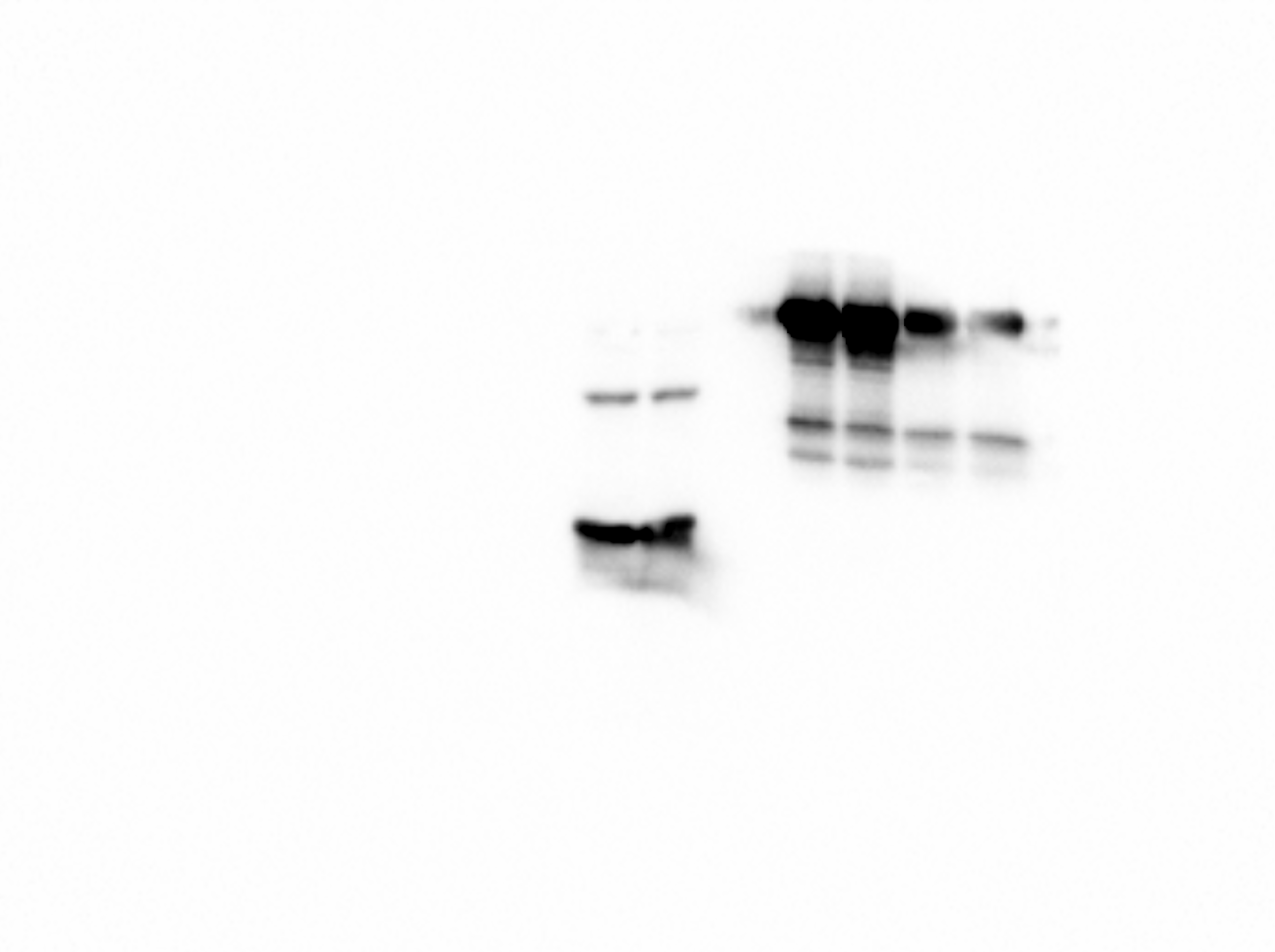

Supplement: Supplementary file 8 — Source data Fig. 4 [file 44319_2025_446_MOESM8_ESM.zip › Figure 4/4F/Lower Panel/Western Input H3/H3_Input_Final.tif]

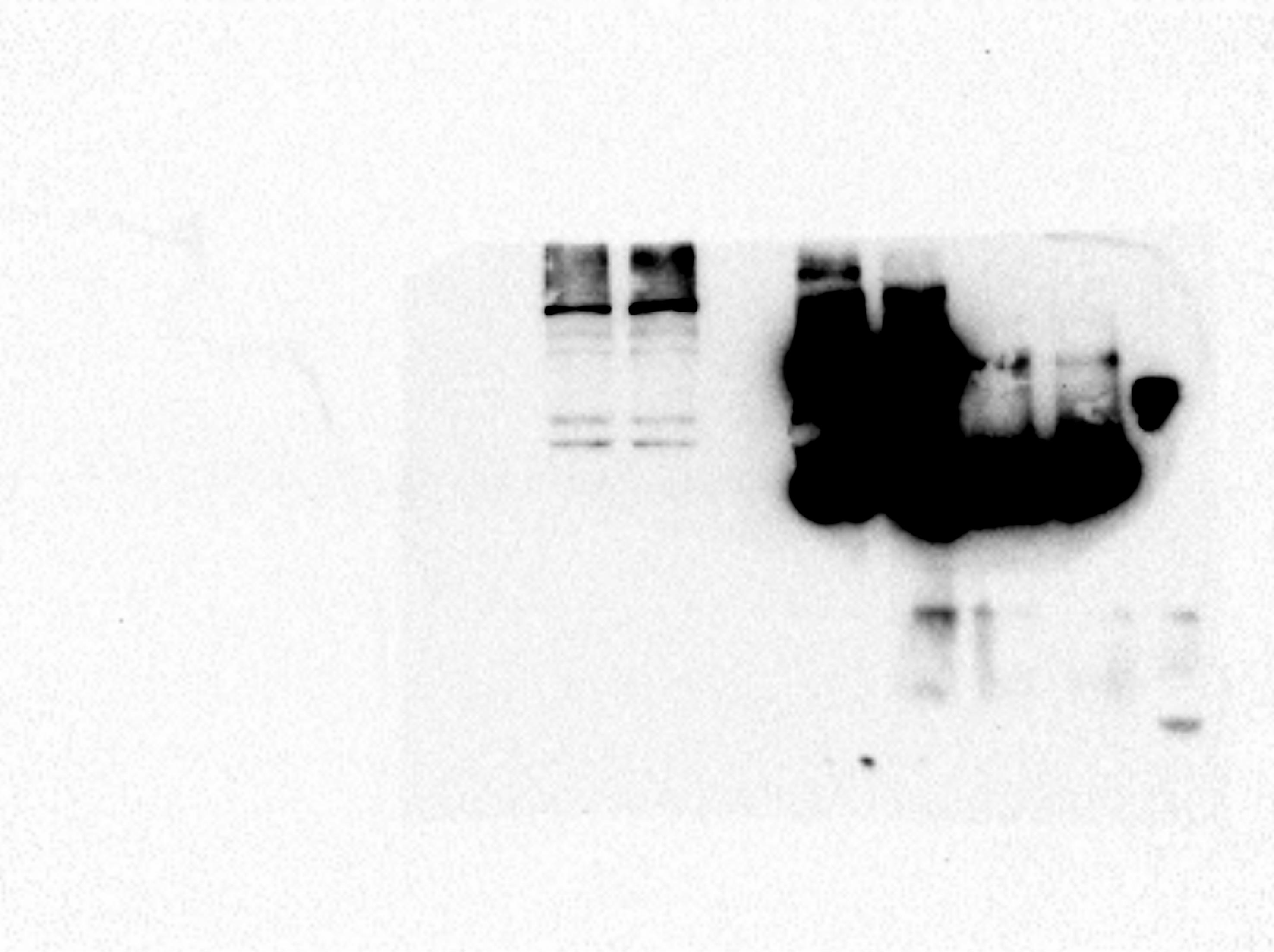

Supplement: Supplementary file 8 — Source data Fig. 4 [file 44319_2025_446_MOESM8_ESM.zip › Figure 4/4F/Lower Panel/Western Input YEATS2/Yeats2_Input_Final.tif]

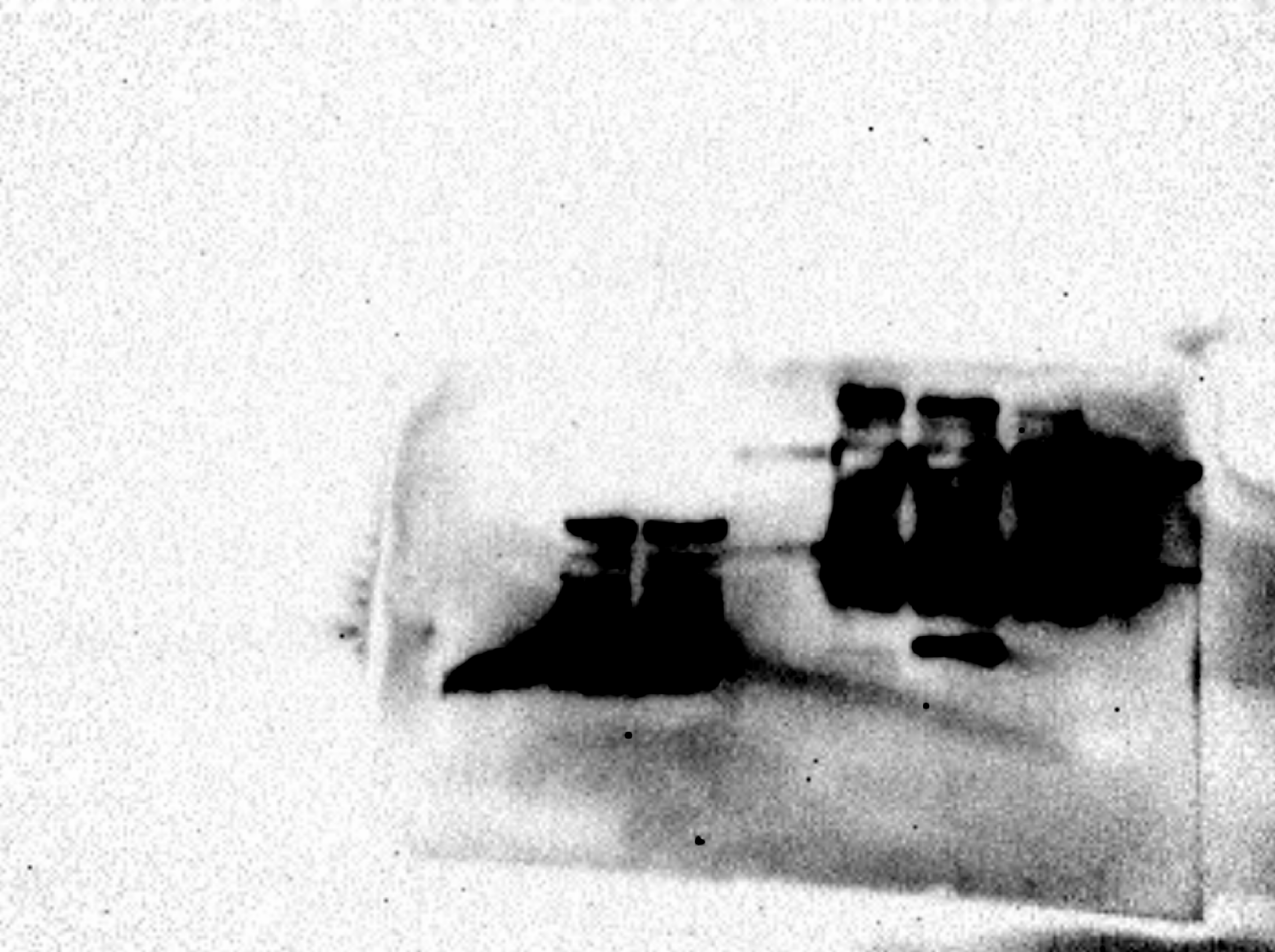

Supplement: Supplementary file 8 — Source data Fig. 4 [file 44319_2025_446_MOESM8_ESM.zip › Figure 4/4F/Middle Panel/Western Blot H3 IP/H3_IP_High expo_120 sec.tif]

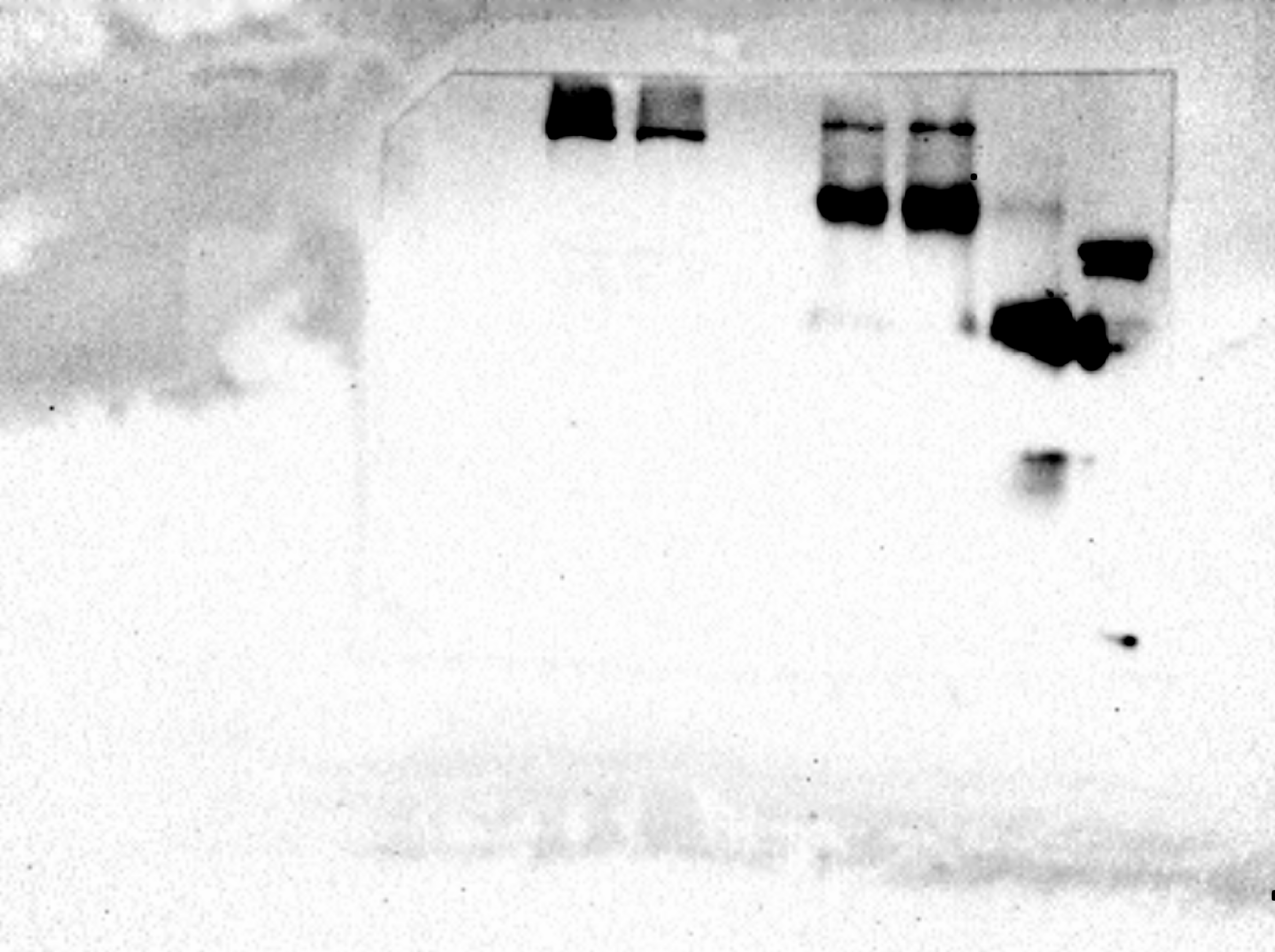

Supplement: Supplementary file 8 — Source data Fig. 4 [file 44319_2025_446_MOESM8_ESM.zip › Figure 4/4F/Middle Panel/Western Blot YEATS2 IP/YEATS2_120 sec_IP_Final.tif]

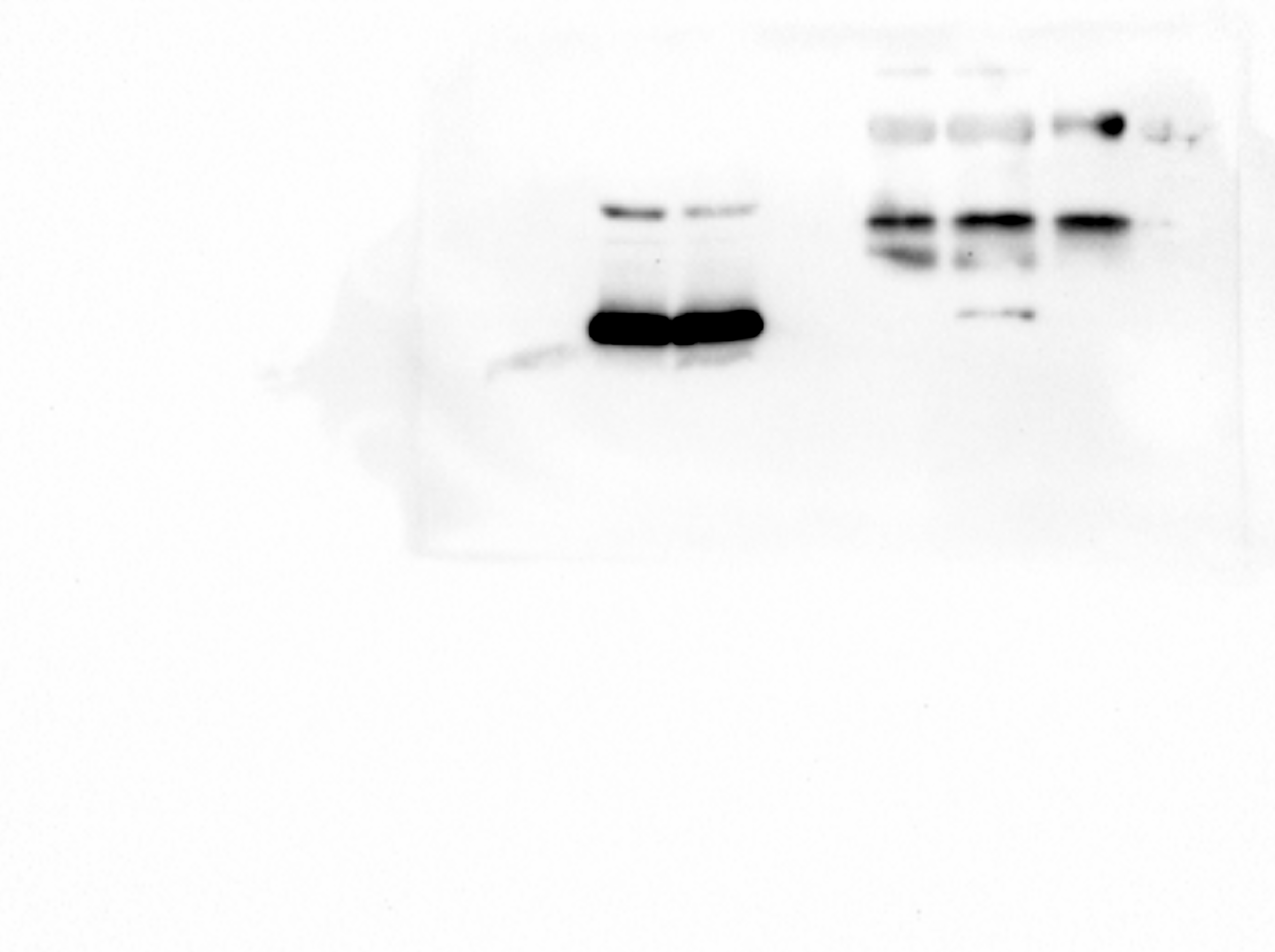

Supplement: Supplementary file 8 — Source data Fig. 4 [file 44319_2025_446_MOESM8_ESM.zip › Figure 4/4F/Middle Panel/Western Input H3/60 SEC H3_iNPUT_fINAL.tif]

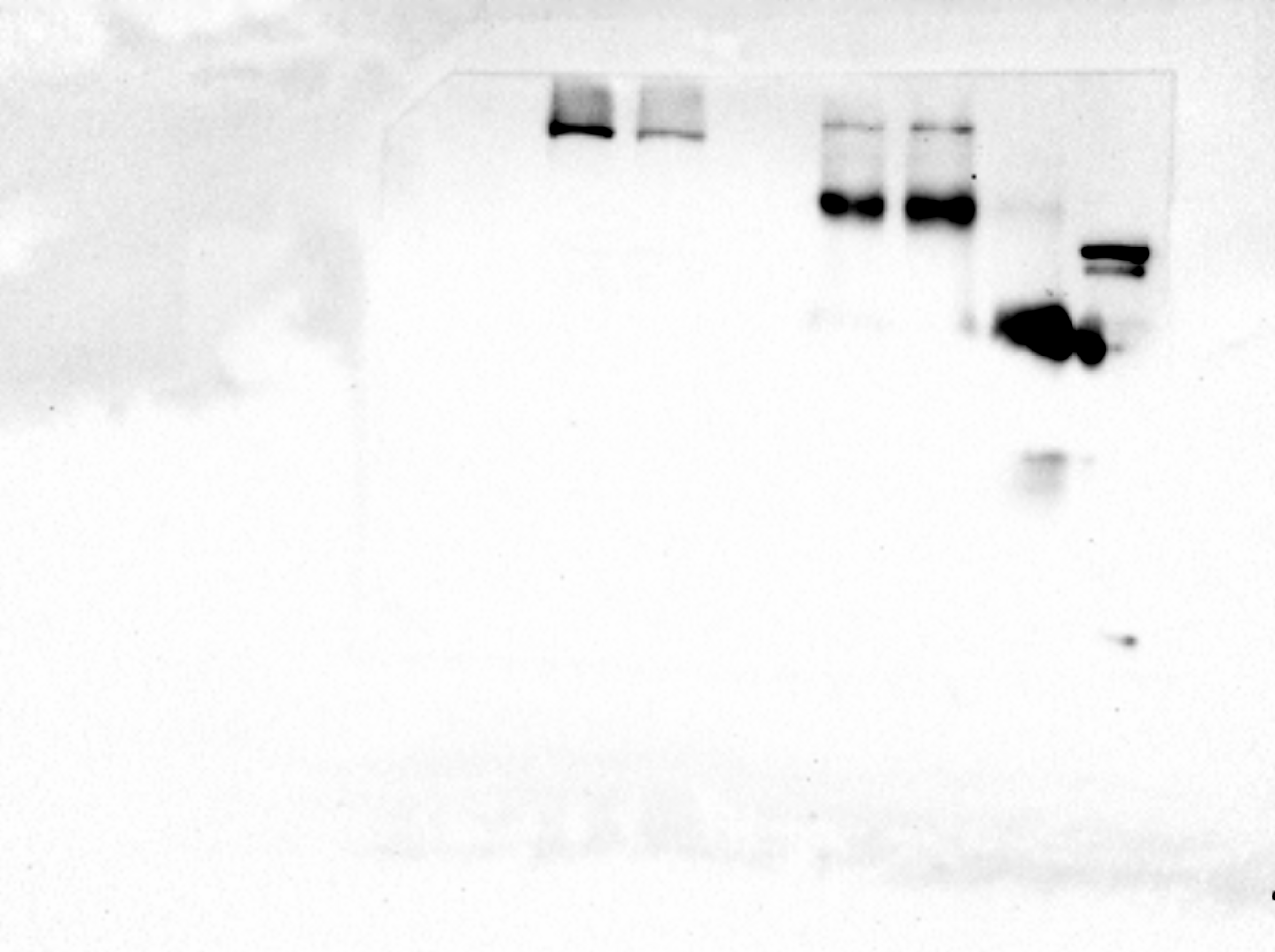

Supplement: Supplementary file 8 — Source data Fig. 4 [file 44319_2025_446_MOESM8_ESM.zip › Figure 4/4F/Middle Panel/Western Input YEATS2/YEATS2_120 sec_Input_Final.tif]

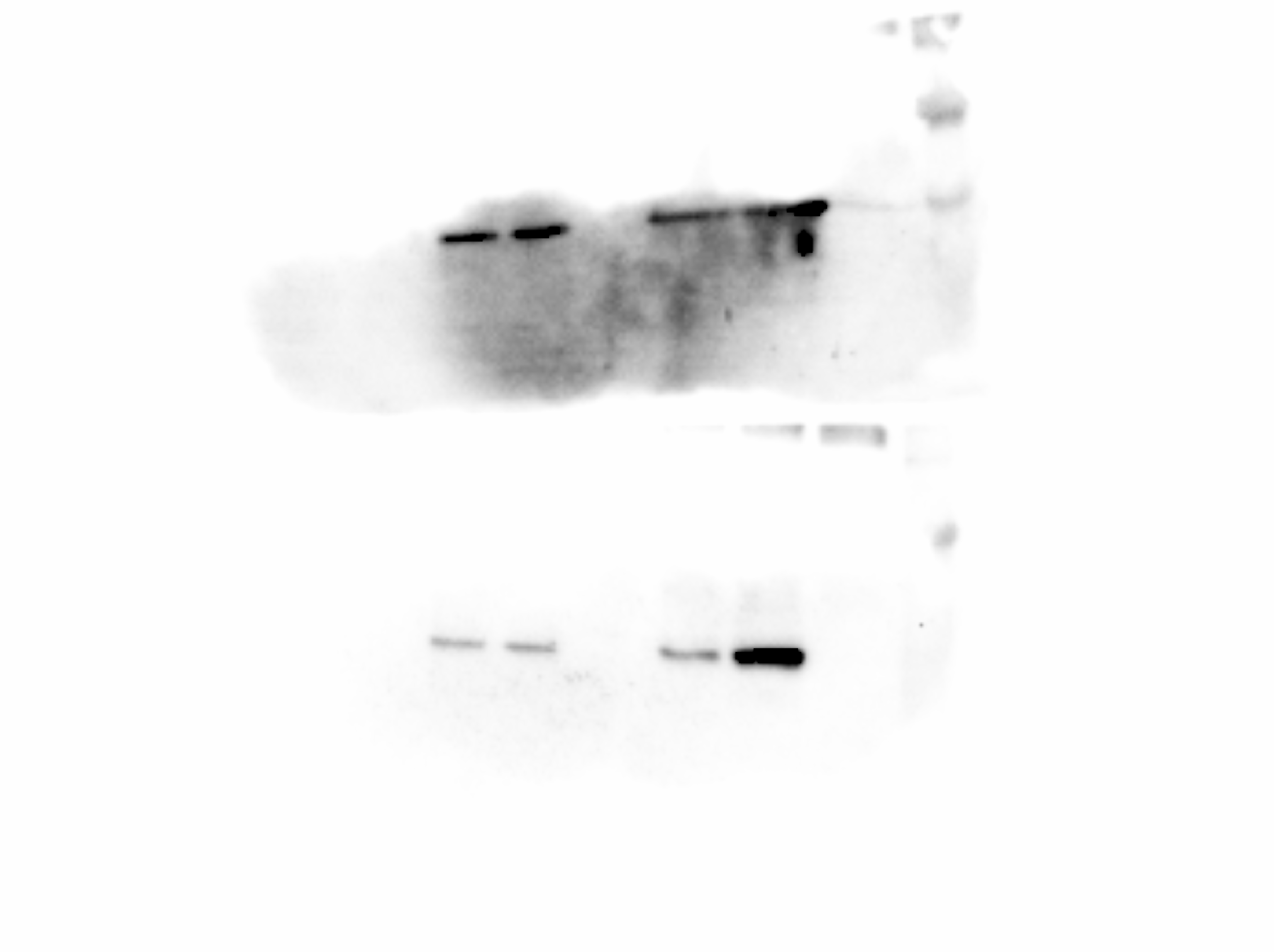

Supplement: Supplementary file 8 — Source data Fig. 4 [file 44319_2025_446_MOESM8_ESM.zip › Figure 4/4F/Upper Panel/Western Blot H3/Histone H3.tif]

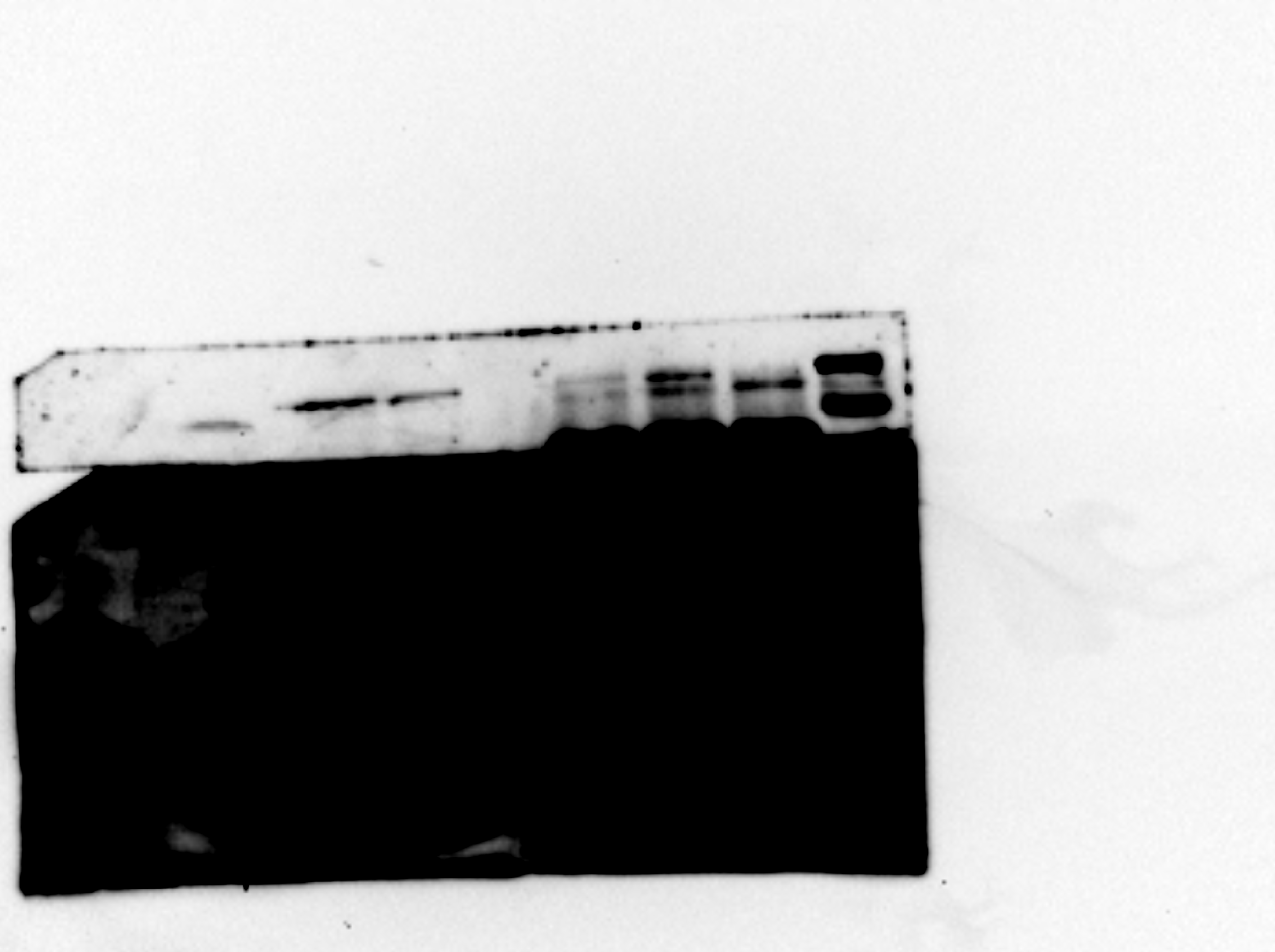

Supplement: Supplementary file 8 — Source data Fig. 4 [file 44319_2025_446_MOESM8_ESM.zip › Figure 4/4F/Upper Panel/Western Blot YEATS2/Yeats2_final.tif]

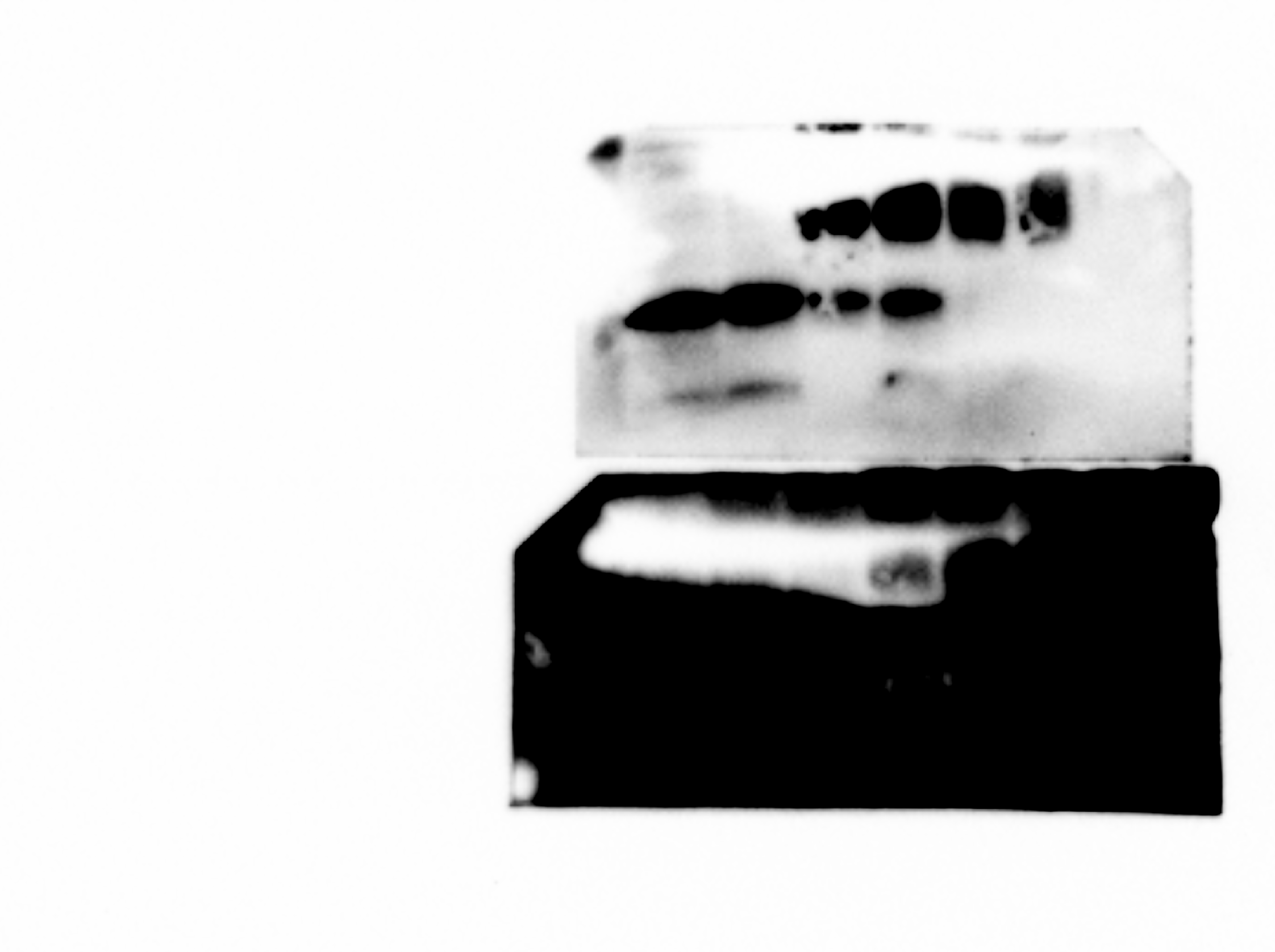

Supplement: Supplementary file 8 — Source data Fig. 4 [file 44319_2025_446_MOESM8_ESM.zip › Figure 4/4G/Lower Panel/Western Blot H3/H3_IP_Final.tif]

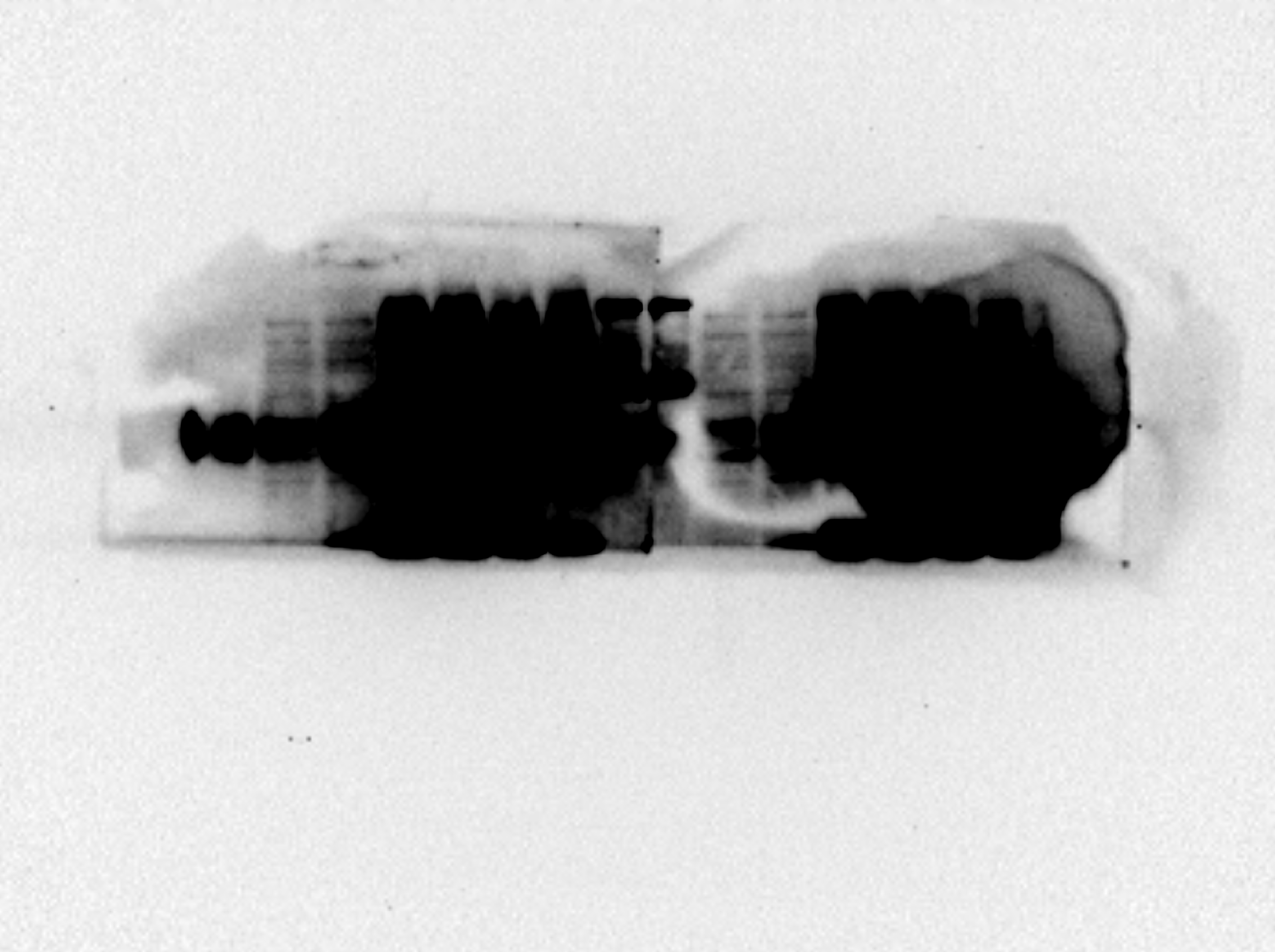

Supplement: Supplementary file 8 — Source data Fig. 4 [file 44319_2025_446_MOESM8_ESM.zip › Figure 4/4G/Lower Panel/Western Blot Input YEATS2/Yeats2_Input.tif]

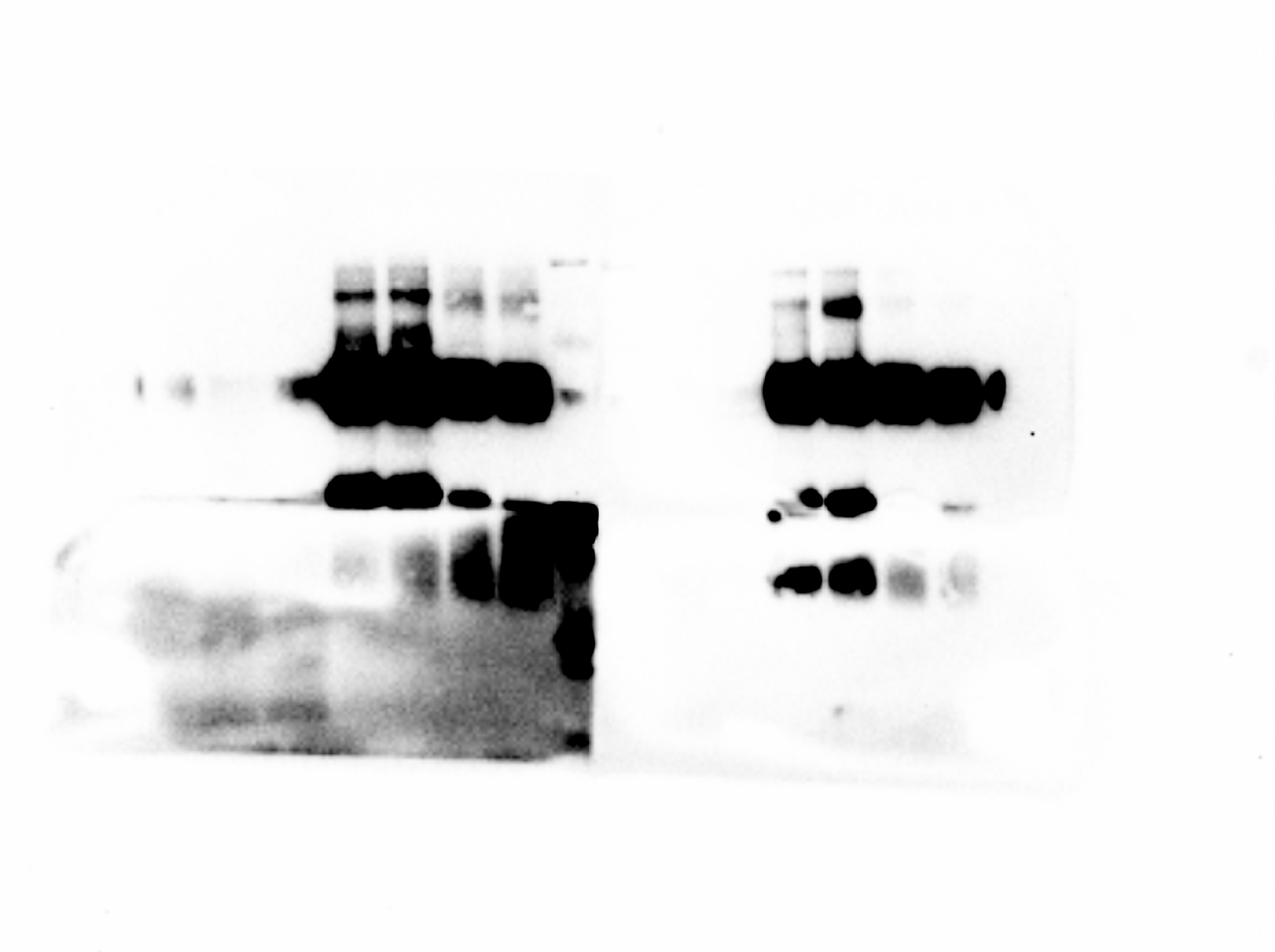

Supplement: Supplementary file 8 — Source data Fig. 4 [file 44319_2025_446_MOESM8_ESM.zip › Figure 4/4G/Lower Panel/Western Blot YEATS2 IP/Yeats2_IP.tif]

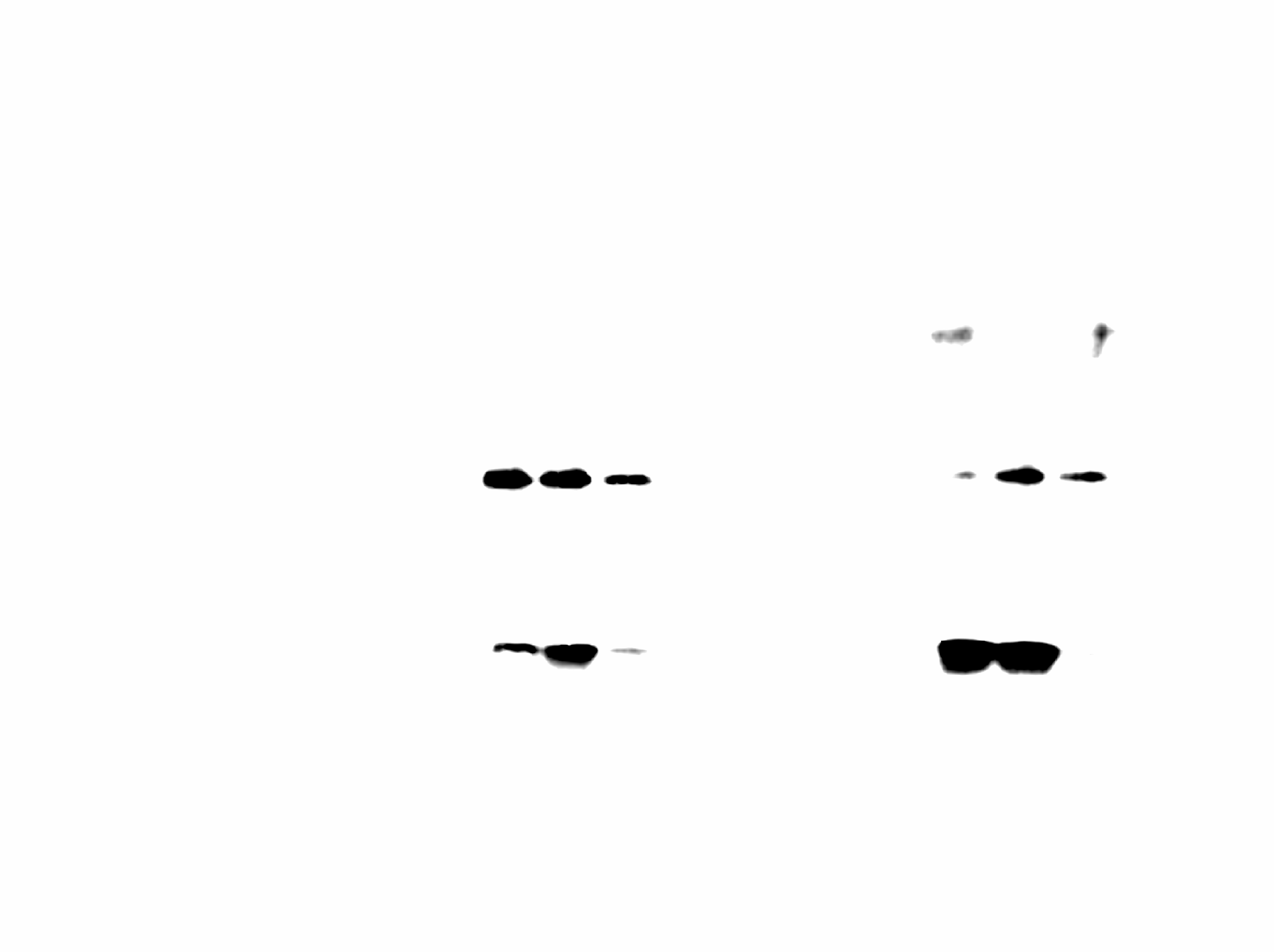

Supplement: Supplementary file 8 — Source data Fig. 4 [file 44319_2025_446_MOESM8_ESM.zip › Figure 4/4G/Upper Panel/Western Blot H3 IP/H3_IP.tif]

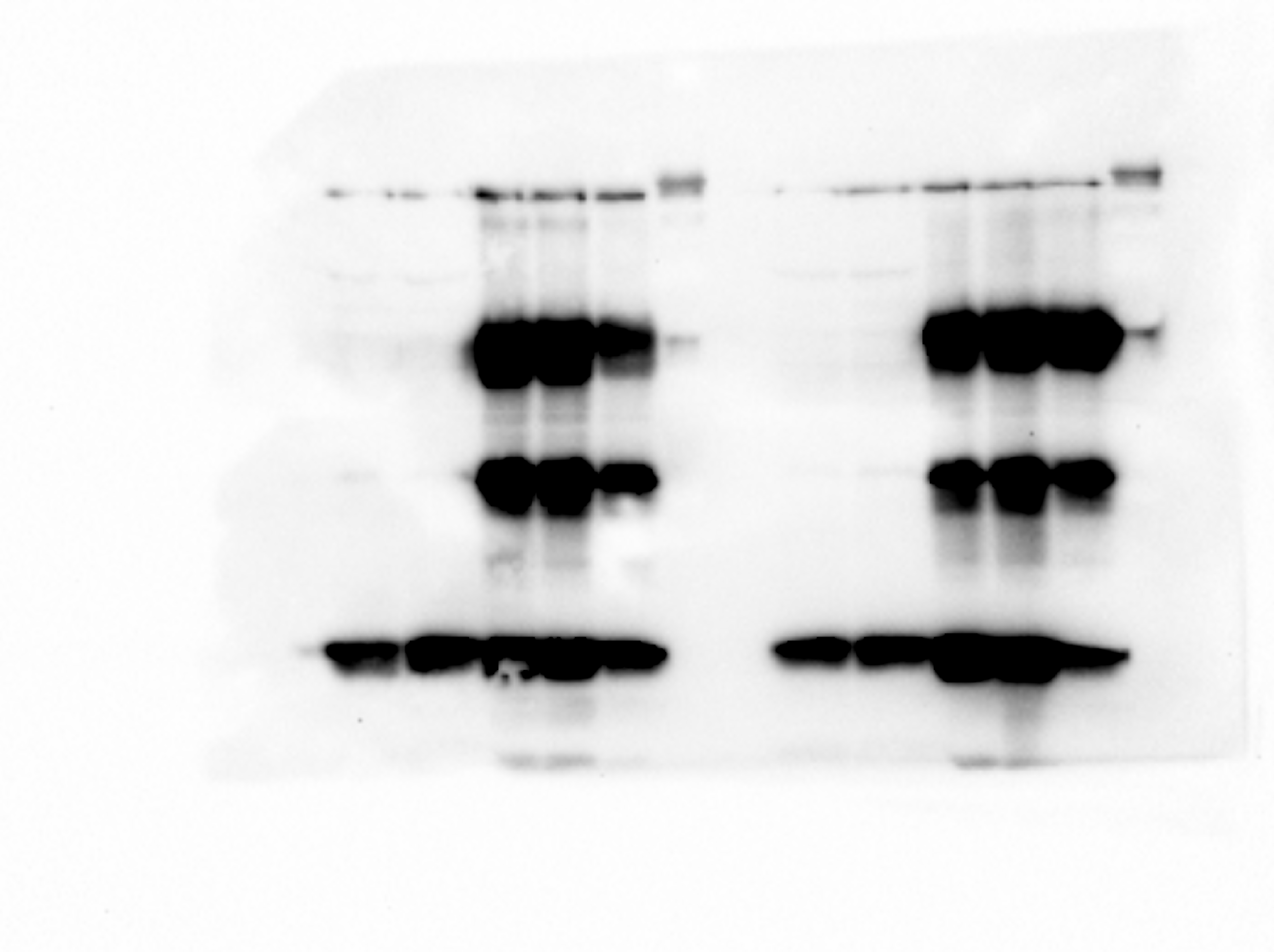

Supplement: Supplementary file 8 — Source data Fig. 4 [file 44319_2025_446_MOESM8_ESM.zip › Figure 4/4G/Upper Panel/Western Blot Input H3/H3_Input_Final.tif]

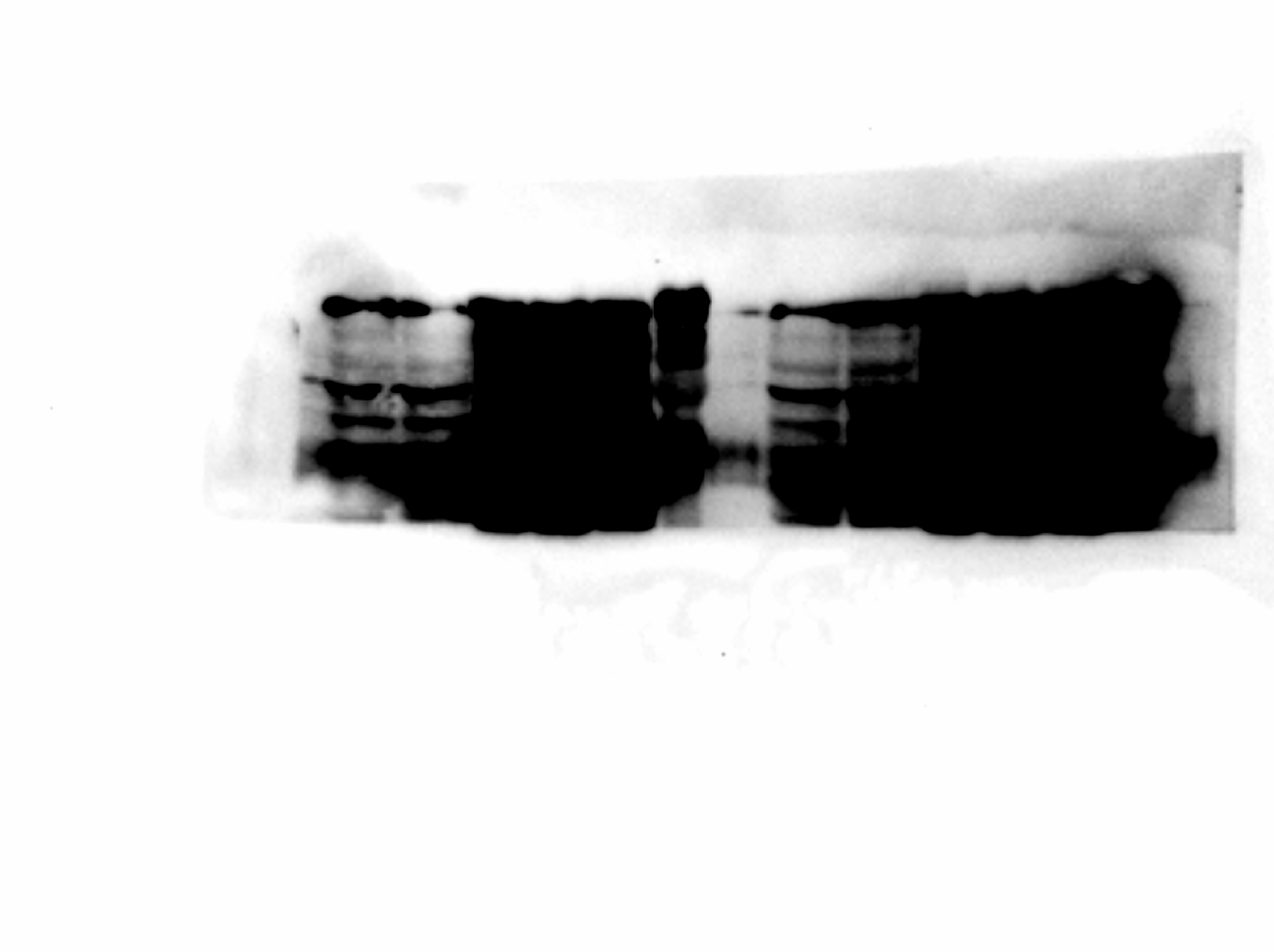

Supplement: Supplementary file 8 — Source data Fig. 4 [file 44319_2025_446_MOESM8_ESM.zip › Figure 4/4G/Upper Panel/Western Blot Input YEATS2/Yeats2_input.tif]

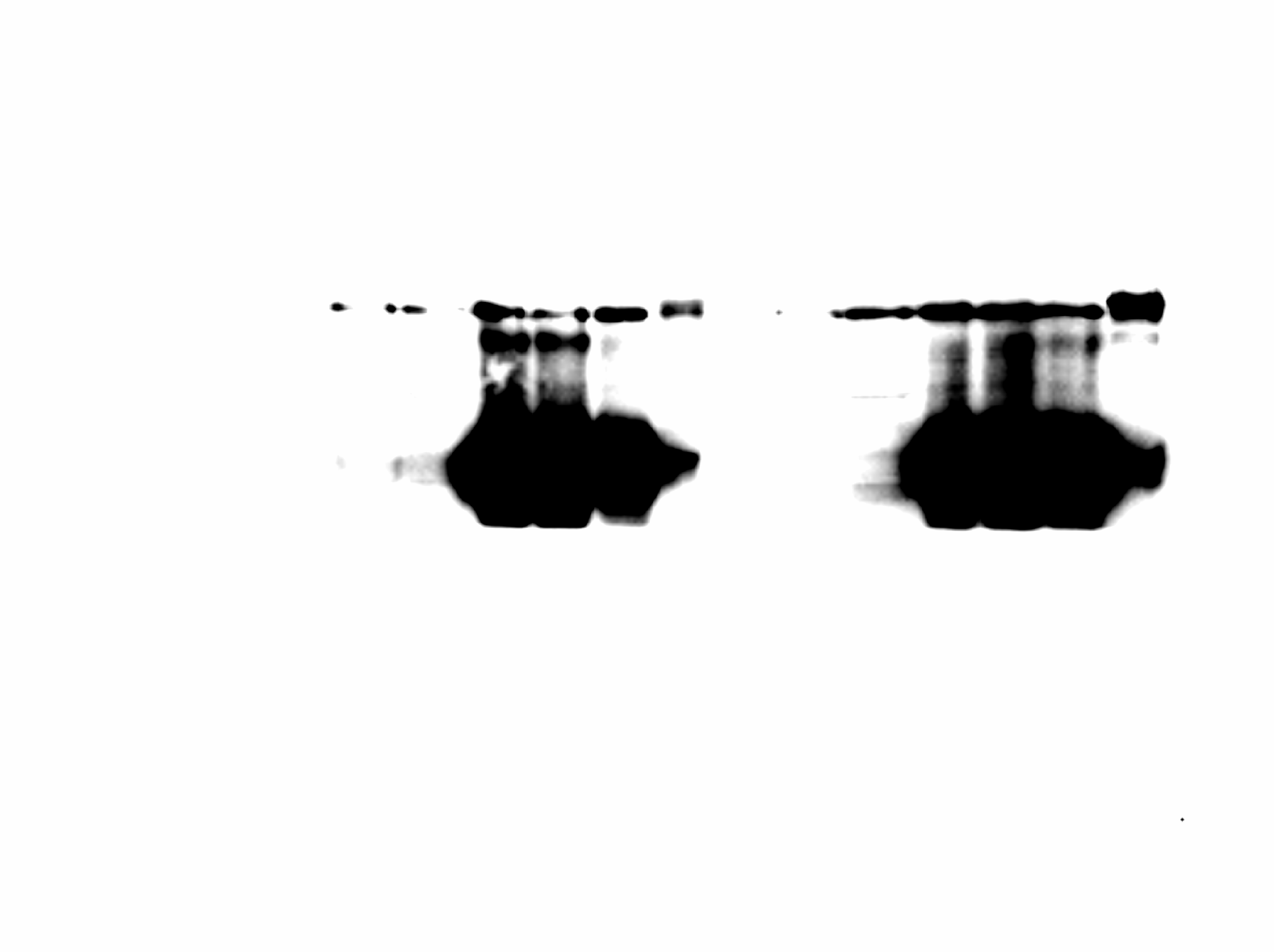

Supplement: Supplementary file 8 — Source data Fig. 4 [file 44319_2025_446_MOESM8_ESM.zip › Figure 4/4G/Upper Panel/Western Blot YEATS2 IP/Yeats2_IP.tif]
