# Supplementary material for: Dynamic interaction of MYC enhancer RNA with YEATS2 protein regulates MYC gene transcription in pancreatic cancer
Source: EMBO Rep. 2025 Apr 11;26(10):2519–44. doi: 10.1038/s44319-025-00446-0 (PMC12117045; doi:10.1038/s44319-025-00446-0)
Supplement: Supplementary file 7 — Source data Fig. 3 [file 44319_2025_446_MOESM7_ESM.zip › Figure 3/3D/README.docx]

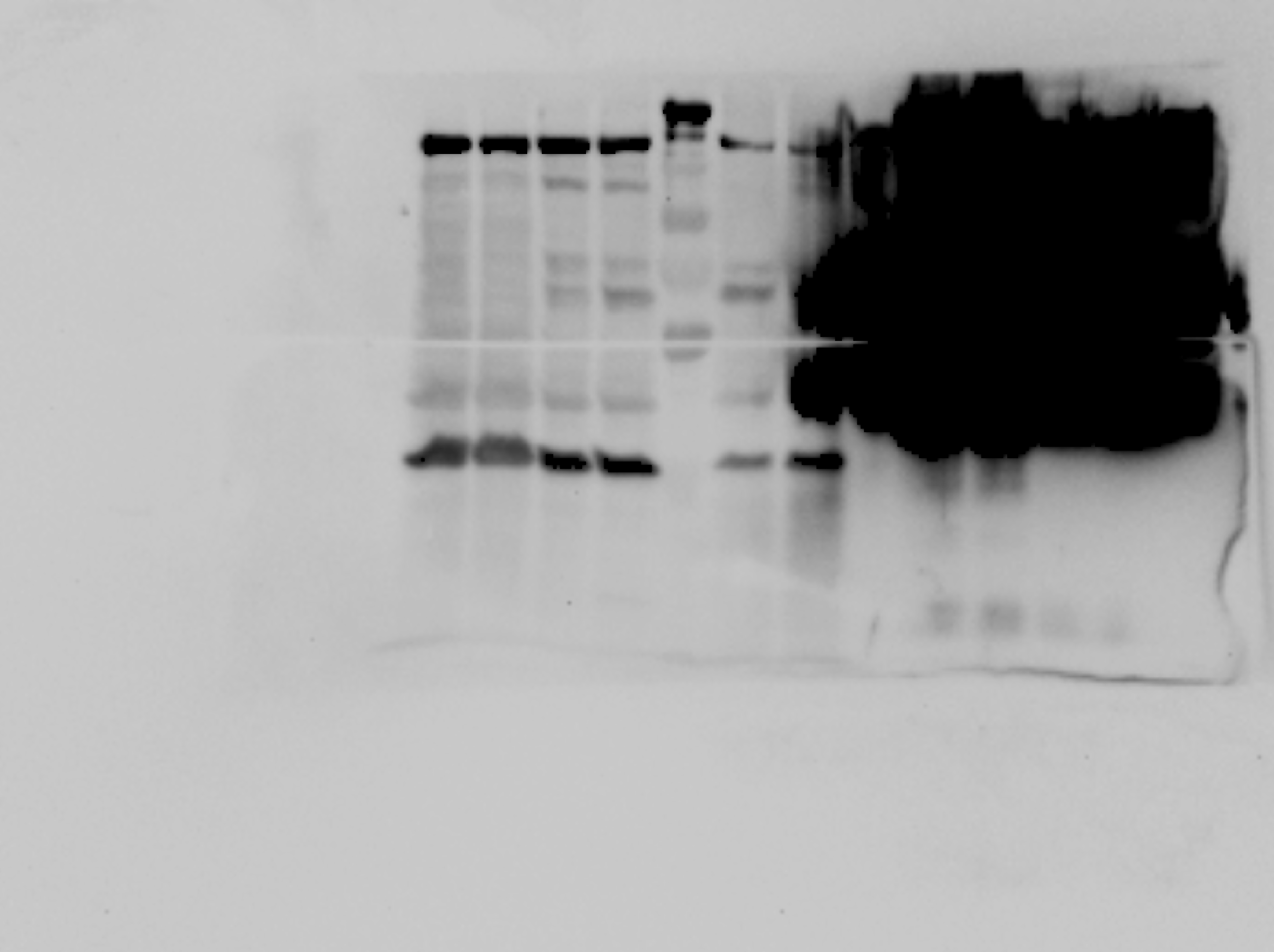


**150kDa**

**37kDa**

**Total YEATS2 in MIAPaCa-2 and HPNE**

**Total GAPDH in MIAPaCa-2 and HPNE**

**TNF-α (h)**

**0**

**24**

**YEATS2**

**GAPDH**

**MiaPaCa2**

**HPNE**

**24**

**0**

**Fig 3D**
