# Supplementary material for: Dynamic interaction of MYC enhancer RNA with YEATS2 protein regulates MYC gene transcription in pancreatic cancer
Source: EMBO Rep. 2025 Apr 11;26(10):2519–44. doi: 10.1038/s44319-025-00446-0 (PMC12117045; doi:10.1038/s44319-025-00446-0)
Supplement: Supplementary file 7 — Source data Fig. 3 [file 44319_2025_446_MOESM7_ESM.zip › Figure 3/3E/README.docx]

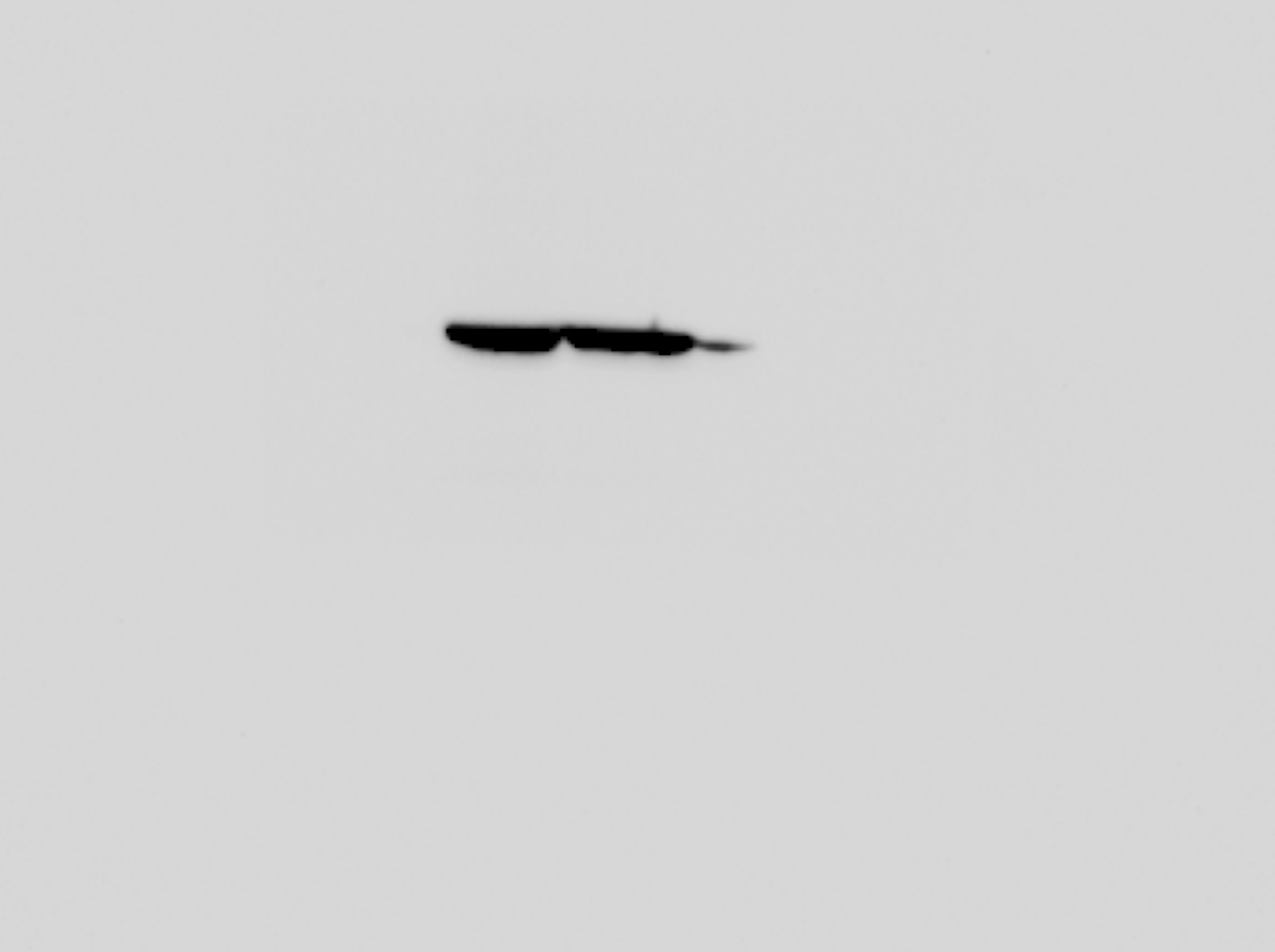

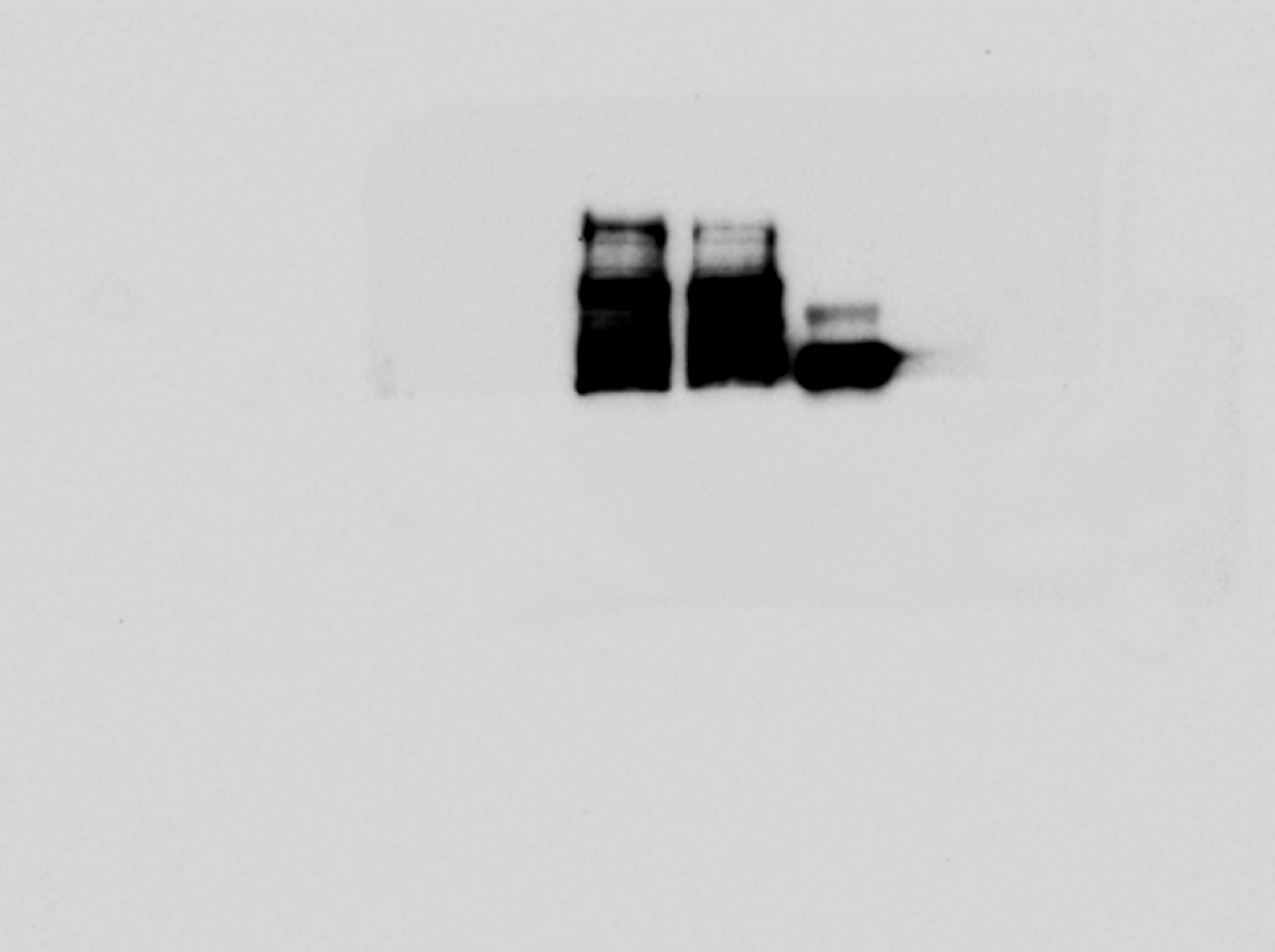


**150kDa**

**YEATS2 Knock down in MIAPaCa-2**

**sh scramble**

**sh YEATS2 #1**

**YEATS2**

**37kDa**

**GAPDH protein levels in MIAPaCa-2 cells following YEATS2 knockdown**

**GAPDH**

**sh scramble**

**sh YEATS2 #1**

**Fig 3E**
