# Supplementary material for: Dynamic interaction of MYC enhancer RNA with YEATS2 protein regulates MYC gene transcription in pancreatic cancer
Source: EMBO Rep. 2025 Apr 11;26(10):2519–44. doi: 10.1038/s44319-025-00446-0 (PMC12117045; doi:10.1038/s44319-025-00446-0)
Supplement: Supplementary file 7 — Source data Fig. 3 [file 44319_2025_446_MOESM7_ESM.zip › Figure 3/3H/README.docx]

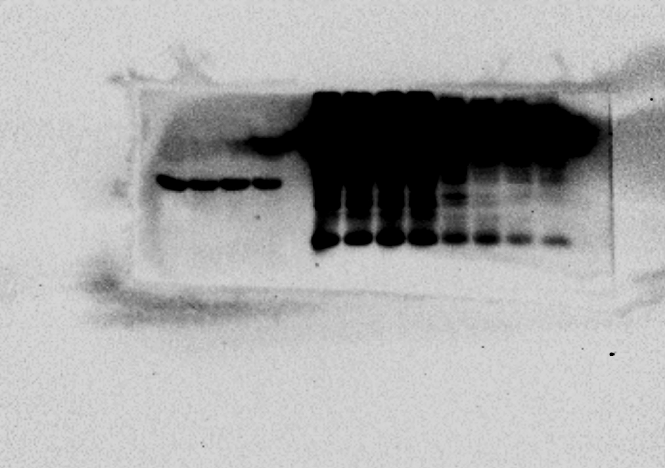

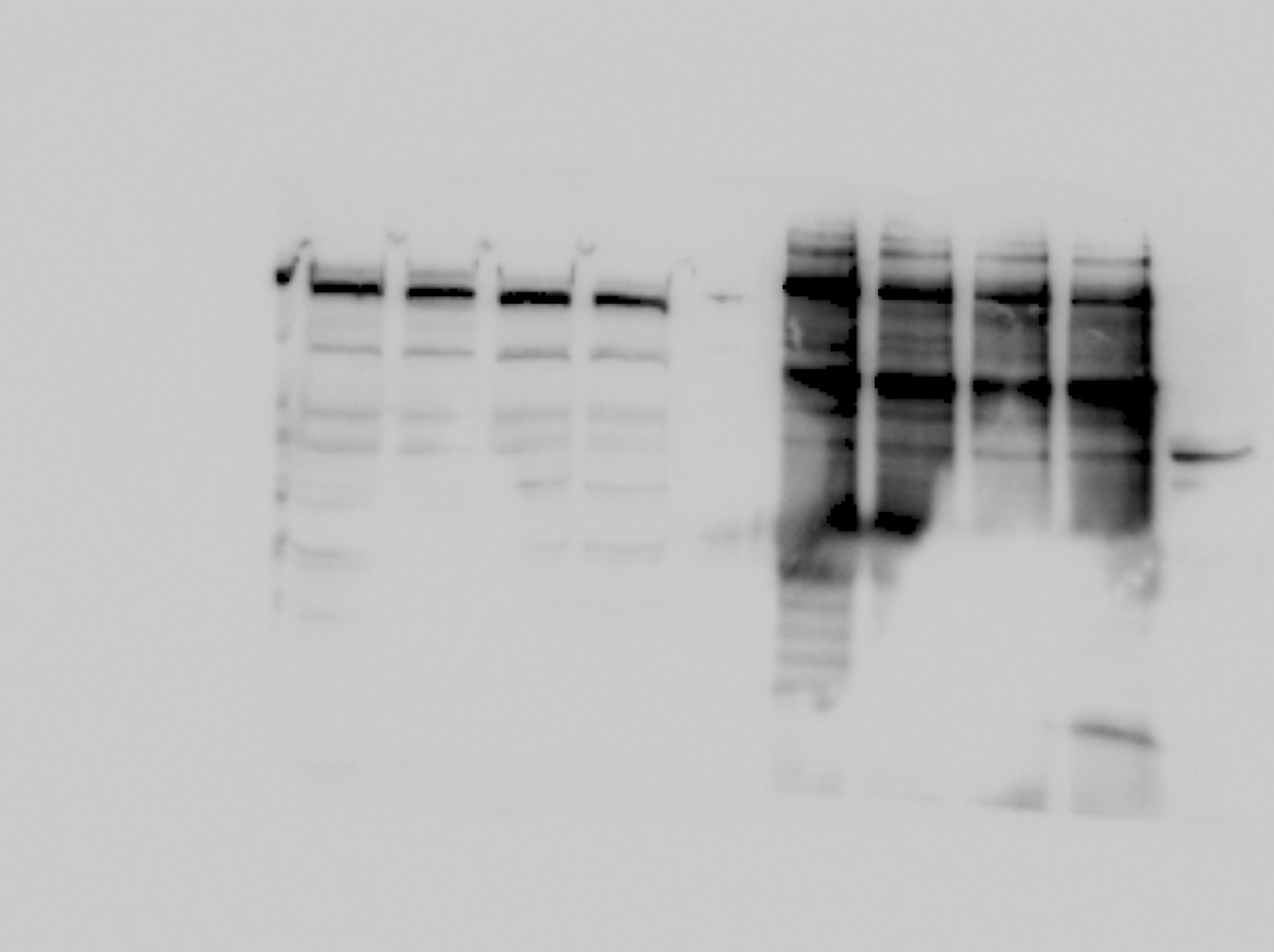


**150kDa**

**YEATS2 in Phosphatase assay in HPNE**


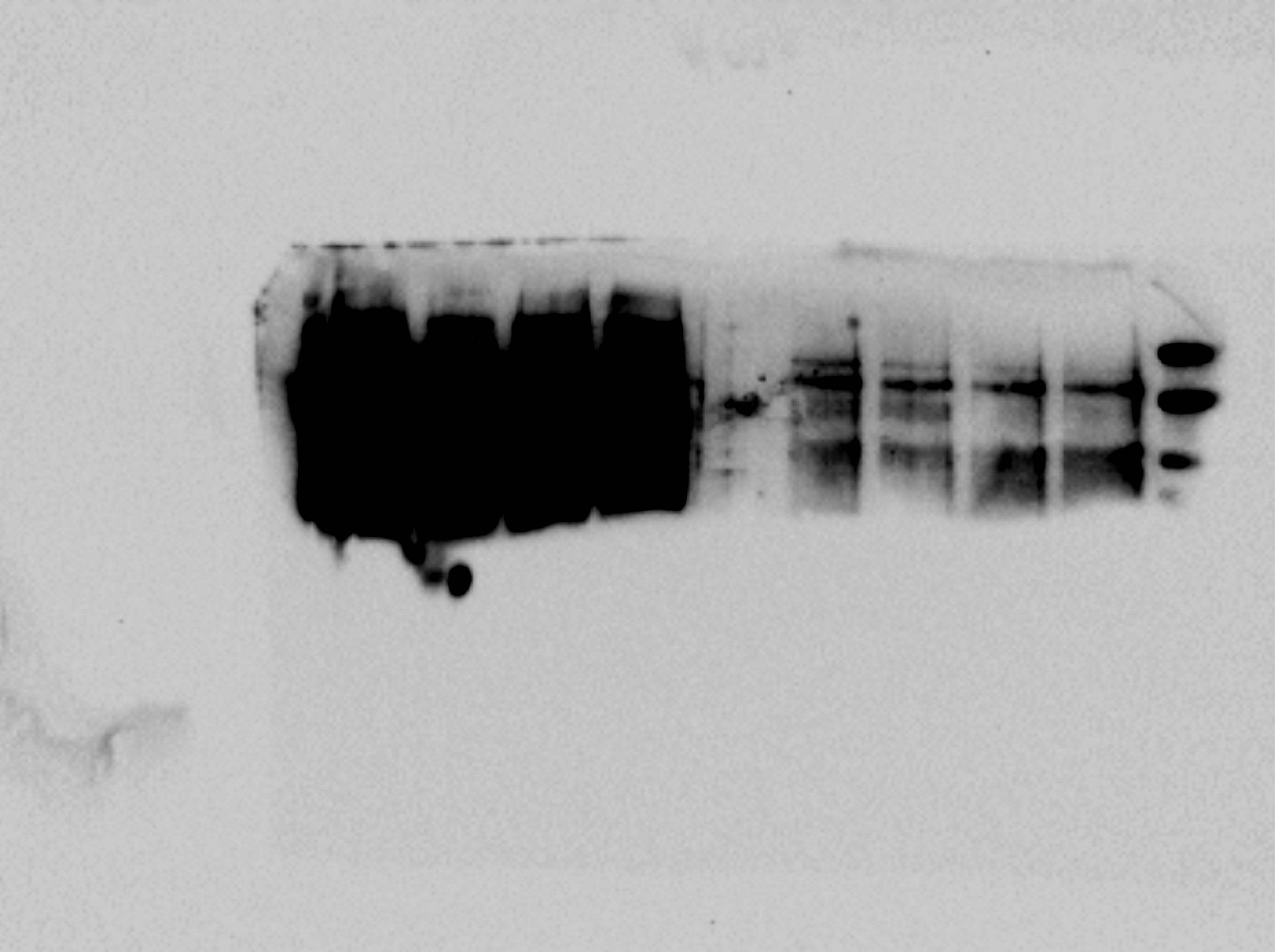


**150kDa**

**Input**

**TNF-α (h)**

**0 24**

**IP: YEATS2**

**0 24**

**IP: YEATS2**

**0 24**

**TNF-α (h)**

**4G10 in Phosphatase assay in HPNE**

**37kDa**

**Input**

**TNF-α (h)**

**0 24**

**GAPDH in Phosphatase assay in HPNE**

**GAPDH**

**4G10**

**YEATS2**

**Fig 3H**
