# Supplementary material for: Dynamic interaction of MYC enhancer RNA with YEATS2 protein regulates MYC gene transcription in pancreatic cancer
Source: EMBO Rep. 2025 Apr 11;26(10):2519–44. doi: 10.1038/s44319-025-00446-0 (PMC12117045; doi:10.1038/s44319-025-00446-0)
Supplement: Supplementary file 7 — Source data Fig. 3 [file 44319_2025_446_MOESM7_ESM.zip › Figure 3/3I/README.docx]

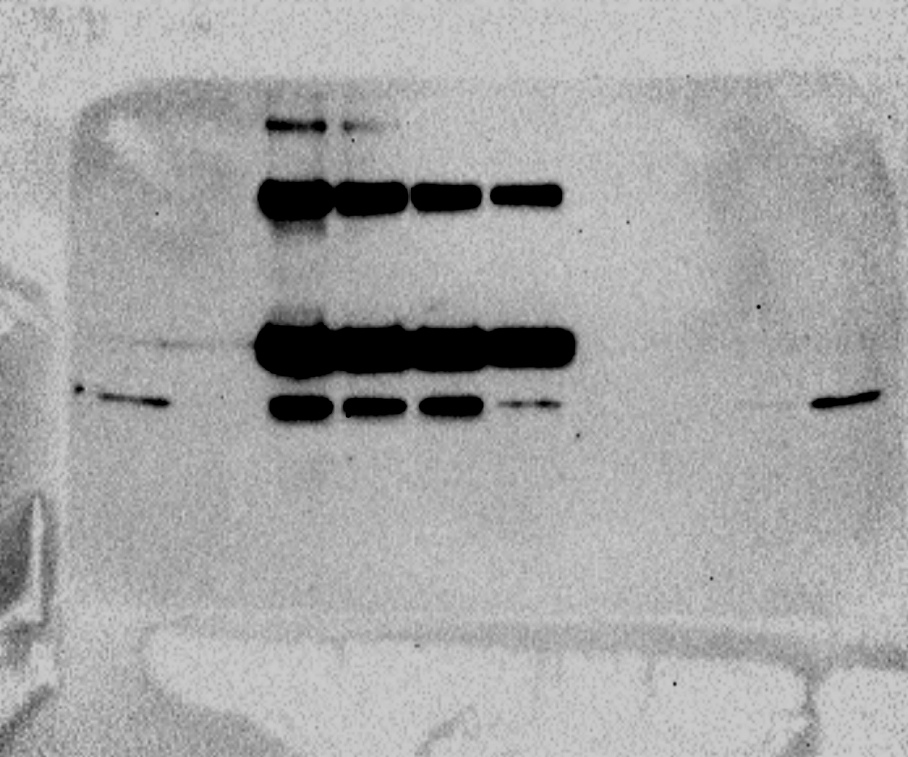


**18kDa**

**Input**

**HEK293T**

**FLAG**

**TNF-α (h)**

**IP: FLAG**

**0 24 -ve C**

**MIAPaCa-2 lysate**

Flag level in-vitro Phosphatase assay in HEK293T


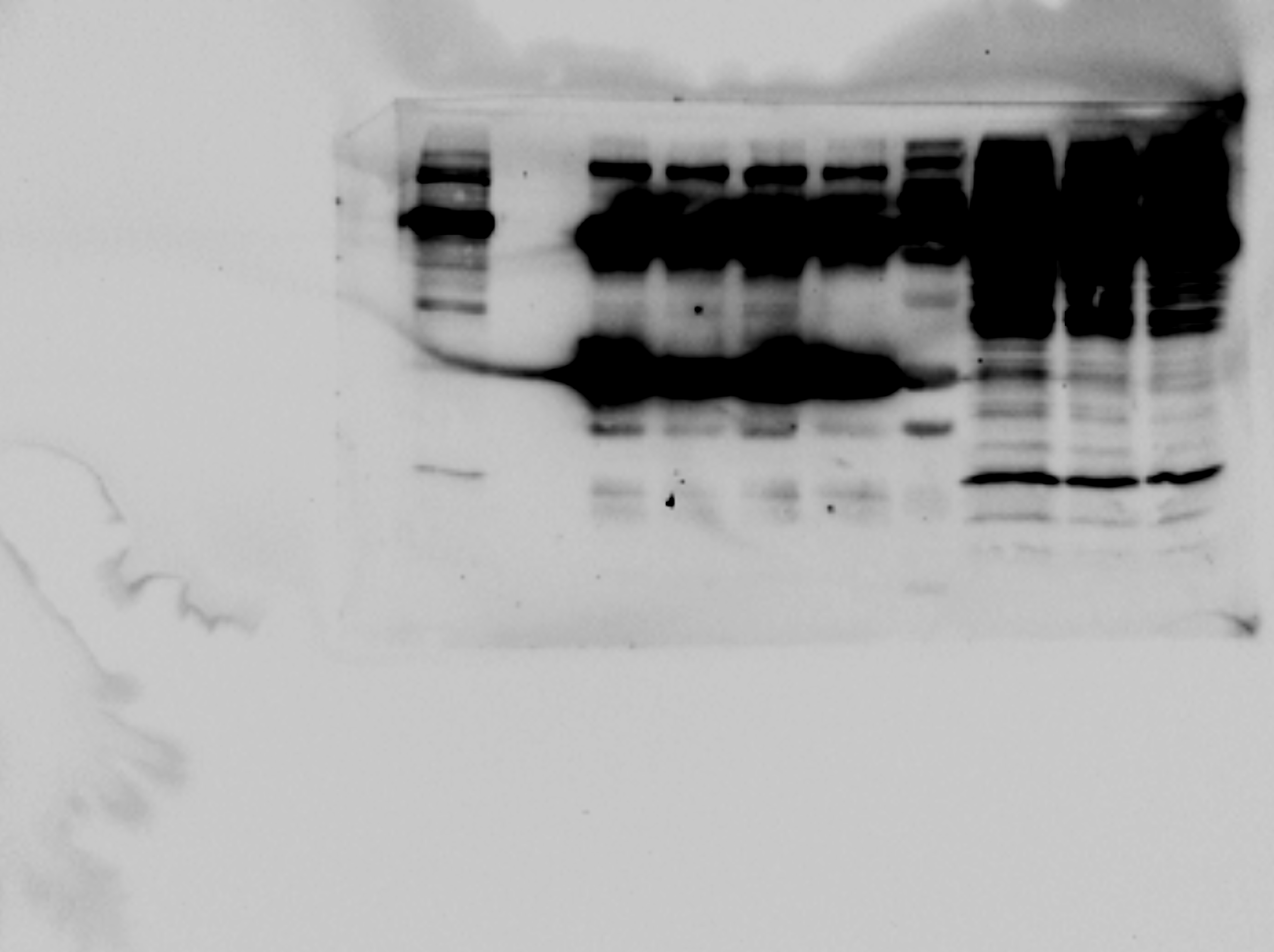


**18kDa**

**0 24 -ve C**

**TNF-α (h)**

**4G10**

**IP: FLAG**

**MIAPaCa-2 lysate**

**HEK293T**

**Fig 3I**

4G10 level in-vitro Phosphatase assay in HEK293T
