# Supplementary material for: Dynamic interaction of MYC enhancer RNA with YEATS2 protein regulates MYC gene transcription in pancreatic cancer
Source: EMBO Rep. 2025 Apr 11;26(10):2519–44. doi: 10.1038/s44319-025-00446-0 (PMC12117045; doi:10.1038/s44319-025-00446-0)
Supplement: Supplementary file 7 — Source data Fig. 3 [file 44319_2025_446_MOESM7_ESM.zip › Figure 3/3K/README.docx]

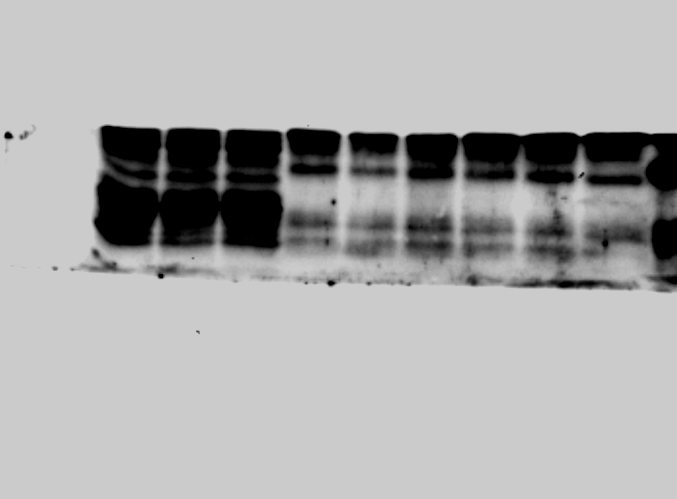


**18kDa**

4G10 level in-vitro Phosphatase assay of mutant in HEK293T

**4G10**

**IP: Flag**

**Y313_0h**

**Y313_ 24h**

**Y313F_0h**

**Y313F_24h**

**TNF-α**

**Fig 3K**


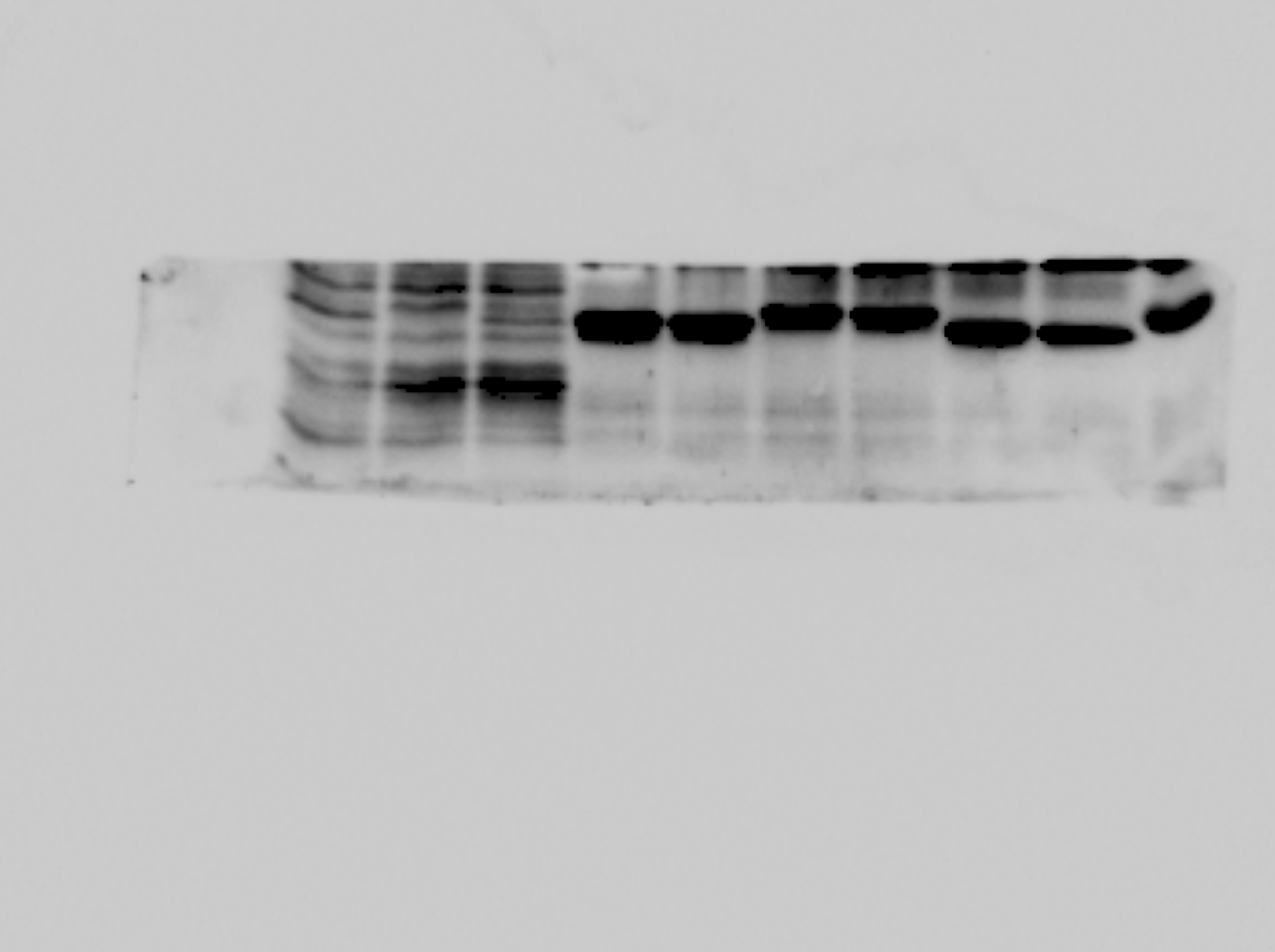


**18kDa**

**Y313_0h**

**Y313_ 24h**

**Y313F_0h**

**Y313F_24h**

**TNF-α**

**Flag**

**IP: Flag**


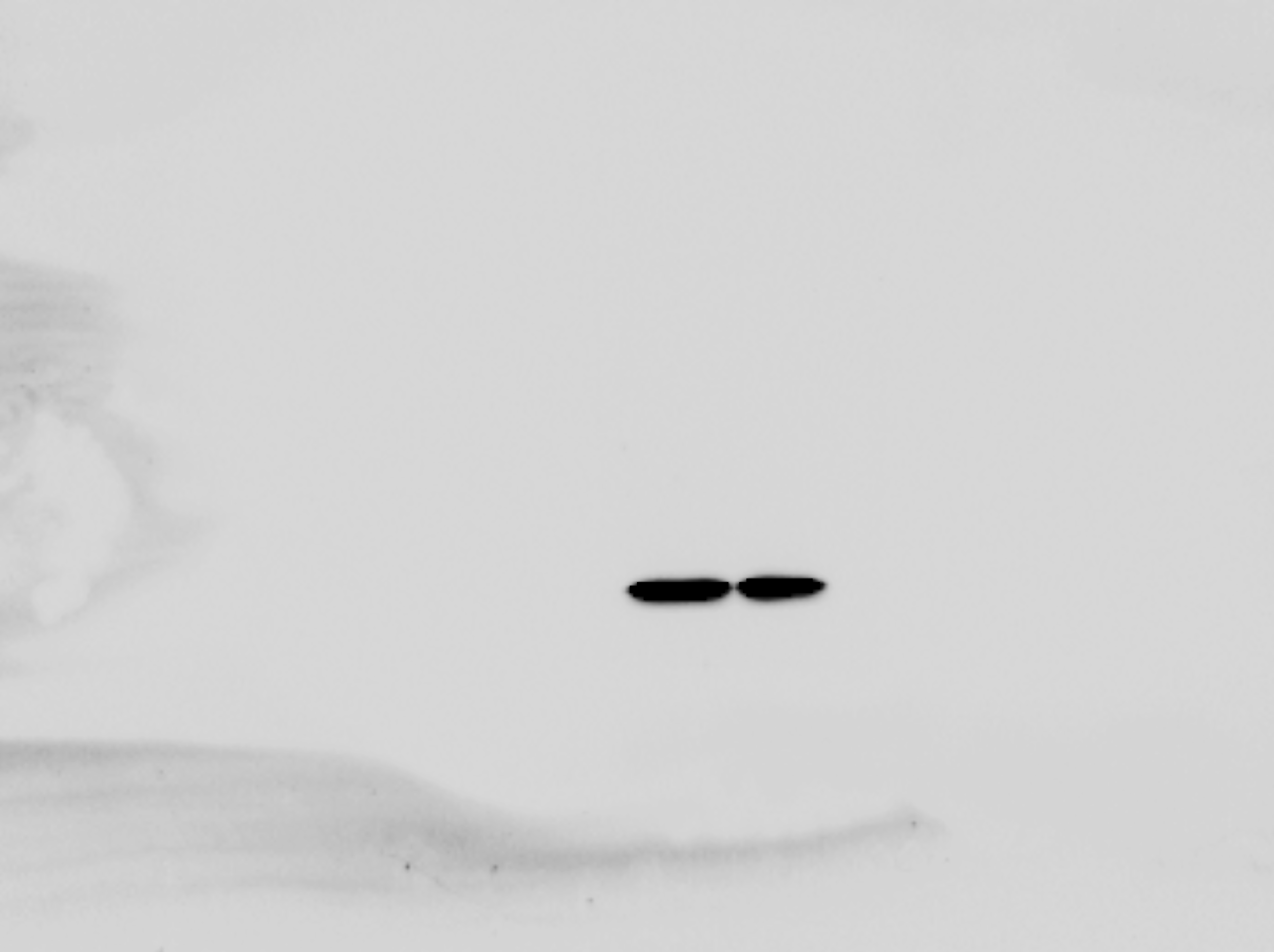


Flag level in-vitro Phosphatase assay of mutant in HEK293T

Input

**Y313_0h**

**Y313_ 24h**

**18kDa**

**Flag**

**TNF-α**
