# Supplementary material for: Dynamic interaction of MYC enhancer RNA with YEATS2 protein regulates MYC gene transcription in pancreatic cancer
Source: EMBO Rep. 2025 Apr 11;26(10):2519–44. doi: 10.1038/s44319-025-00446-0 (PMC12117045; doi:10.1038/s44319-025-00446-0)
Supplement: Supplementary file 8 — Source data Fig. 4 [file 44319_2025_446_MOESM8_ESM.zip › Figure 4/4F/Upper Panel/README.docx]

**IP: Histone H3**

**WB: YEATS2 in MIAPaCa-2**


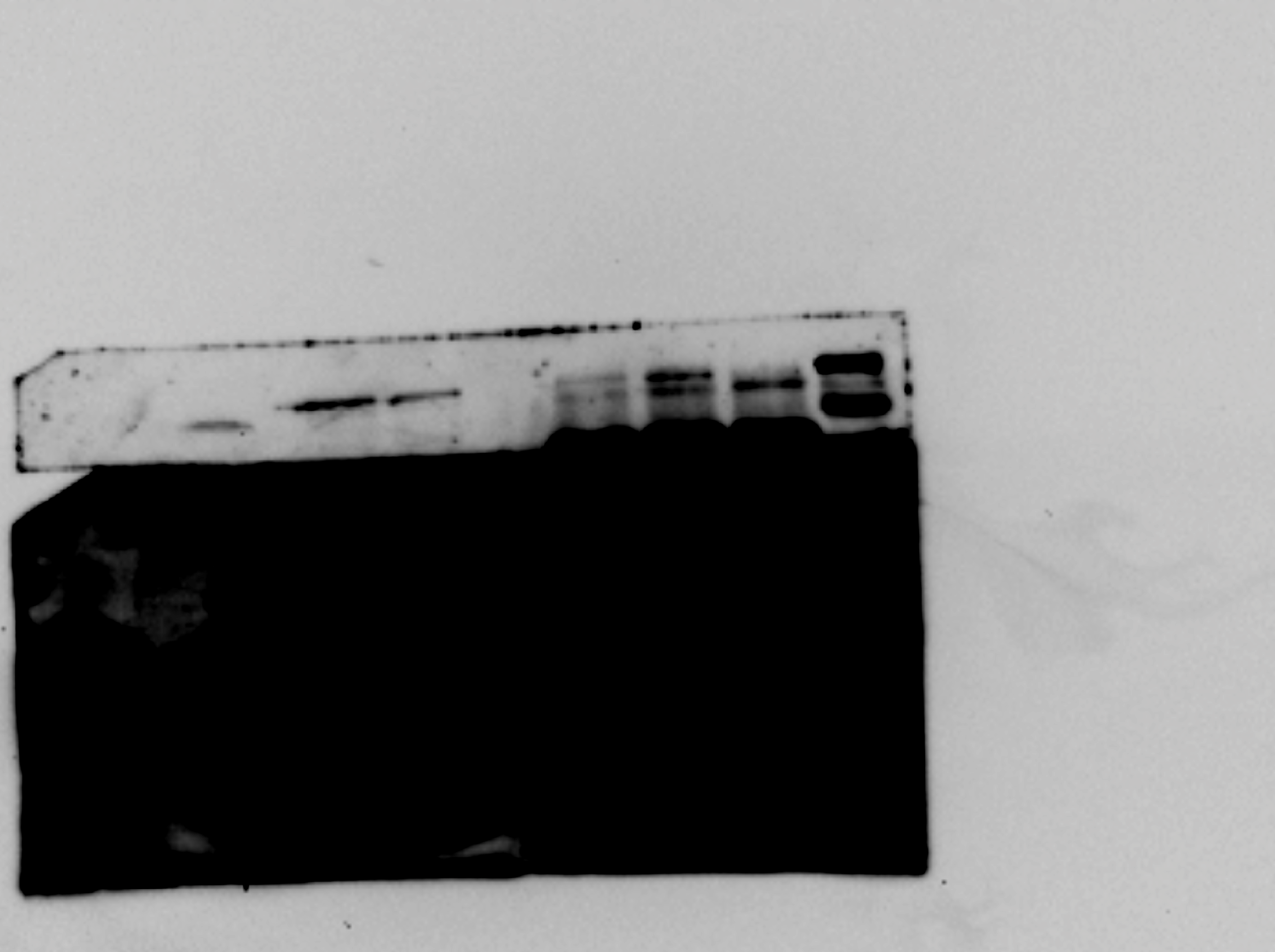

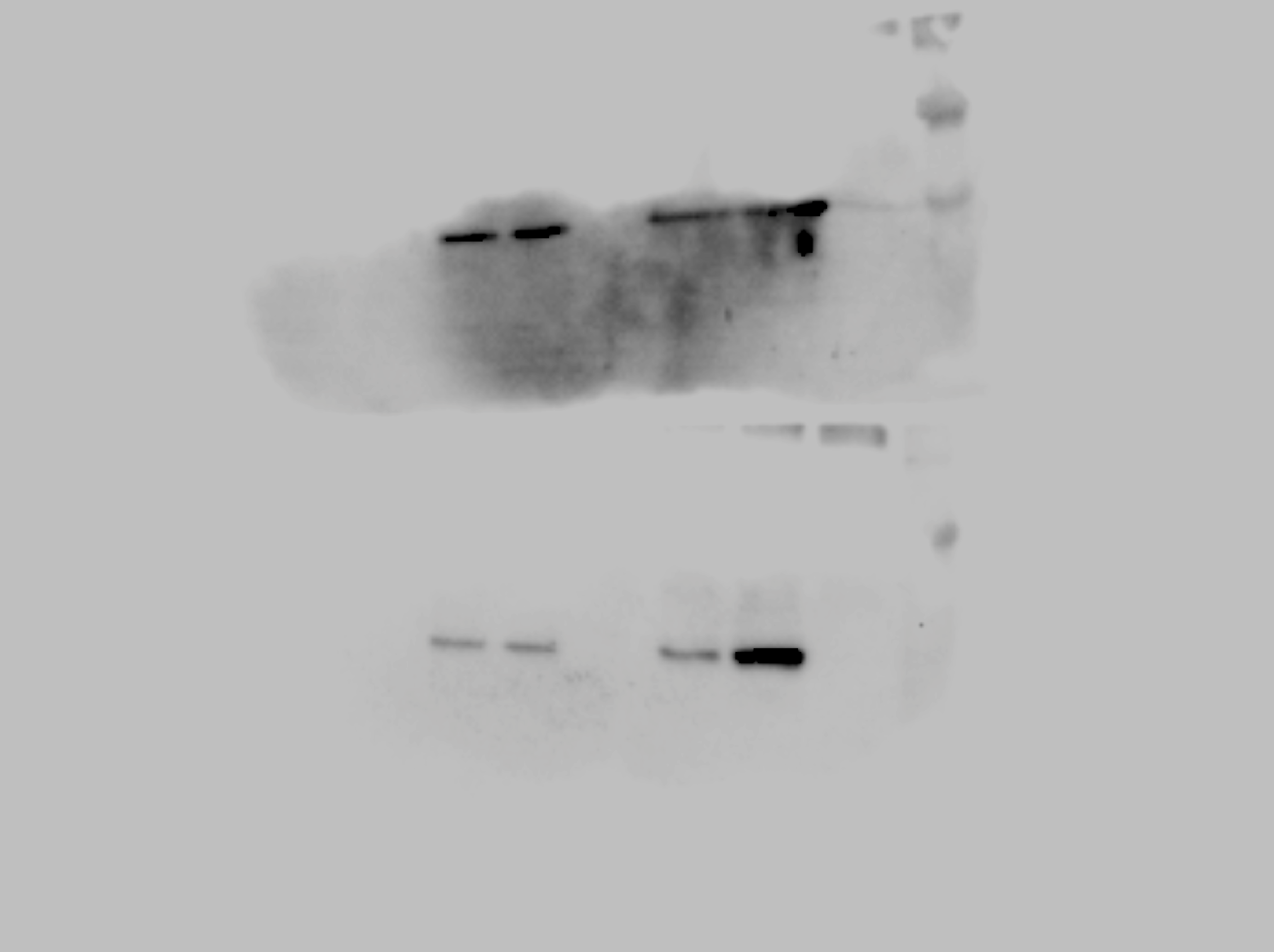


**Input**

**24**

**0**

**TNF-α (h)**

**0**

**24**

**IP:H3**

**H3**

**IP:IgG**

**-**

**17kDa**

**150kDa**

**TNF-α (h)**

**0**

**24**

**0**

**24**

**-**

**Input**

**IP:H3**

**IP:IgG**

**YEATS2**

**IP: Histone H3**

**WB: Histone H3 in MIAPaCa-2**

**Fig 4F upper panel**
