# Supplementary material for: Dynamic interaction of MYC enhancer RNA with YEATS2 protein regulates MYC gene transcription in pancreatic cancer
Source: EMBO Rep. 2025 Apr 11;26(10):2519–44. doi: 10.1038/s44319-025-00446-0 (PMC12117045; doi:10.1038/s44319-025-00446-0)
Supplement: Supplementary file 9 — Source data Fig. 5 [file 44319_2025_446_MOESM9_ESM.zip › Figure 5/5B/Image/Micr. Image.docx]

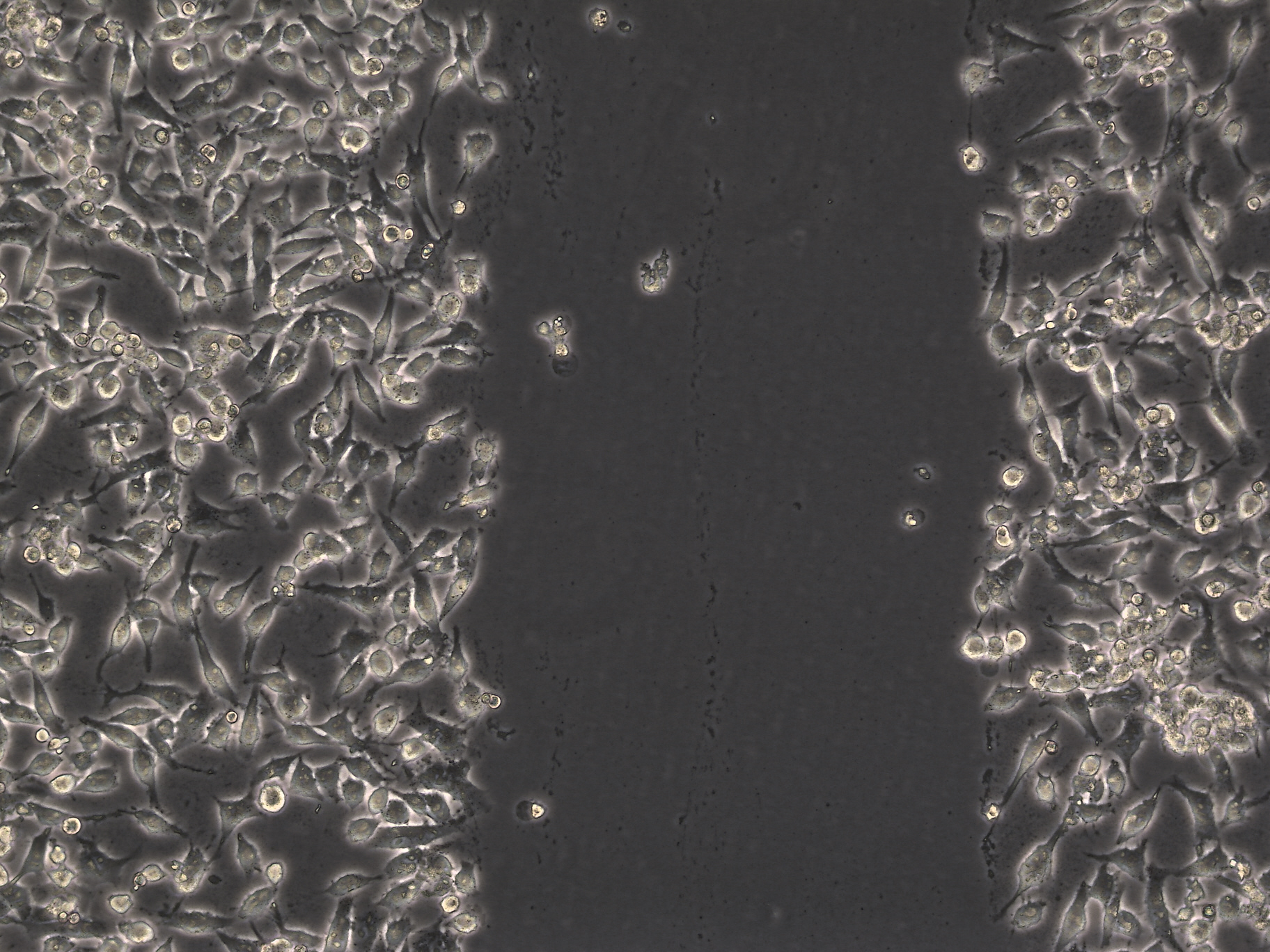

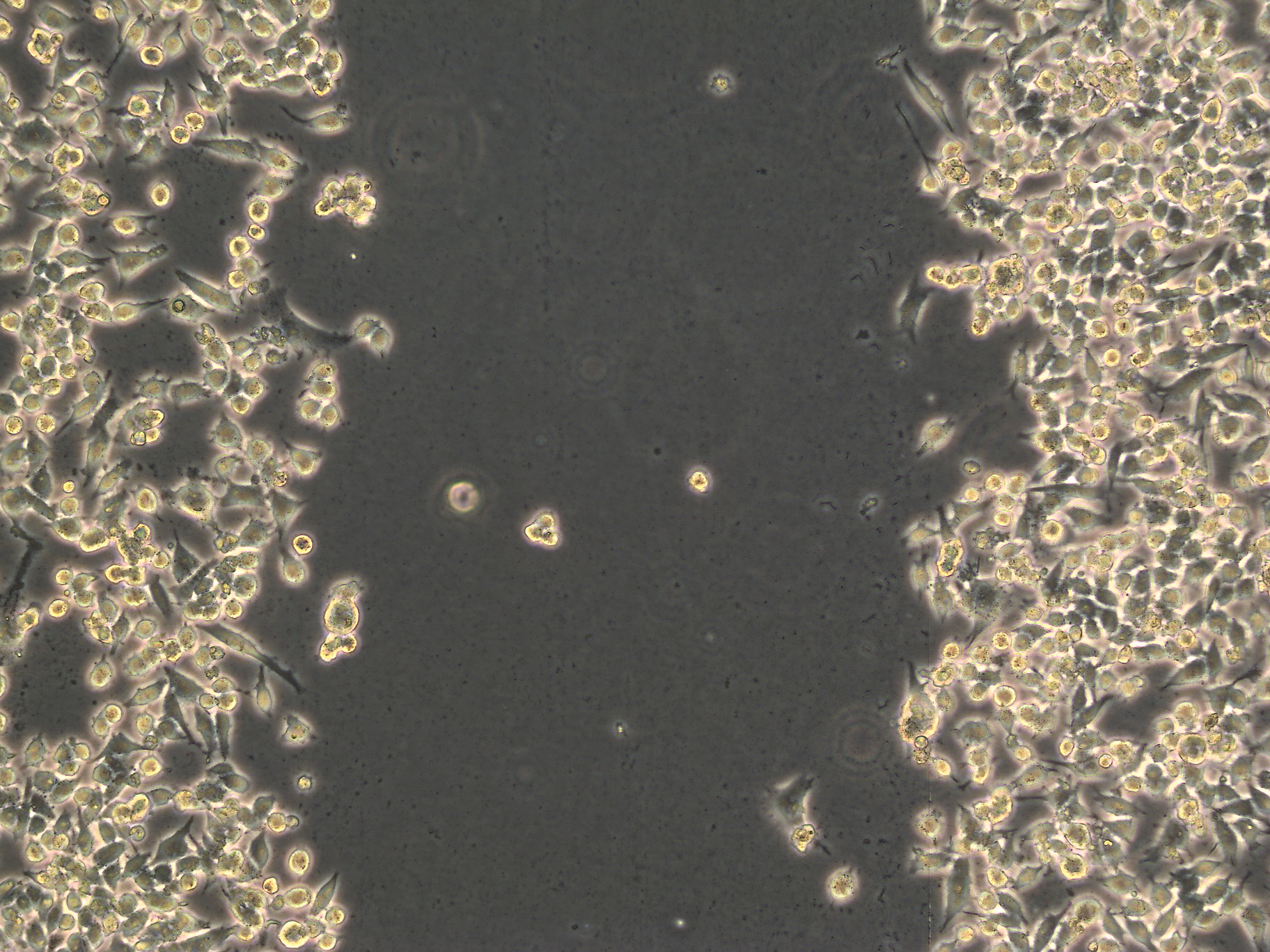

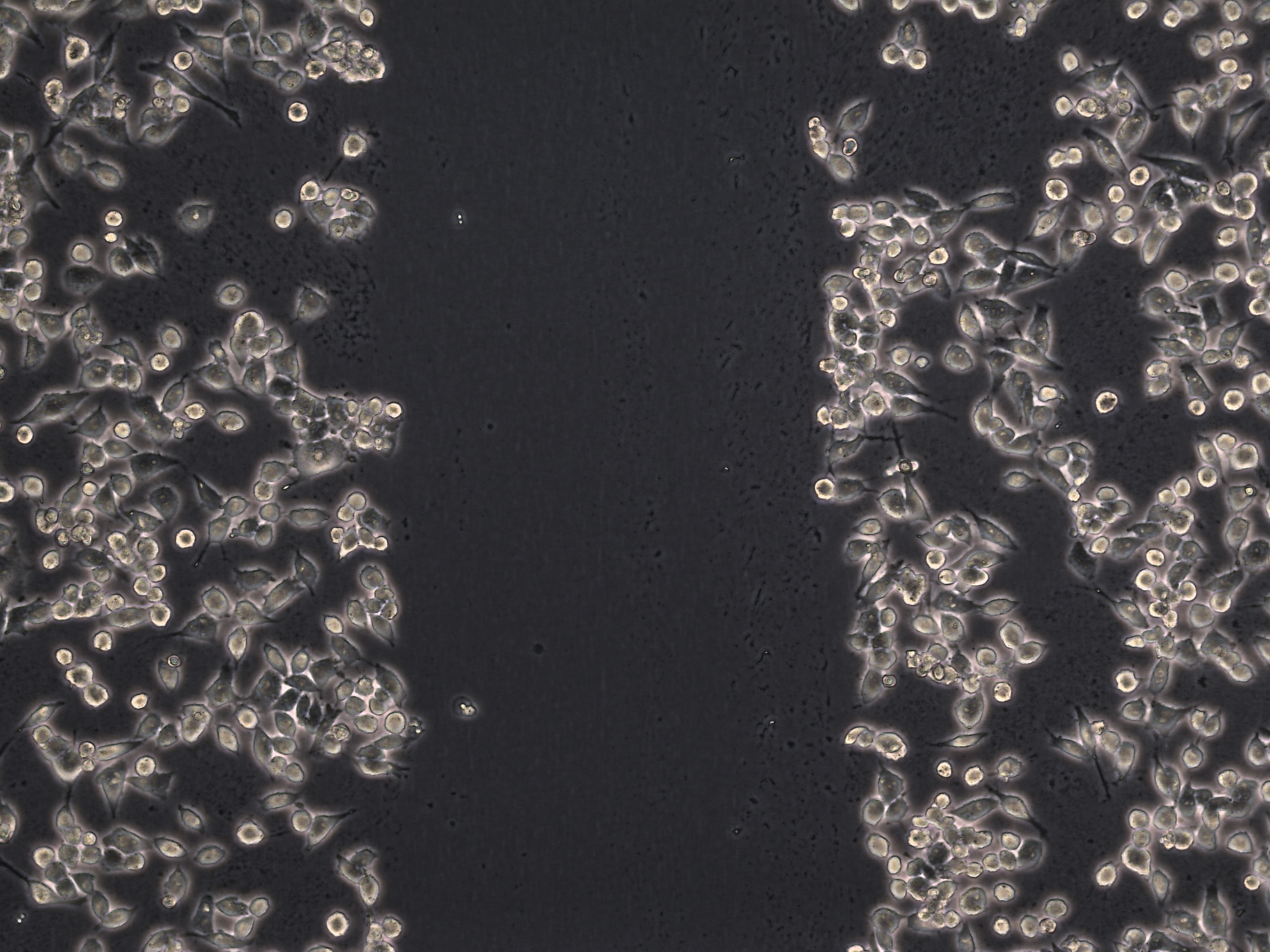

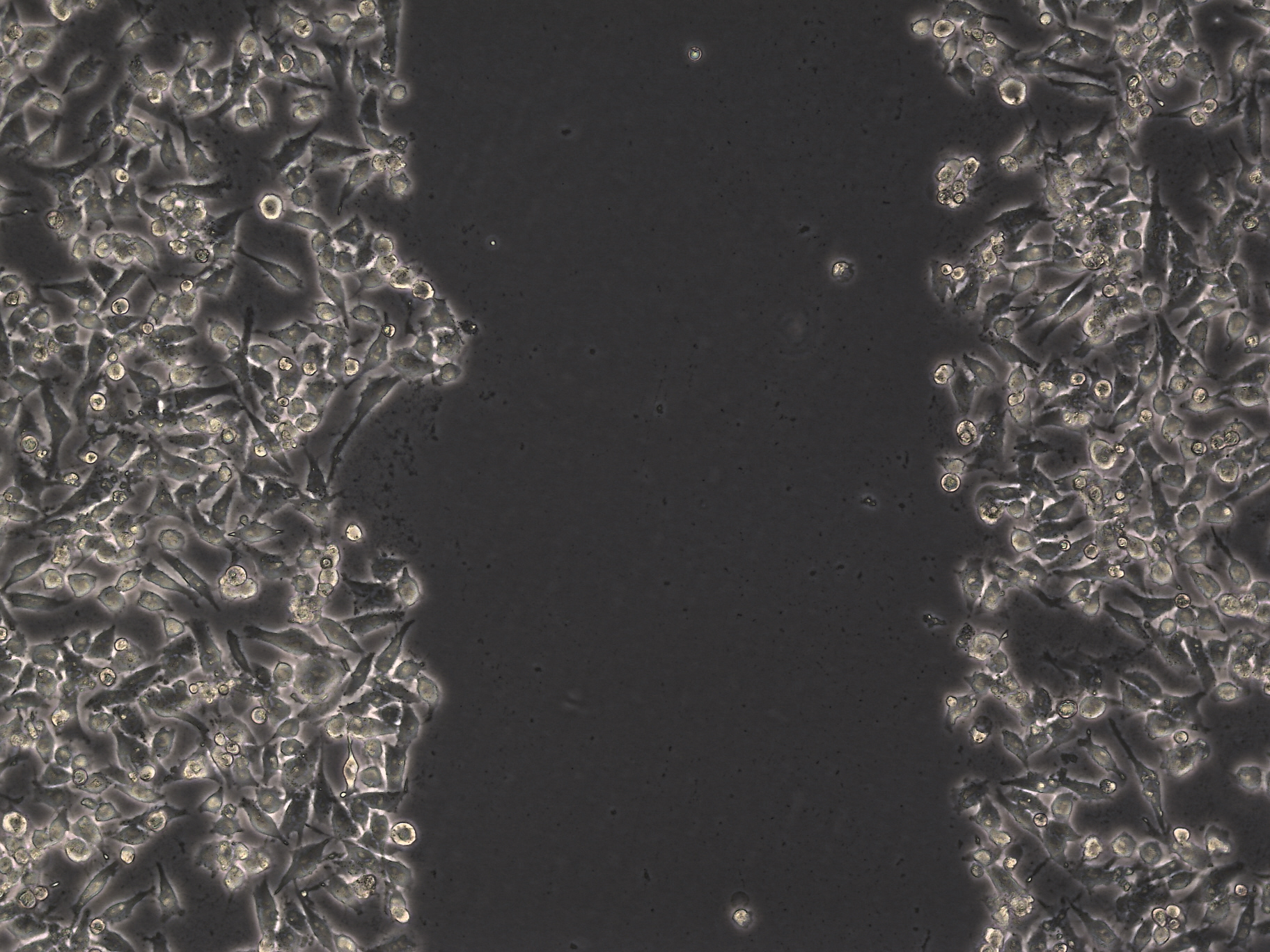


**-TNFα**

**+TNFα**

**VC**

*MYC*-490eRNA

**Fig 5B**

**0h**


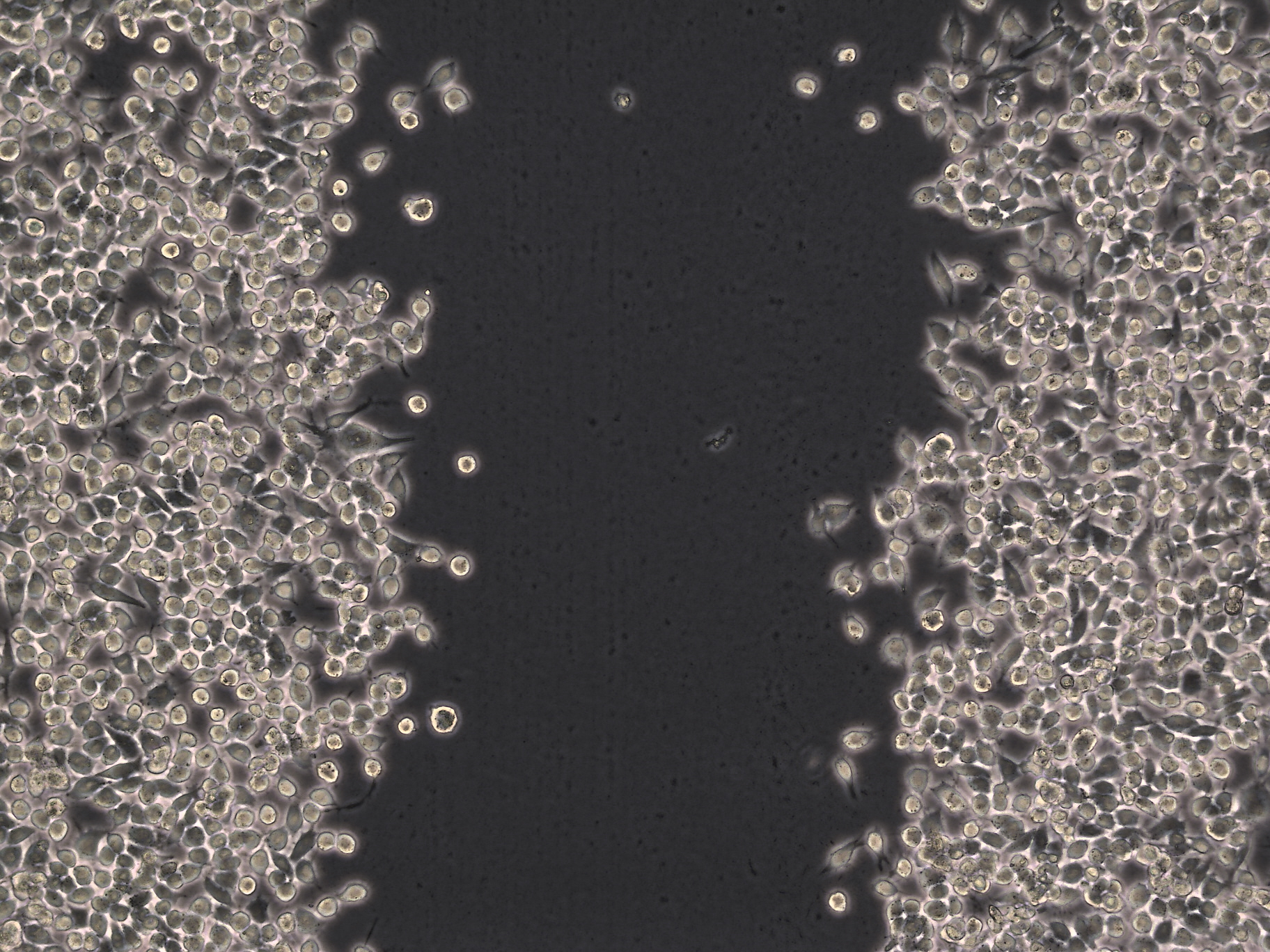

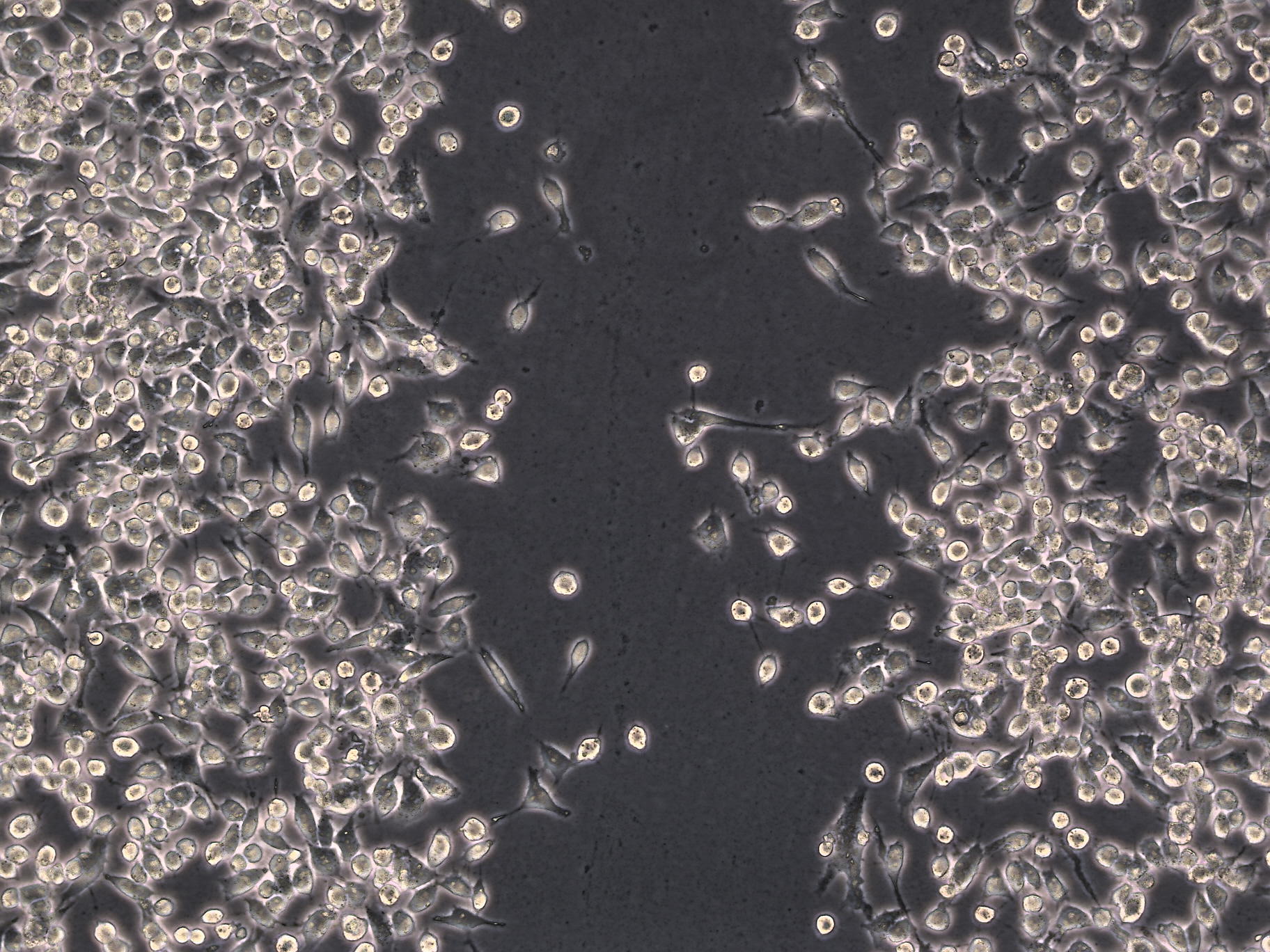

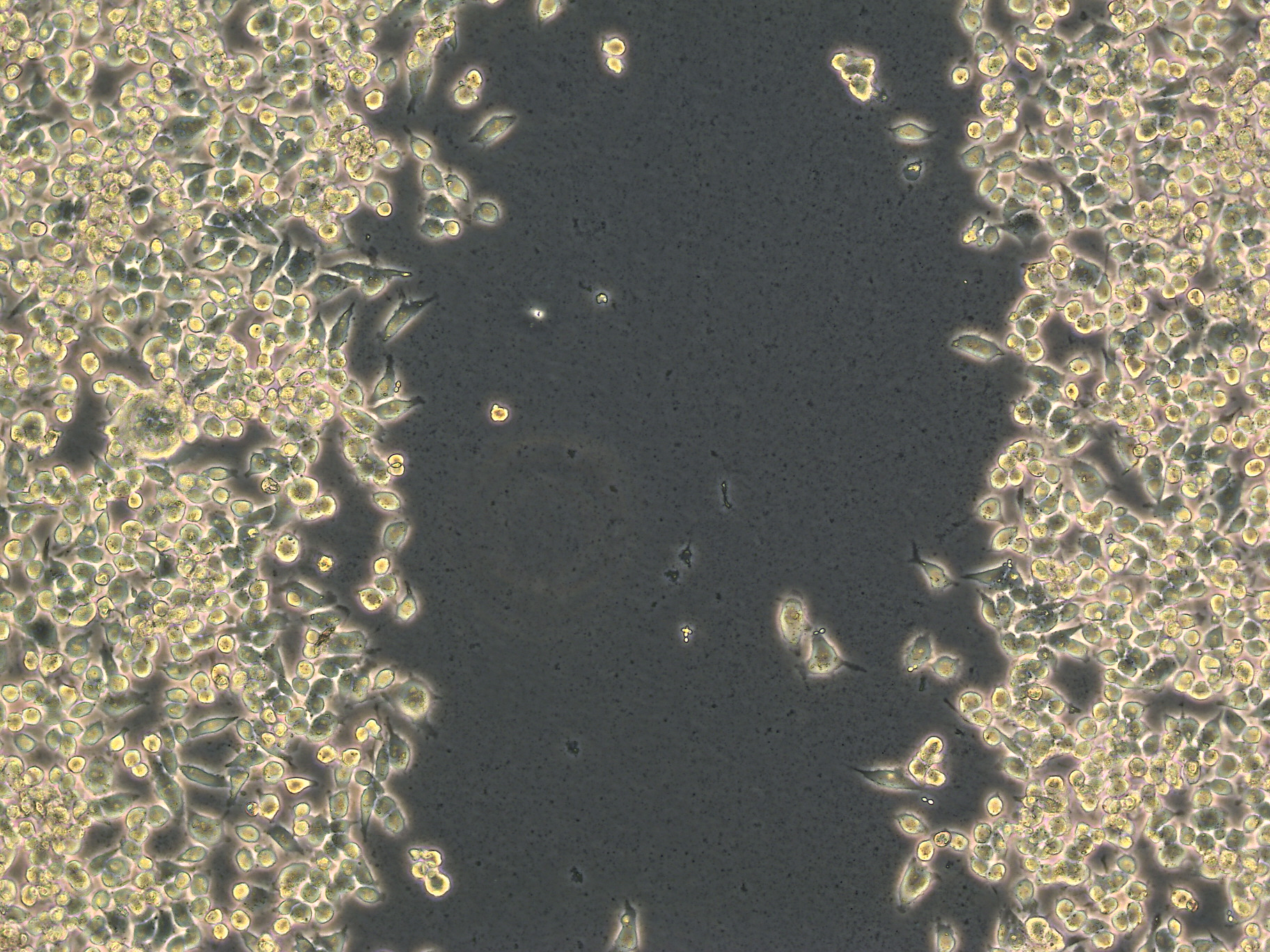

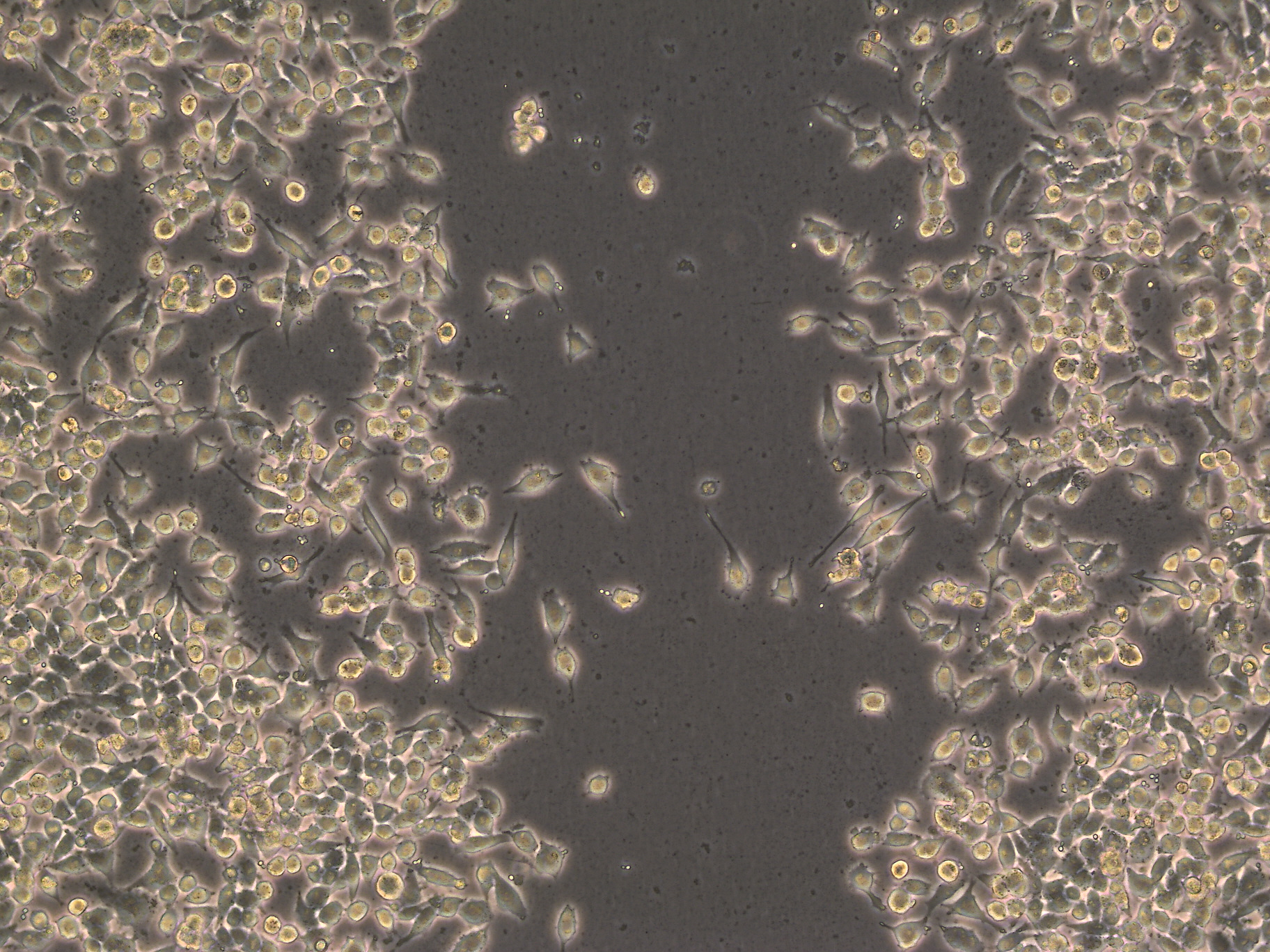


**-TNFα**

**+TNFα**

**VC**

*MYC*-490eRNA

**Fig 5B**

**24h**


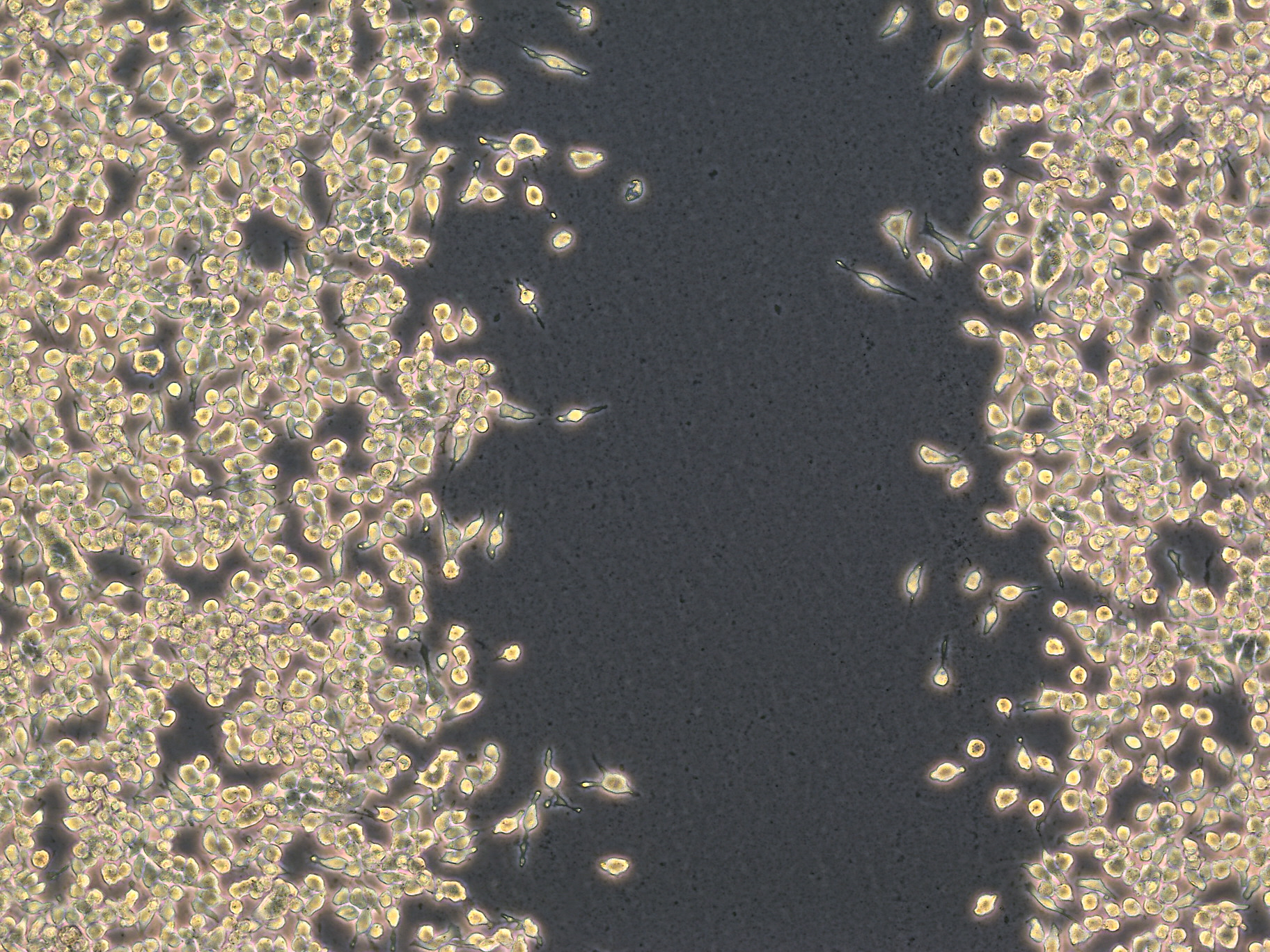

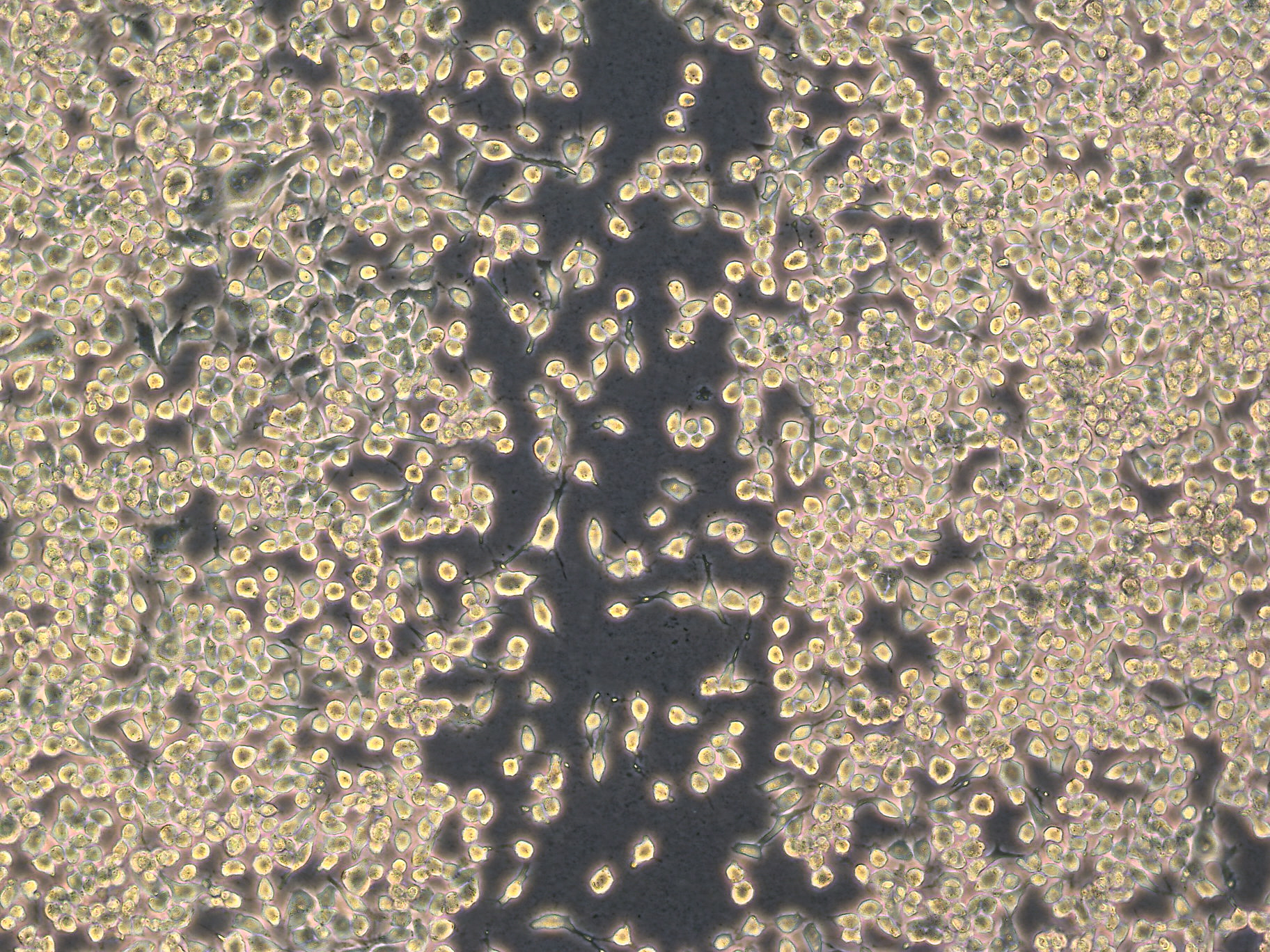

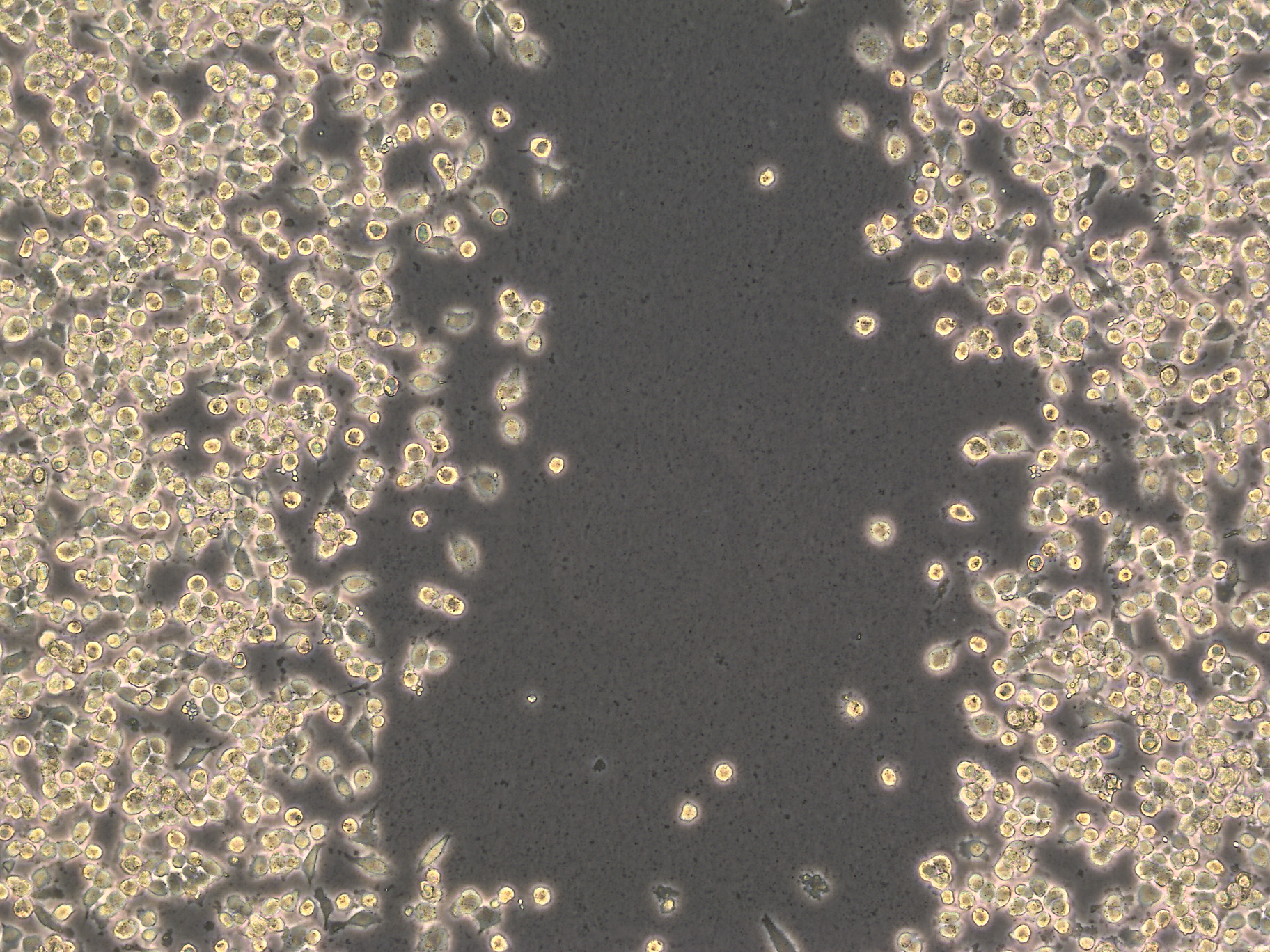

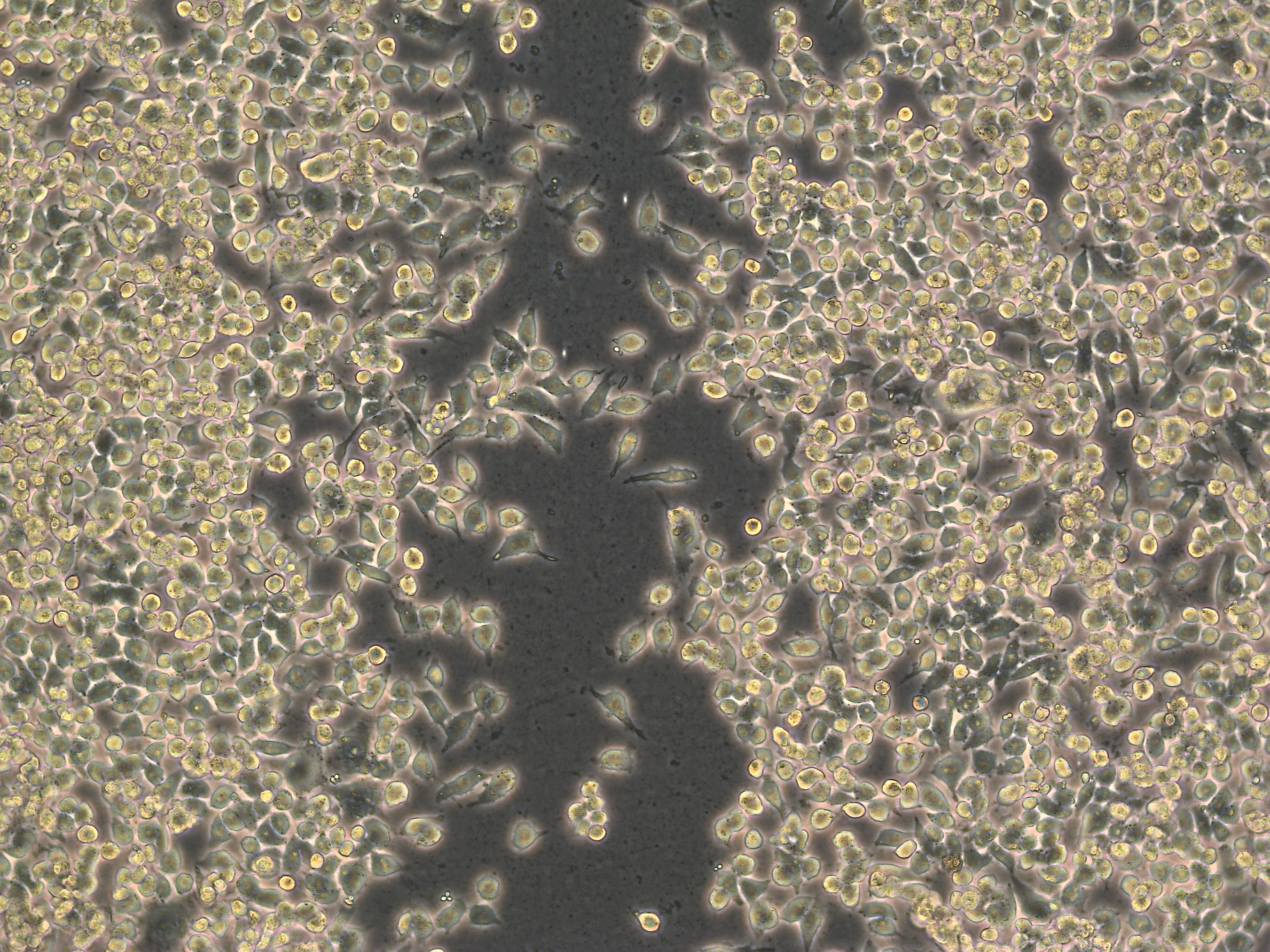


**-TNFα**

**+TNFα**

**VC**

*MYC*-490eRNA

**Fig 5B**

**48h**


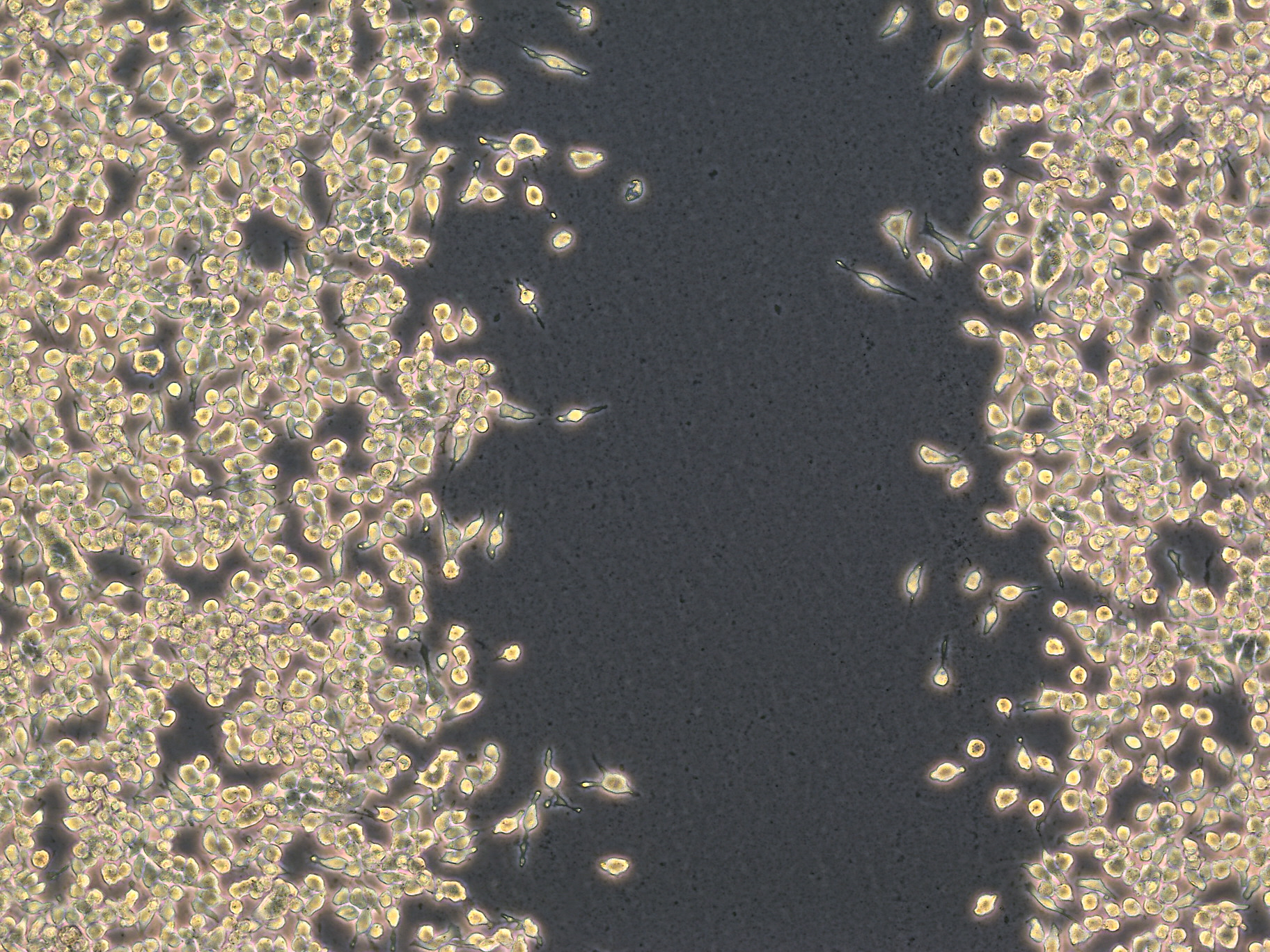

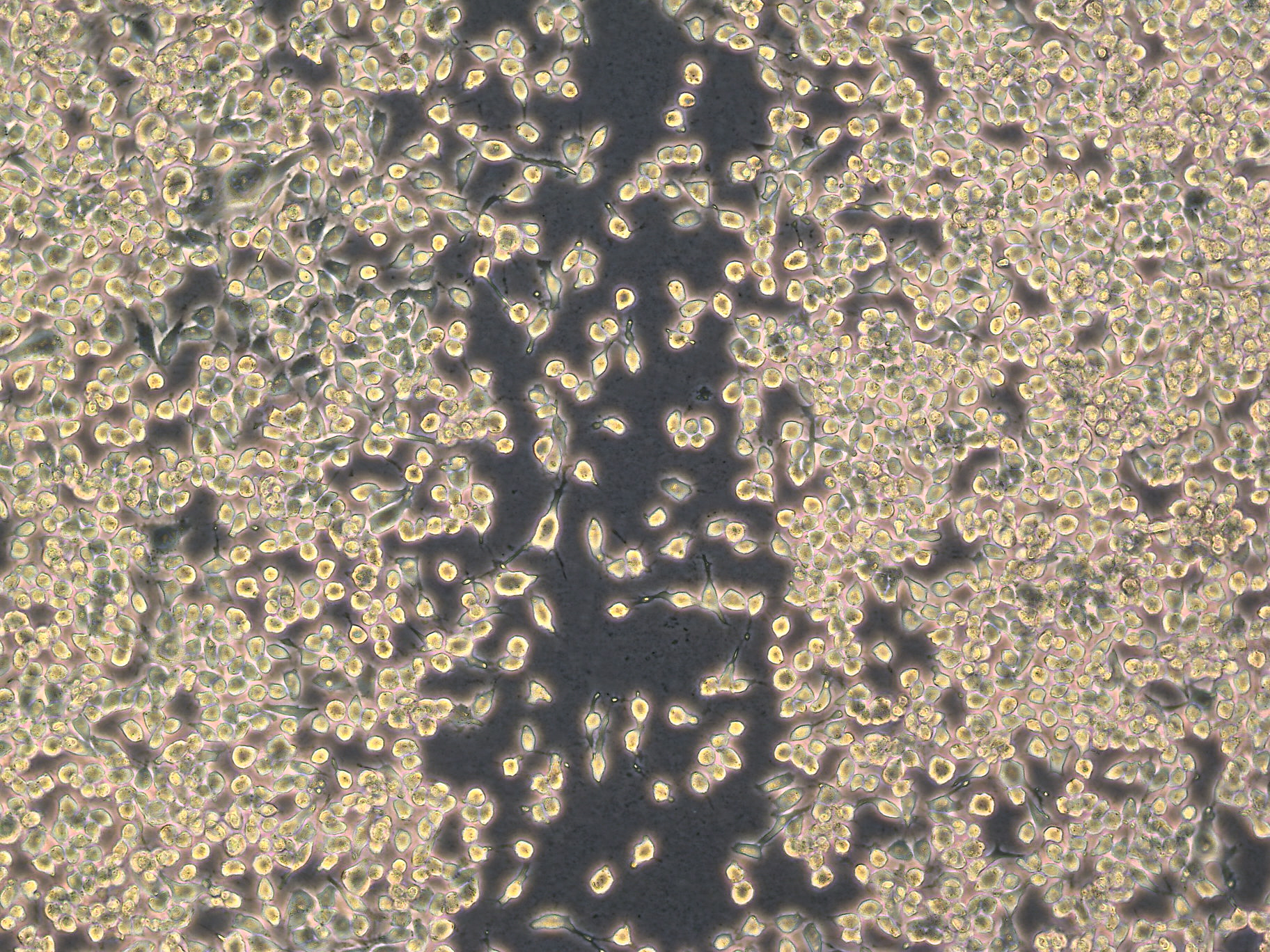

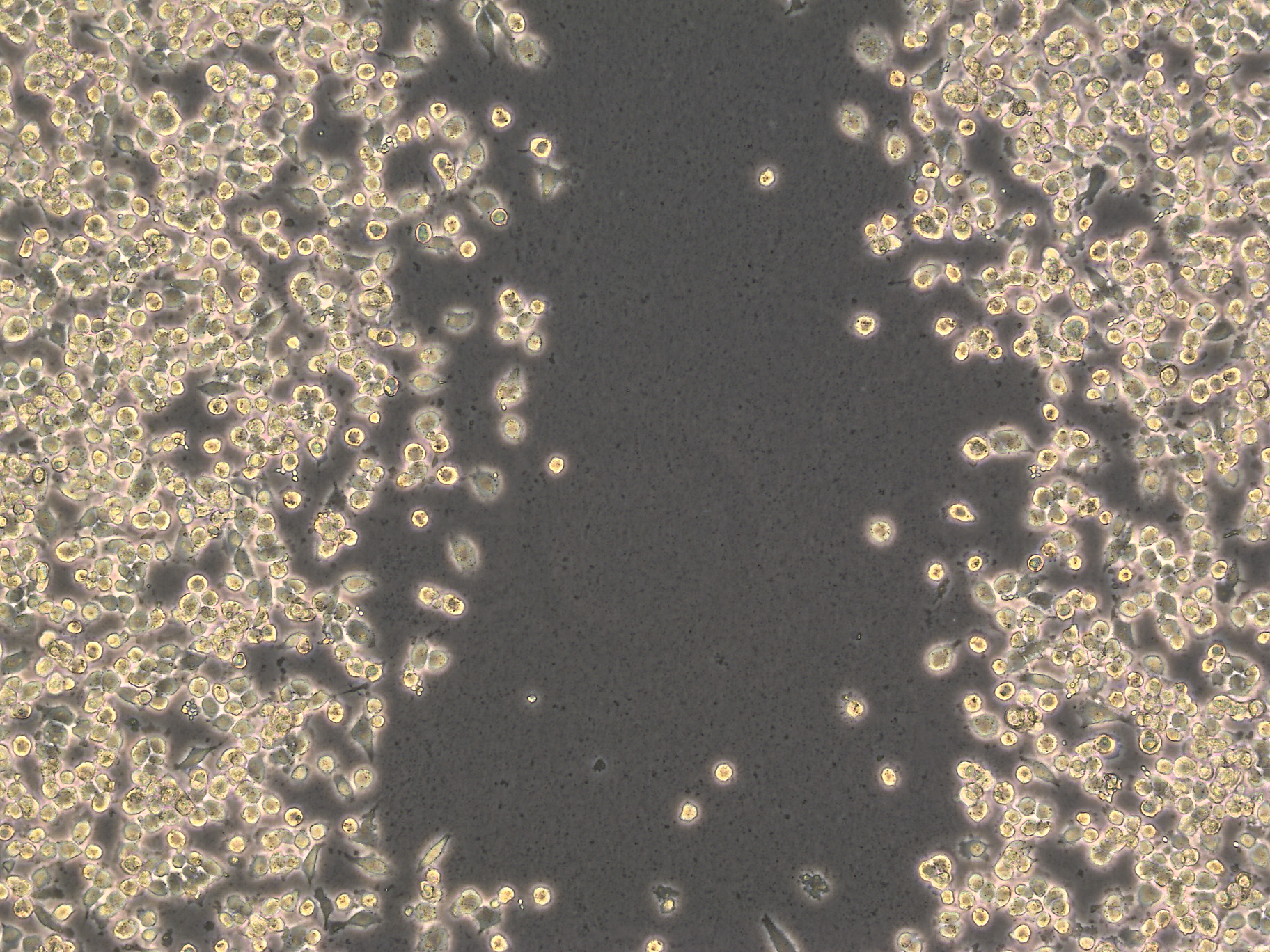

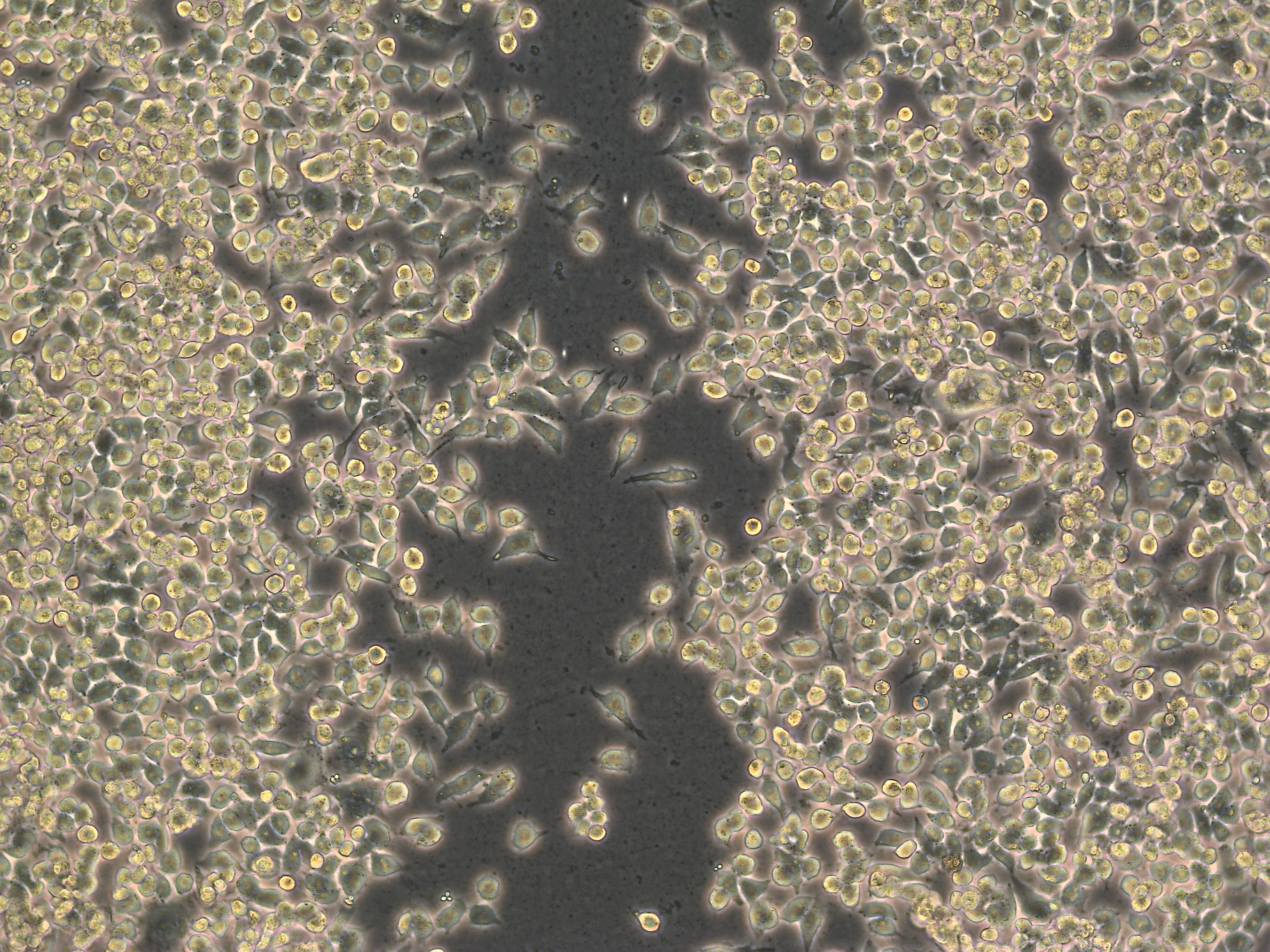


**-TNFα**

**+TNFα**

**VC**

*MYC*-490eRNA

**Fig 5B**

**72h**
