# Supplementary material for: Dynamic interaction of MYC enhancer RNA with YEATS2 protein regulates MYC gene transcription in pancreatic cancer
Source: EMBO Rep. 2025 Apr 11;26(10):2519–44. doi: 10.1038/s44319-025-00446-0 (PMC12117045; doi:10.1038/s44319-025-00446-0)
Supplement: Supplementary file 10 — Expanded View Figures [file 44319_2025_446_MOESM10_ESM.pdf]

Expanded View Figures

Figure EV1. Induction of chronic inflammation in mammalian cell lines.

(**A**) Survival plot analysis depicting the impact of *MYC* amplification status on the overall survival of patients in the GSE-62452 cohort. Through 24 h treatment of TNF-α, a chronic inflammatory condition was mimicked in (**B**) MIAPaCa-2, data are presented as mean ± SD from three independent experiments ($n = 3$). Statistical significance was determined using an unpaired two-tailed *t* test: ***$P = 0.0003$ and ***$P = 0.0002$ for IL-6 and IL-1β respectively and (**C**) AsPC-1, data are presented as mean ± SD from three independent experiments ($n = 3$). Statistical significance was determined using an unpaired two-tailed *t* test: ***$P = 0.00015$ and ****$P = < 0.0001$ for IL-6 and IL-1β respectively. (**D**) HPNE cell lines which has been shown by significant increase in the expression level of pro-inflammatory cytokines IL-6 and IL-1β. Data are presented as mean ± SD from three independent experiments ($n = 3$). Statistical significance was determined using an unpaired two-tailed *t* test: ***$P = 0.0008$ and ***$P = 0.0006$ for IL-6 and IL-1β respectively. (**E**) Validation of *MYC* eRNA and *MYC* mRNA expression was performed using RT-PCR in TNF-α stimulated HCT-116 cells for 0 h and 24 h, data are presented as mean ± SD from three independent experiments ($n = 3$). Statistical significance was determined using an unpaired two-tailed *t* test: ****$P =< 0.0001$, **$P = 0.0082$ and ****$P = 0.0001$ for *MYC*-490, *MYC*-425, and *MYC* mRNA respectively and the expression level of pro-inflammatory cytokines IL-6 and IL-1β **$P = 0.0015$, ***$P = 0.0008$ respectively was measured in those conditions.

 © The Author(s)

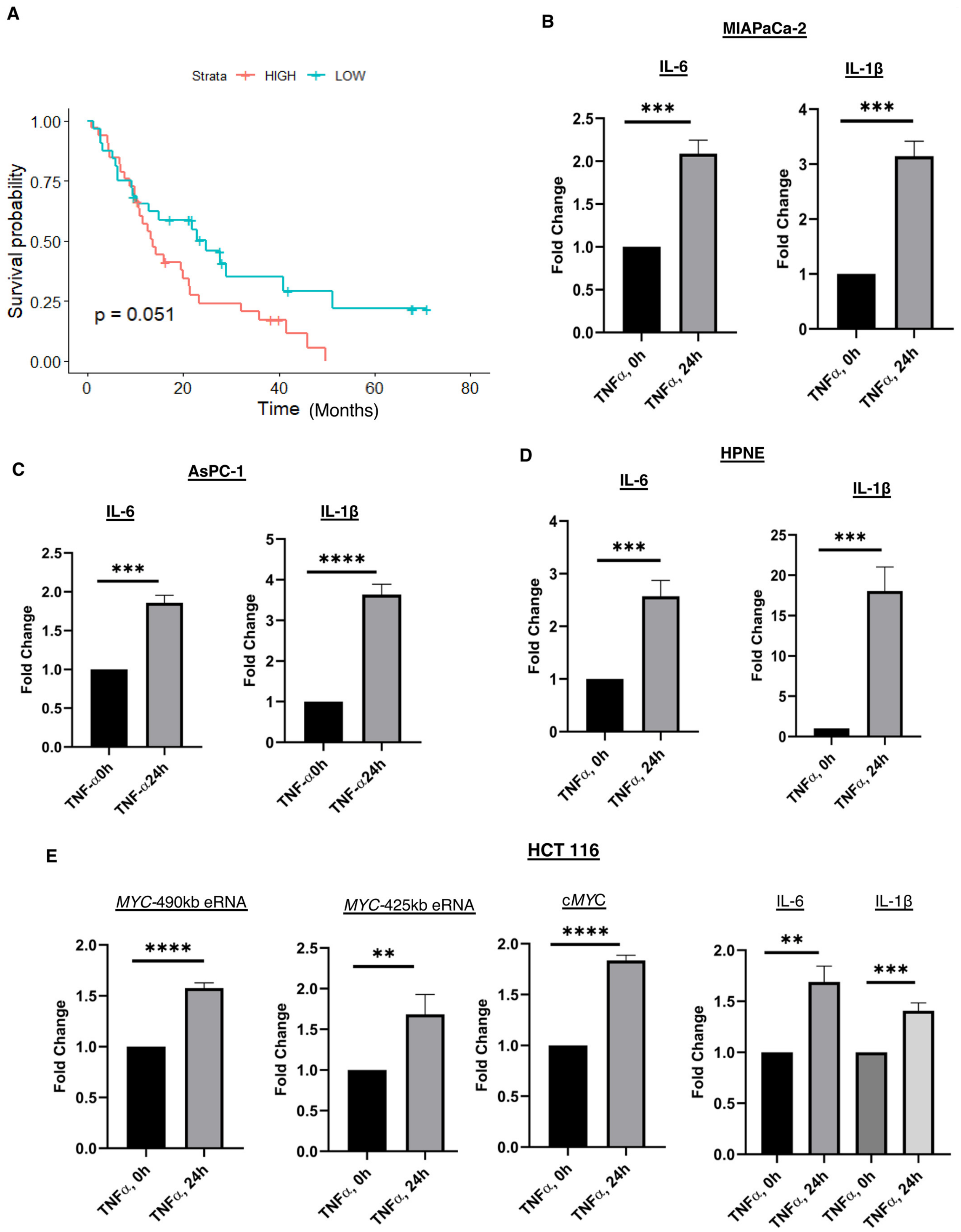
A
Strata HIGH LOW
Survival probability
1.00
0.75
0.50
0.25
0.00
p = 0.051
0 20 40 60 80
Time (Months)
B
MIAPaCa-2
IL-6
IL-1β
***
***
Fold Change
TNFα, 0h
TNFα, 24h
C
AsPC-1
IL-6
IL-1β
***
****
TNF-α0h
TNF-α24h
D
HPNE
IL-6
IL-1β
***
***
E
HCT 116
MYC-490kb eRNA
MYC-425kb eRNA
cMYC
IL-6
IL-1β
****
**
****
**
***

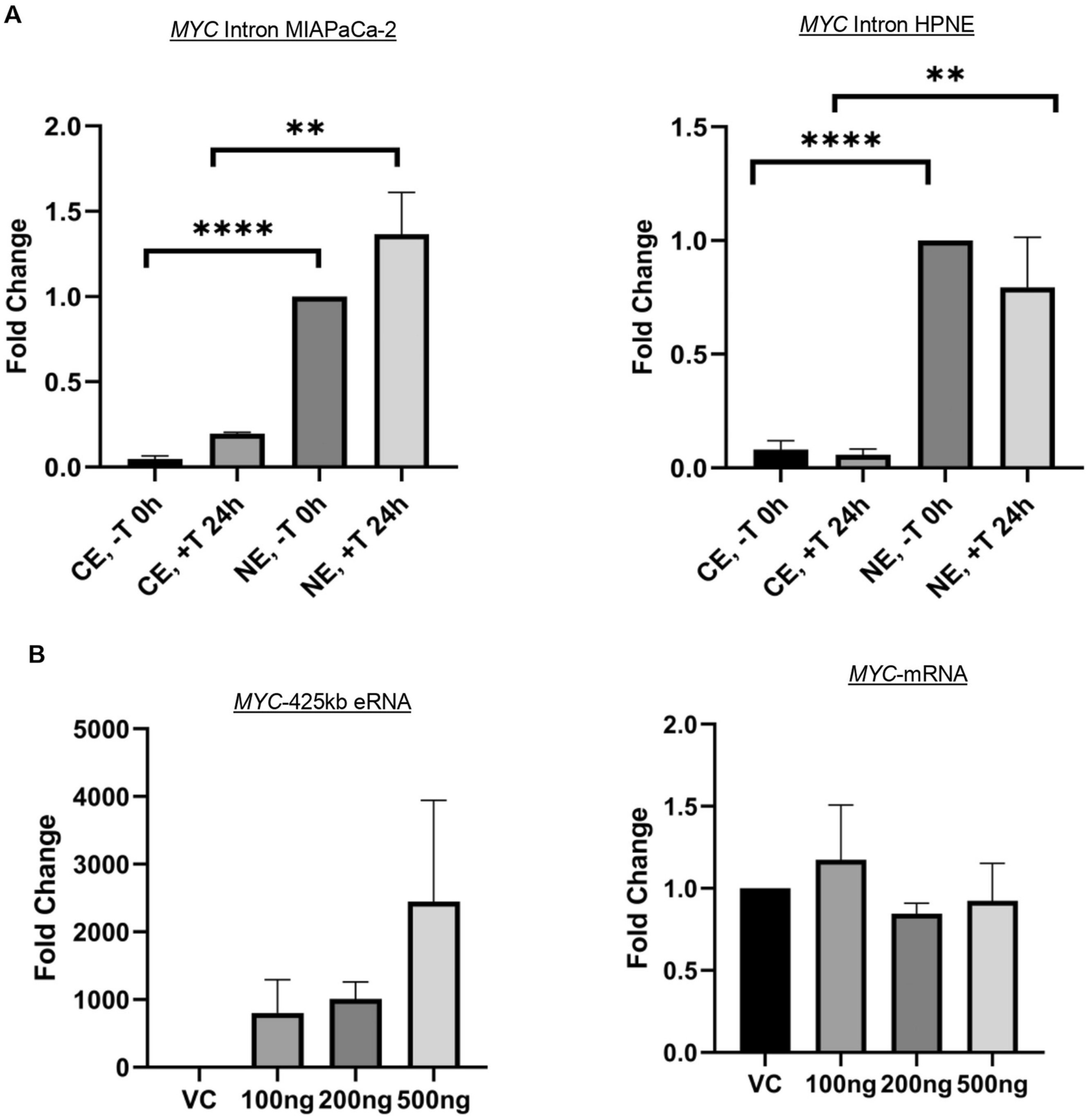

Figure EV2. Analysis of *MYC* Intron from fractions as well as *MYC*-425-kb eRNA driven *MYC* mRNA expression by RT-PCR.

(**A**) *MYC* intron RNA level was checked by qRT-PCR using intron specific primer from cytosolic and nuclear fraction of MIAPaCa-2 (left panel). Data are presented as mean ± SD from three independent experiments ($n = 3$). Statistical significance was determined using an unpaired two-tailed *t* test: ****$P = < 0.0001$ and **$P = 0.0012$ for TNF-α 0 h and 24 h respectively and HPNE cells (right panel), data are presented as mean ± SD from three independent experiments ($n = 3$). Statistical significance was determined using an unpaired two-tailed *t* test: ****$P = < 0.0001$ and **$P = 0.0045$ for TNF-α 0 h and 24 h respectively. (**B**) MIAPaCa-2 cells were transfected with plasmid expressing *MYC*-425-kb eRNA (left panel), at increasing gradient of concentration for 48 h and *MYC* mRNA expression (right panel) were checked by RT-PCR. The data represented the mean and s.e.m. of $n = 3$ independent experiments.

 © The Author(s)

A

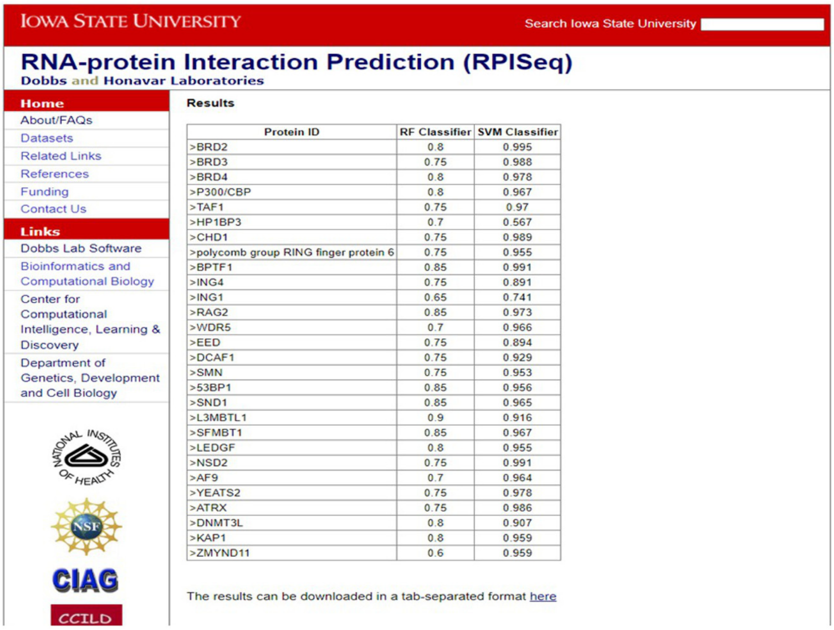
IOWA STATE UNIVERSITY — Search Iowa State University

RNA-protein Interaction Prediction (RPISeq)
Dobbs and Honavar Laboratories

Home | About/FAQs | Datasets | Related Links | References | Funding | Contact Us

Links: Dobbs Lab Software | Bioinformatics and Computational Biology | Center for Computational Intelligence, Learning & Discovery | Department of Genetics, Development and Cell Biology

Results

Protein ID	RF Classifier	SVM Classifier
>BRD2	0.8	0.995
>BRD3	0.75	0.988
>BRD4	0.8	0.978
>P300/CBP	0.8	0.967
>TAF1	0.75	0.97
>HP1BP3	0.7	0.567
>CHD1	0.75	0.989
>polycomb group RING finger protein 6	0.75	0.955
>BPTF1	0.85	0.991
>ING4	0.75	0.891
>ING1	0.65	0.741
>RAG2	0.85	0.973
>WDR5	0.7	0.966
>EED	0.75	0.894
>DCAF1	0.75	0.929
>SMN	0.75	0.953
>53BP1	0.85	0.956
>SND1	0.85	0.965
>L3MBTL1	0.9	0.916
>SFMBT1	0.85	0.967
>LEDGF	0.8	0.955
>NSD2	0.75	0.991
>AF9	0.7	0.964
>YEATS2	0.75	0.978
>ATRX	0.75	0.986
>DNMT3L	0.8	0.907
>KAP1	0.8	0.959
>ZMYND11	0.6	0.959

The results can be downloaded in a tab-separated format here

B

Protein	Hdock score
BRD2	0.9863
BRD3	0.9764
BRD4	0.9745
P300/CBP	0.9571
TAF1	0.9671
HP1BP3	0.8798
CHD1	0.9674
PCGF6	0.9250
BPTF1	0.9437
ING4	0.9072
ING1	0.9340
RAG2	0.8752
WDR5	0.9300
EED	0.9840
DCAF1	0.8880
SMN	0.8728
53BP1	0.9776
SND1	0.9546
L3MBTL1	0.9810
SFMBT1	0.9589
LEDGF	0.9187
NSD2	0.9462
AF9	0.9575
YEATS2	0.9758
ATRX	0.9737
DNMT3L	0.9697
KAP1	0.9723
ZMYND11	0.9638

C

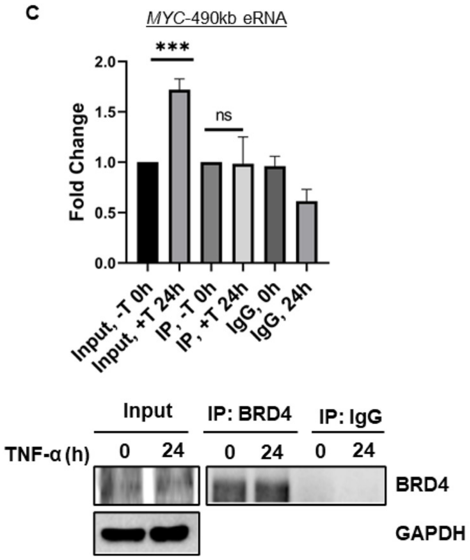

D

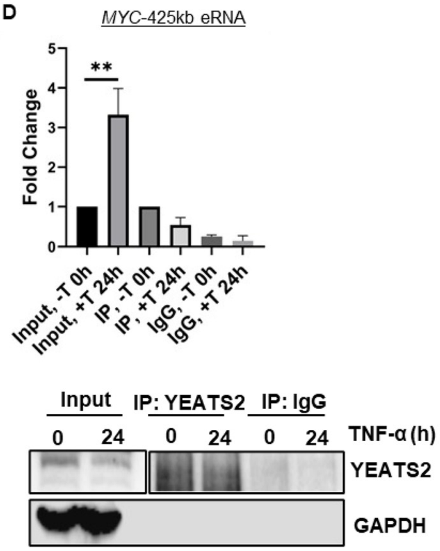

E

YEATS2 PROTEIN AA Sequence HUMAN:

MSGIKRTIKETDPDYEDVSVALPNKRHKAIENSARDAAVQKIETIIKEQFALEMKNKEHEIEVIDQRLIEARRMMDKL
RACIVANYYASAGLLKVSEGSKTCDTMVFNHPAIKKFLESPSRSSSPANQRAETPSANHSESDSLSQHNDFLSDKDN
NSNMDIEERLSNNMEQRPSRNTGRDTSRITGSHKTEQRNADLTDETSRLFVKKTIVVGNVSKYIPPDKREENDQST
HKWMVYVRGSRREPSINHFVKKVWFFLHPSYKPNDLVEVREPPFHLTRRGWGEFPVRVQVHFKDSQNKRIDIIHN
LKLDRTYTGLQTLGAETVVDVELHRHSLGEDCIYPQSSESDISDAPPSLPLTIPAPVKASSPIKQSHEPVPDTSVEKAGF
PASTEAERHTPFYALPSSLERTPTKMTTSQKVTFCSHGNSAFQPIASSCKIVPQSQVPNPESPGKSFQPITMSCKIVSG
SPISTPSPSPLPRTPTSTPVHVKQGTAGSVINNPYVIMDKQPGQVIGATTPSTGSPTNKISTASQVSQGTGSPVPKIHG
SSFVTSTVKQEDSLFASMPPLCPIGSHPKVQSPKPITGGLGAFTKVIIKQEPGEAPHVPATGAASQSPLPQYVTVKGG
HMIAVSPQKQVITPGEGIAQSAKVQPSKVVGVPVGSALPSTVKQAVAISGGQILVAKASSSVSKAVGPKQVVTQGVA
KAIVSGGGGTIVAQPVQTLTKAQVTAAGPQKSGSQGSVMATLQLPATNLANLANLPPGTKLYLTTNSKNPSGKGKLL
LIPQGAILRATNNANLQSGSAASGGSGAGGGGGGGGGGSGSGGGGSTGGGGGTAGGGTQSTAGPGGISQHLT
YTSYILKQTPQGTFLVGQPSPQTSGKQLTTGSVVQGTLGVSTSSAQGQQTLKVISGQKTTLFTQAAHGGQASLMKIS
DSTLKTVPATSQLSKPGTTMLRVAGGVITTATSPAVALSANGPAQQSEGMAPVSSSTVSSVTKTSGQQQVCVSQATV
GTCKAATPTVVSATSLVPTPNPISGKATVSGLLKIHSSQSSPQQAVLTIPSQLKPLSVNTSGGVQTILMPVNKVVQSFS
TSKPPAILPVAAPTPVVPSSAPAAVAKVKTEPETPGPSCLSQEGQTAVKTEESSELGNYVIKIDHLETIQQLLTAVVKKIP
LITAKSEDASCFSAKSVEQYYGWNIGKRRAAEWQRAMTMRKVLQEILEKNPRFHHLTPLKTKHIAHWCRCHGYTPP
DPESLRNDGDSIEDVLTQIDSEPECPSSFSSADNLCRKLEDLQQFQKREPENEEEVDILSLSEPVKINIKKEQEEKQEEV
KFYLPPTPGSEFIGDVTQKIGITLQPVALHRNVYASVVEDMILKATEQLVNDILRQALAVGYQTASHNRIPKEITVSNIH
QAICNIPFLDFLTNKHMGILNEDQ

F

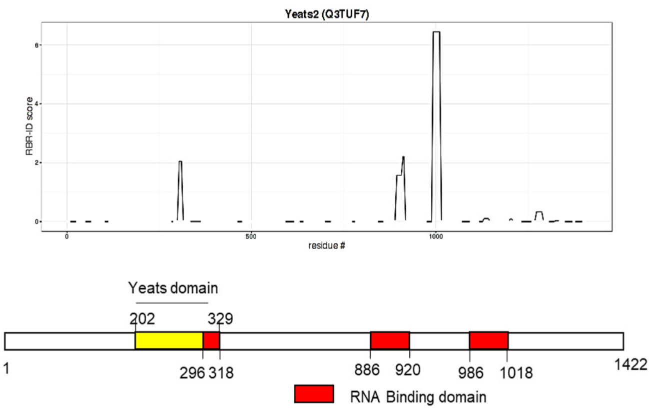

Figure EV3. *MYC*-490-kb eRNA interacts with YEATS2, a component of ATAC-HAT complex.

(**A**) Analysis by RPIseq software to check the interaction between *MYC*-490-kb enhancer RNA and different histone reader molecules. (**B**) Analysis by HDOCK web server tool to check the interaction between *MYC*-490-kb enhancer RNA and different histone reader molecules. (**C**) UV-RIP experiment was performed in MIAPaCa-2 cells in TNF-α stimulated condition and BRD4 associated *MYC*-490 eRNA was checked by qRT-PCR. Data are presented as mean ± SD from three independent experiments ($n = 3$). Statistical significance was determined using an unpaired two-tailed *t* test: ***$P = 0.0003$ for Input and ns for IP. Western blot was performed to check successful pulldown of BRD4 protein. GAPDH served as loading control. (**D**) UV-RIP experiment was performed in MIAPaCa-2 cells in TNF-α stimulated condition and YEATS2 associated *MYC*-425 eRNA was checked by qRT-PCR. Data are presented as mean ± SD from three independent experiments ($n = 3$). Statistical significance was determined using an unpaired two-tailed *t* test: **$P = 0.0037$ for Input. Western blot was performed to check successful pulldown of YEATS2 protein. GAPDH served as loading control. (**E**) Amino acid sequence of YEATS2 showing the Tyr residue (Red color) throughout the protein. The YEATS domain was marked with yellow color, and the three RNA binding domains were marked with purple color. (**F**) The three RNA binding motifs as shown in RBR-ID from Bonasio's lab (upper panel). A schematic of YEATS2 protein showing the overlapping region of YEATS domain with the 1st RNA binding motif (lower panel).

 © The Author(s)

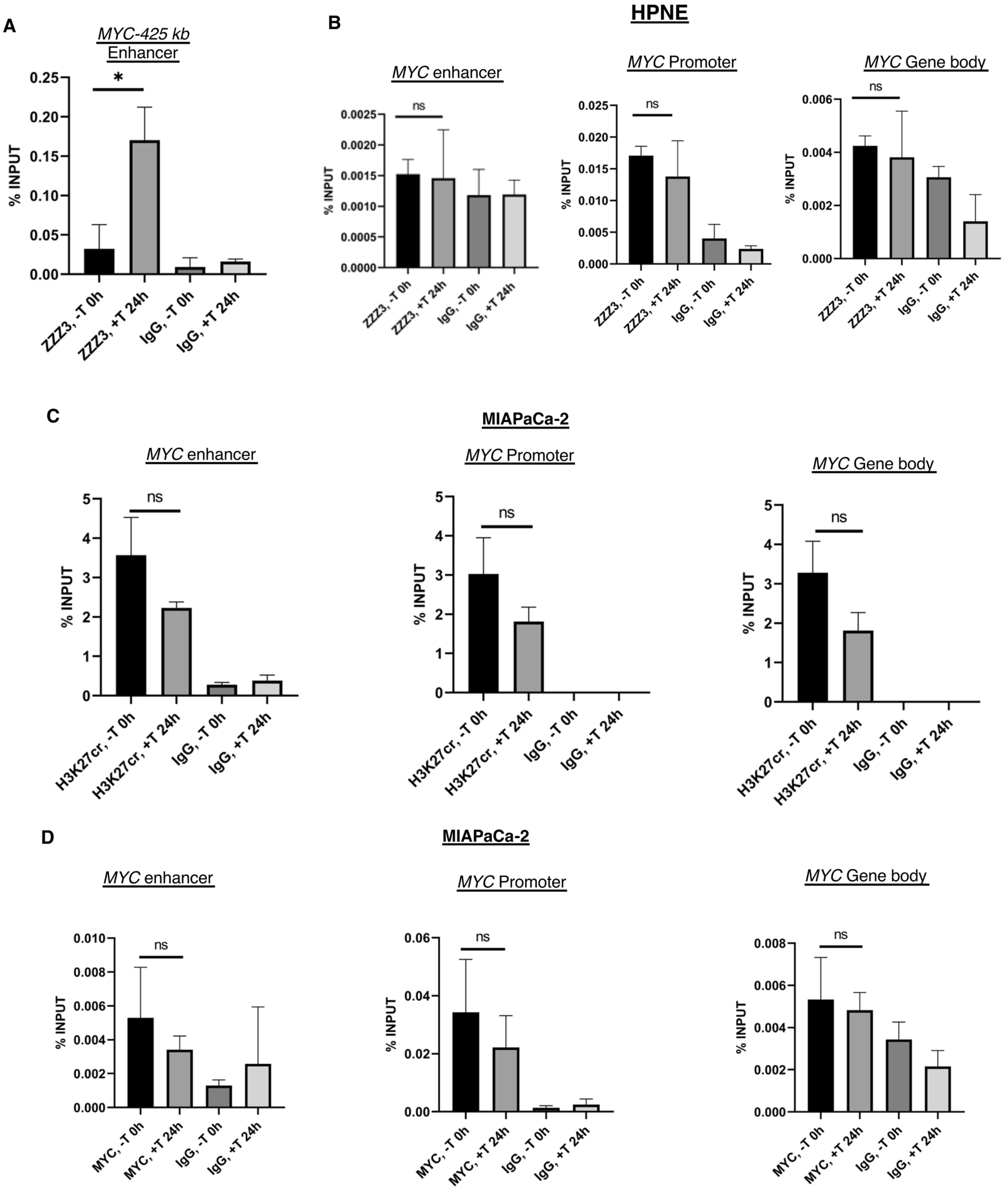
A
MYC-425 kb
Enhancer
*
% INPUT
ZZZ3, -T 0h
ZZZ3, +T 24h
IgG, -T 0h
IgG, +T 24h
B
HPNE
MYC enhancer
MYC Promoter
MYC Gene body
ns
C
MIAPaCa-2
MYC enhancer
MYC Promoter
MYC Gene body
H3K27cr, -T 0h
H3K27cr, +T 24h
D
MIAPaCa-2
MYC enhancer
MYC Promoter
MYC Gene body
MYC, -T 0h
MYC, +T 24h

◀ **Figure EV4. Neither MYC occupancy nor H3K27cr level was increased in *MYC* promoter/enhancer region with TNF stimulation.**

(**A**) ChIP-qPCR analysis was performed using ZZZ3 antibody to check YEATS2-containing ATAC complex occupancy in 24 h TNF-α stimulated condition at *MYC*-425-kb enhancer region. Data are presented as mean ± SD from three independent experiments ($n = 3$). Statistical significance was determined using an unpaired two-tailed t test: $^{*}P = 0.0146$ for Input. (**B**) There was no significant change in YEATS2 occupancy (measured by ZZZ3 antibody) at promoter region or enhancer region (490 kb upstream) as well as gene body of *MYC* gene between 0 h and 24 h of TNF-α treatment in HPNE cell line. (**C**) ChIP-qPCR study was performed using H3K27cr (crotonylation) antibody from TNF-α treated MIAPaCa-2 cells in both enhancer and promoter region as well as gene body. (**D**) Similar ChIP-qPCR was done with MYC antibody to assess MYC TF occupancy in MYC promoter and/or enhancer and also in gene body of 0 h and 24 h of TNF-α treated MIAPaCa-2 cells. The data represented the mean and s.e.m. of $n = 3$ independent experiments. Statistical significance was determined by a two-tailed Student's t test. ns, not significant.

 © The Author(s)
